# Design of a site selective heterochromic bimetallic lanthanide coiled coil with nanometre-scale control

**DOI:** 10.1039/d6sc00813e

**Published:** 2026-05-12

**Authors:** Louise N. Slope, Anokhi Shah, Michael J. Taylor, Valentina Borghesani, Simon G. Caulton, Nikolas J. Brooks, Kate A. Hadley, Georgina Rose, Robert I. Hunter, Hassane E. L. Mkami, Graham M. Smith, Aneika C. Leney, Niklaas J. Buurma, Andrew L. Lovering, Janet E. Lovett, Anna F. A. Peacock

**Affiliations:** a School of Chemistry, University of Birmingham Edgbaston B15 2TT UK a.f.a.peacock@bham.ac.uk; b SUPA School of Physics and Astronomy, University of St Andrews KY16 9SS UK; c BSRC, University of St Andrews KY16 9ST UK; d Department of Chemistry, Life Sciences, and Environmental Sustainability, University of Parma Parco Area Delle Scienze 17A 43124 Parma Italy; e School of Biosciences, University of Birmingham Edgbaston B15 2TT UK; f Physical Organic Chemistry Centre, School of Chemistry, Cardiff University Main Building Park Place Cardiff CF10 3AT Cymru/Wales UK

## Abstract

Lanthanide-protein scaffolds hold significant promise for the design of functional biomaterials. Yet the selective incorporation of multiple lanthanide ions with distinct properties into discrete sites at tuneable distances within a single construct remains a key challenge. Here, we report the rational design and structural characterization of the first *de novo* coiled coil capable of binding two different lanthanide ions at independent, non-equivalent sites with defined intermetallic spacing. By installing orthogonal coordination environments, comprising Asn_3_Asp_3_ and Asp_3_-only motifs, at defined positions along the coiled coil axis, we achieve precise, site-specific metal binding across a series of constructs spanning 1 to 5 nm. Site occupancy and intermetallic distances were validated using luminescence, electron paramagnetic resonance (EPR) spectroscopy, mass spectrometry and X-ray crystallography. The latter reveals the first structure of a coiled coil bound to two Tb^3+^ ions, and the shortest non-bridged metal–metal distance reported to date in such a scaffold (11.9 Å). The chemically distinct coordination sites enable sequential and selective metal loading. Remarkably, this system is capable of binding two different lanthanides, Tb^3+^ and Yb^3+^, at distinct sites, despite their extremely similar coordination chemistries. These results establish a robust and modular platform for constructing nanometre-scale molecular rulers, and highlight new avenues for the rational design of multifunctional metalloproteins.

## Abstract 2

A *de novo* protein scaffold enables selective binding of two different lanthanide ions at distinct sites with nanometre-scale control. This establishes a programmable platform for spatially defined metalloprotein architectures.

## Graphical abstract

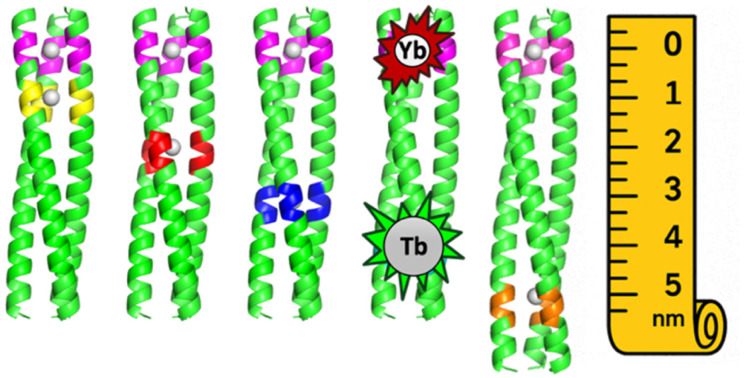

## Introduction

Lanthanide ions (Ln^3+^) play a central role in a broad spectrum of modern technologies, including biomedical imaging, molecular sensing, data storage, and renewable energy, due to their sharp f-f emission bands, long luminescence lifetimes, and unique magnetic properties. The ability to precisely control the spatial arrangement of Ln^3+^ ions opens the door to the rational design of next-generation optical, magnetic, and electronic materials. Despite these opportunities, the incorporation of multiple distinct Ln^3+^ ions into defined sites within biomolecular scaffolds remains a major challenge, primarily due to their remarkably similar ionic radii and coordination preferences. Achieving selective, site-specific binding of individual Ln^3+^ ions at tuneable distances within designed protein or peptide frameworks represents an unmet challenge.

Several natural examples are emerging as valuable blueprints for lanthanide protein design. In particular, the recent discovery of lanthanide-binding proteins such as lanmodulin^1^ have highlighted the emerging biological relevance of these metal ions and introduced promising leads for selective rare-earth capture and recovery,^2–6^ including through the formation of mixed lanthanide heterocomplexes.^7^ However, natural protein scaffolds often lack the structural modularity and offer limited control over the spatial organization of binding sites. In contrast, *de novo* designed coiled coils offer a highly programmable and structurally predictable platform for introducing bespoke metal-binding motifs at user-defined intervals along the helical axis.^8–12^ Their modularity enables precise tuning of both the coordination environment and the distance between bound metals, providing a versatile platform for constructing functional multi metalloproteins with atomic-level precision.

Previous studies have demonstrated that individual lanthanide-binding sites can be effectively incorporated into coiled coil protein scaffolds,^13^ enabling applications in MRI contrast enhancement and luminescent probes.^14–16^ Moreover, the local coordination environment, and thus the selectivity for specific Ln^3+^ ions, can be modulated by varying the position of the binding site along the coiled coil axis.^17,18^ Hydrated, solvent-exposed sites near the N-terminus tend to be promiscuous and accommodate a wide range of Ln^3+^ ions, whereas more buried core sites favour medium-sized ions such as Tb^3+^. Despite these advances, no system has yet been reported that integrates two distinct lanthanide-binding sites within a single scaffold.

Here, we report the first example of a heterochromic dual-site lanthanide-binding coiled coil scaffold capable of coordinating two different Ln^3+^ ions at non-equivalent, spatially separated sites. Through a combination of luminescence spectroscopy, EPR distance measurements, mass spectrometry and X-ray crystallography, we demonstrate that this scaffold can both differentiate between closely related Ln^3+^ ions and precisely control their spatial arrangement, positioning them with predictable intermetallic distances ranging from 1 to 5 nm. These findings establish new principles for engineering functional multi metalloproteins with tuneable coordination chemistry and nanoscale spatial precision, and offer a versatile platform for advancing synthetic biology and lanthanide biochemistry.

## Results

### Design of a dual lanthanide-binding coiled coil

To achieve the goal of creating a coiled coil capable of coordinating two distinct lanthanide ions, Ln_1_ and Ln_2_, at two separate yet similar binding sites, a series of peptides were designed by incorporating two lanthanide binding sites into a single coiled coil. These dual-binding coiled coils feature two distinct types of lanthanide-binding sites: a promiscuous site near the N-terminus (top) of the coiled coil, characterized by a single Asp_3_ layer; and a selective site located more buried centrally in the core, composed of adjacent Asn_3_Asp_3_ layers, which enables size-dependent discrimination between Ln^3+^ ions.^18^ The more buried core Asn_3_Asp_3_ sites show a preference for medium-sized Ln^3+^ ions, typically those with ionic radii between those of Nd^3+^ and Tb^3+^.^18^ To assist in monitoring and quantifying binding, Trp residues were introduced adjacent to the lanthanide-binding sites. Tryptophan facilitates concentration measurements and can sensitize Tb^3+^ luminescence when in close proximity, serving as a useful reporter of binding. Because incorporating two lanthanide-binding sites within a single coiled coil is expected to destabilize the structure, the peptide was extended by a heptad to restore stability.

**Fig. 1:**
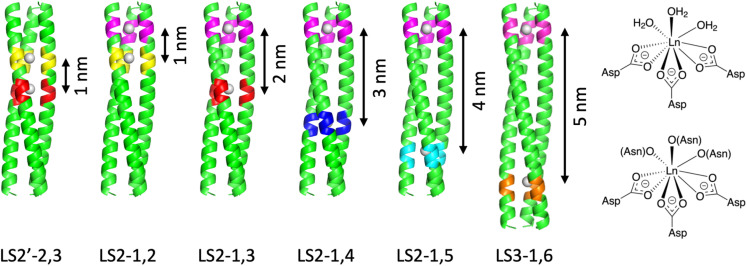
Models of the series of dual Ln^3+^ binding coiled coils containing adjacent (LS2′-2,3 and LS2-1,2) and distinct (LS2-1,3, LS2-1,4, LS2-1,5 and LS3-1,6) sites. Constructed from Asp_3_ and Asn_3_Asp_3_ sites (right). The main chain atoms are represented as helical ribbons (N-terminus at the top, C-terminus at the bottom), binding sites are highlighted, and the Ln^3+^ ions are shown as grey spheres. Distances between the binding sites are shown.

The peptide series, LS2-*X*,*Y*, were designed using the heptad (*abcdefg*) repeat approach, based on the sequence Ac-G(I_*a*_A_*b*_A_*c*_I_*d*_E_*e*_Q_*f*_K_*g*_)_5_G-NH_2_. In this design, the repeating heptad motif ensures residues occupying equivalent positions (*e.g.* position *a*) in each heptad align with similar spatial environments due to the periodicity of the α-helical structure. *X* corresponds to the location of the top binding site (typically position 1 at the interface of the 1st and 2nd heptad) and *Y* to the location of the second binding site (see Table 1 and Fig. 1, S1–S8). For example, the second Asn_3_Asp_3_ site can span the 2nd and 3rd heptads, adjacent to the first site (as in LS2-1,2) or can be systematically translated linearly down the helix one heptad at a time, to yield LS2-1,3, LS2-1,4 and LS2-1,5, respectively. LS2-1,5 features binding sites at both the N- and C-termini. A longer version of this peptide, LS3-1,6, has been extended by one core heptad. In LS2-1,2 the terminal and buried core metal binding sites are “adjacent” with no intervening Ile layers between the three binding layers, *i.e.* creating a double binding site Asp_3_Asn_3_Asp_3_. Similarly, two buried core binding sites were located “adjacent” in LS2′-2,3, which consists of four contiguous binding residue layers, Asn_3_Asp_3_Asn_3_Asp_3_, with no intervening Ile residues.

**Table 1:** Peptide sequences used in this work

Peptide	Sequence^a^ (N → C terminus)								
Heptad	g	abcdefg	abcdefg	abcdefg	abcdefg	abcdefg	abcdefg	abcdefg	a
LS2-1,2	Ac-G	IAAIE**W̲**K	**D̲**AA**N̲**E**W̲**K	**D̲**AAIEQK	IAAIEQK	IAAIEQK	IAAIEQK	G-NH_2_	
LS2-1,3	Ac-G	IAAIE**W̲**K	**D̲**AAIEQK	IAA**N̲**E**W̲**K	**D̲**AAIEQK	IAAIEQK	IAAIEQK	G-NH_2_	
LS2-1,4	Ac-G	IAAIE**W̲**K	**D̲**AAIEQK	IAAIEQK	IAA**N̲**E**W̲**K	**D̲**AAIEQK	IAAIEQK	G-NH_2_	
LS2-1,5	Ac-G	IAAIE**W̲**K	**D̲**AAIEQK	IAAIEQK	IAAIEQK	IAA**N̲**E**W̲**K	**D̲**AAIEQK	G-NH_2_	
LS2-1,5(7Q)	Ac-G	IAAIEQK	**D̲**AAIEQK	IAAIEQK	IAAIEQK	IAA**N̲**E**W̲**K	**D̲**AAIEQK	G-NH_2_	
LS2-1,5(35Q)	Ac-G	IAAIE**W̲**K	**D̲**AAIEQK	IAAIEQK	IAAIEQK	IAA**N̲**EQK	**D̲**AAIEQK	G-NH_2_	
LS3-1,6	Ac-G	IAAIE**W̲**K	**D̲**AAIEQK	IAAIEQK	IAAIEQK	IAAIEQK	IAA**N̲**E**W̲**K	**D̲**AAIEQK	G-NH_2_
LS2′-2,3	Ac-G	IAAIEQK	IAA**N̲**E**W̲**K	**D̲**AA**N̲**E**W̲**K	**D̲**AAIEQK	IAAIEQK	IAAIEQK	G-NH_2_	
*x*LS2″-2,3	Ac-E	**W̲**EAIEKK	IAA**N**ESK	**D̲**QAIEKK	**D̲**QAIEKK	IQAIEKK	IEAIEHG	IG-NH_2_	

a Binding site residues, and where relevant the Trp (sensitizer), are bold and underlined.

### Tb^3+^ titrations

**Fig. 2:**
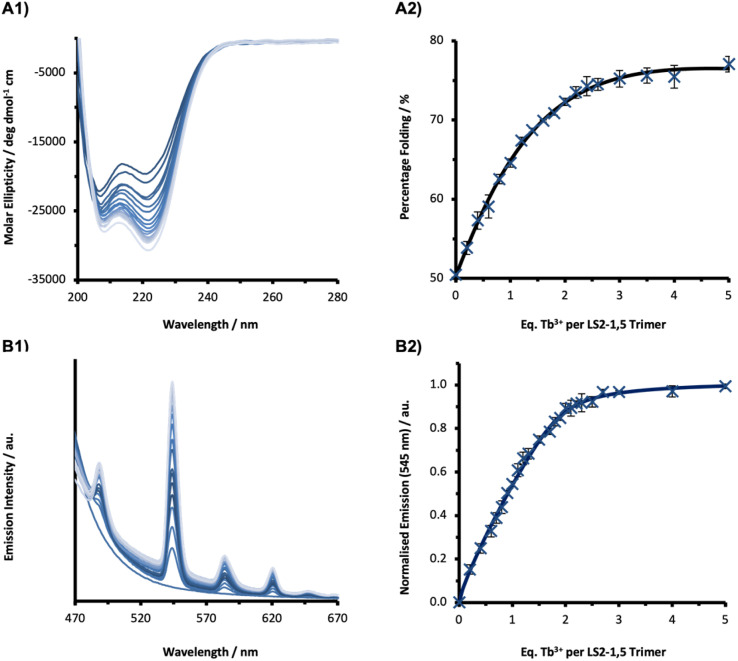
Tb^3+^ binding titrations monitored by (A1) CD and (B1) luminescence of a 30 µM LS2-1,5 monomer solution in 10 mM HEPES buffer pH 7.0. Plot of Tb^3+^ binding, showing (A2) percentage folded (based on molar ellipticity at 222 nm), and (B2) normalized emission intensity (based on the integration of the 545 nm Tb^3+^ emission peak), as a function of Tb^3+^ equivalents per trimer. Data is a result of the average of three repeats where the error bars represent the standard deviation. *λ*_ex_ = 280 nm.

The series of peptides were investigated by circular dichroism (CD) spectroscopy. Spectra of peptides in the absence and presence of different concentrations of Tb^3+^ ions are shown in Fig. 2A and S9. The extent of peptide folding, based on the molar ellipticity at 222 nm (an indication of α-helical content) varies across the series (Table S1). Generally, those peptides with binding sites located within core heptads, and shorter segments of uninterrupted heptads, are poorly folded in the absence of a Tb^3+^, whereas those with both binding sites located in terminal heptads, are more folded. However, in all examples, the addition of increasing aliquots of TbCl_3_ led to an increase in folding, see Fig. 2A and S9, before reaching a plateau at a molar ratio consistent with around two equivalents Tb^3+^ per trimer.

The same titration monitored by luminescence spectroscopy with excitation of the Trp residue at 280 nm, led to the appearance of the characteristic Tb^3+^ emission peaks at 490, 545, 585, 620, and 650 nm, on addition of increasing aliquots of TbCl_3_ (see Fig. 2B and S10). Though some of the CD titrations revealed biphasic profiles (Fig. S9), this behaviour was more clearly resolved through luminescence spectroscopy (Fig. S10). Titrations were consistent with two binding sites and reached a plateau at around two equivalents Tb^3+^ per trimer.

### Control peptides

**Fig. 3:**
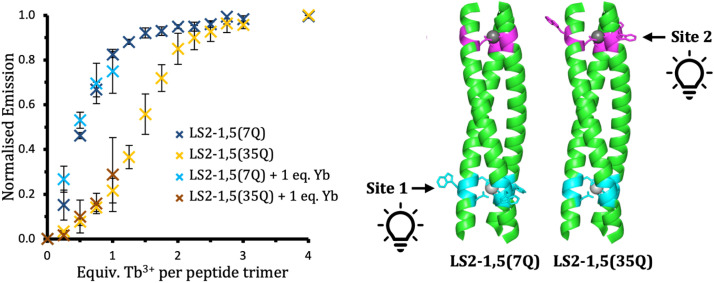
Normalized emission intensity based on the integration of the 545 nm Tb^3+^ emission peak of 30 µM peptide monomer solutions of LS2-1,5(7Q) (blue) and LS2-1,5(35Q) (yellow/red) in 10 mM HEPES buffer pH 7.0, as a function of Tb^3+^ equivalents per trimer in the absence (dark blue and yellow) and presence of one equivalent of Yb^3+^ (light blue and red). Data are the average of three repeats where the error bars represent the standard deviation. *λ*_ex_ = 280 nm.

Instead of being limited to observing overall luminescence intensity of all bound Tb^3+^, our coiled coil allows us to design control structures where each binding site can be monitored individually, providing further insight into the binding equilibria and thus the applicable binding model. Two “control” peptides based on LS2-1,5 have been prepared, LS2-1,5(7Q) and LS2-1,5(35Q), where *X*Q corresponds to the Trp position in the original amino acid sequence which has been changed to a Gln (see Table 1, Fig. 3 and S11). Whereas both binding sites in LS2-1,5 feature adjacent Trp reporter groups, the single Trp in the control peptides can only report on the binding of Tb^3+^ to one site, as the second site is too far away (∼4 nm) for Tb^3+^ emission to be sensitized by Trp excitation at 280 nm.

The two control peptides, LS2-1,5(7Q) and LS2-1,5(35Q), displayed similar Tb^3+^ binding curves when monitored by CD, to each other and the parent peptide LS2-1,5, and suggest two Tb^3+^ ions bind per trimer (Fig. S12). The analogous Tb^3+^ titrations monitored by luminescence differ from each other and from LS2-1,5 (Fig. 3 and S13). Firstly, the final emission intensity for Tb^3+^ bound to LS2-1,5(35Q) is less than that for LS2-1,5(7Q). Secondly, the titration for LS2-1,5(7Q), shows a sharp increase in luminescence on the addition of up to one equivalent of Tb^3+^, followed by a plateau at greater equivalences. The analogous titration with LS2-1,5(35Q), shows the greatest increase between one and two equivalences of Tb^3+^, followed by a plateau.

To obtain quantitative data from these titrations, a fitting function was developed that allows the global fit of a binding model to the luminescence titration data for Tb^3+^ binding to LS2-1,5, LS2-1,5(7Q) and LS2-1,5(35Q). The binding model (Scheme S1) was based on the model by Tochtrop *et al.*^19^ and involves two non-equivalent binding sites on each coiled coil and allows for cooperativity, if any. The model further assumes that luminescence for LS2-1,5(7Q) only reports on occupancy of site 1, that luminescence for LS2-1,5(35Q) only reports on occupancy of site 2, and that luminescence for LS2-1,5 corresponds to the simple sum of luminescence intensities from sites 1 and 2. The model also allows for luminescence quantum yields to be different for each site. A full derivation of the equations and Origin C code defining the model are available in the SI. Overall, global fits of the Tb^3+^ binding model to all titration curves with fully optimizable background signals for each individual titration with the cooperativity factor restricted to values of 1, 0.1 and 10, are shown in Fig. S14–S16, fitting parameters for cooperativity factors restricted to values of 0.01, 0.1, 1, 10 and 100 are reported in Table S2, and an evaluation of the significance of parameter values is represented in Fig. S17. Overall, the analysis indicates an association constant of 1.1 × 10^7^ M^−1^ for site 1 and 5 × 10^5^ M^−1^ for site 2 with no evidence for cooperativity in binding. A speciation plot generated from this binding model using the derived association constants is shown in Fig. S18. The predicted population distributions are in excellent agreement with the experimental titration data.

### Hydration states of bound Tb^3+^

Luminescence lifetime decay studies of the dual binding site coiled coils were performed in the presence of 0.3 equivalents TbCl_3_, in H_2_O and D_2_O, and monitored at 545 nm, see Fig. S19–S20. The inner sphere water content coordinated to the bound Tb^3+^ was estimated using the Parker–Beeby equation^20^ and is reported in Table S3. Analogous data recorded for 0.3 equivalents TbCl_3_ in the presence of 1.0 equivalents YbCl_3_ per trimer (*vide infra*) is consistent, with the majority of Tb^3+^ remaining bound in sites with a similar inner sphere water content.

### Gd^3+^ binding

Due to their similar size but different properties, Tb^3+^ and Gd^3+^ are frequently used interchangeably. Titrations of GdCl_3_ into 30 µM solutions of LS2-1,3, LS2-1,5 and LS2′-2,3 monomer, *i.e.* examples of dual Ln binding peptides with both distinct and adjacent sites, were monitored by CD spectroscopy. Titrations are shown in Fig. S21, and are in good agreement with the analogous CD titrations performed with TbCl_3_.

### EPR distance measurements

**Fig. 4:**
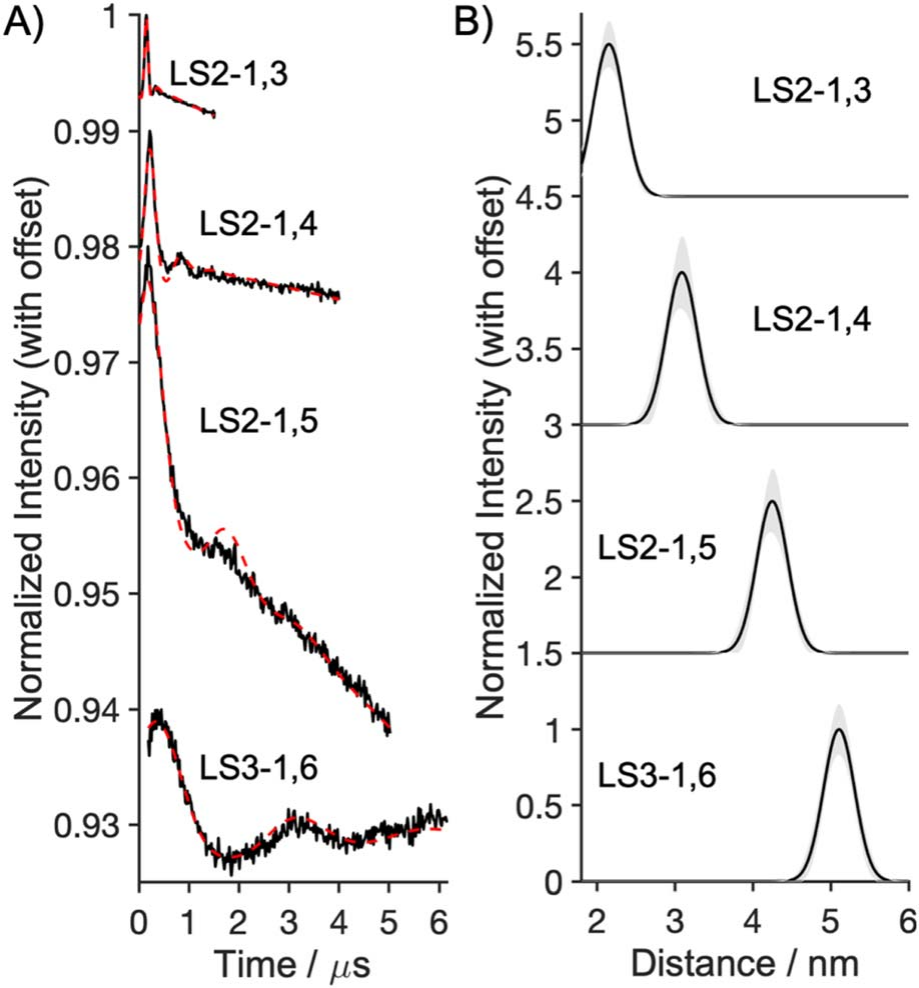
The DEER results for Gd_2_(Peptide)_3_ with fits assuming a Gaussian distribution of fixed standard deviation from the mean of 0.2 nm: (A) time traces (black) and simulated fits (red dash); (B) distance distributions with 95% confidence shown in grey.

The distance between the two bound Gd^3+^ for the dual binding peptides was measured using the EPR spectroscopic method of double electron–electron resonance (DEER) in the frozen state.^21–24^ The LS2 measurements were taken at W-band using a specialized home-built high power and wide bandwidth spectrometer (HiPER) in order to obtain accurate distances even for the expected short Gd–Gd distance in the LS2-1,3 peptide.^25,26^ The DEER time traces and the distances shown in Fig. 4 were extracted using DeerLab 1.1.4.^27^Table 2 presents the mean distance from the constrained-width Gaussian analysis, and the most probable distance obtained from Tikhonov regularization analysis, *i.e.* a non-parametric or “model free” method. The distances support the hypothesis that the peptide series binds Gd^3+^ at specific sites with inter Gd^3+^ distances of slightly over 2, 3, 4 and 5 nm for LS2,1–3, LS2-1,4, LS2-1,5 and LS3-1,6, respectively (see Fig. S22–S29 and Tables S4–S8).

**Table 2:** The DEER measurement Gd–Gd distance results

Gd_2_(Peptide)_3_	Mean of constrained Gaussian (nm)	Most probable distance from model-free fit (nm)
LS2-1,3	2.15	2.05
LS2-1,4	3.09	3.07
LS2-1,5	4.25	4.18
LS3-1,6	5.11	5.09

### X-ray crystal structure

**Fig. 5:**
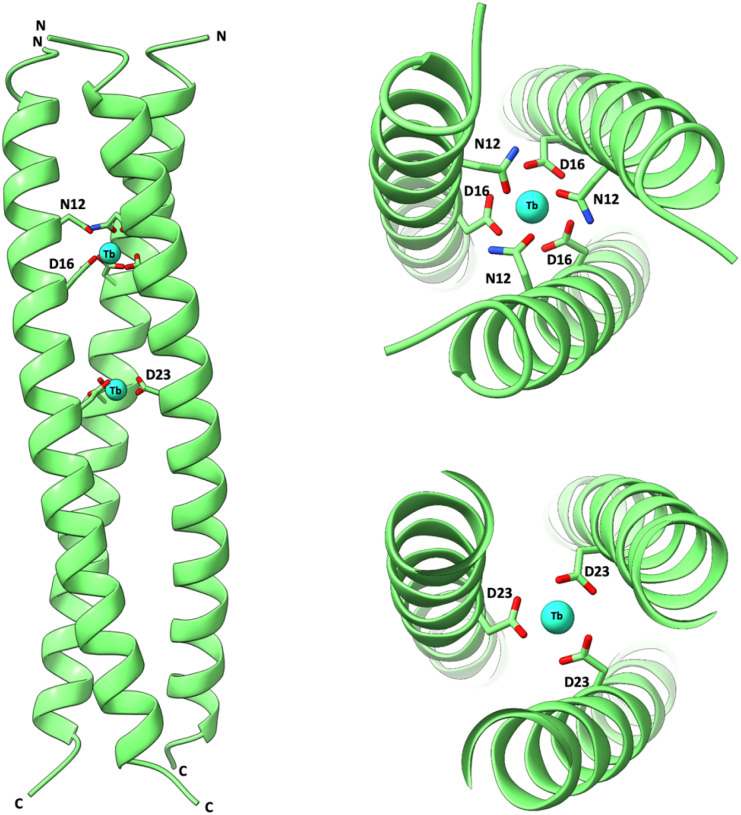
Crystal structure of two Tb^3+^ ions bound within the interior of a designed parallel three stranded coiled-coil (*x*LS2″-2,3). Main-chain atoms are shown as ribbons (N-termini indicated), the binding-site Asn and Asp side chains in stick form (C green, O red, N blue) and the bound Tb^3+^ ions as cyan spheres. Left: side view of the full assembly. Right: zoomed-in views of individual Tb^3+^ binding sites, showing the coordination environment formed by ligating residues. Due to the limited resolution (2.76 Å), the exact coordination geometry could not be unambiguously determined from the crystallographic data.

Despite multiple attempts, we were unable to obtain crystals of the LS peptides of suitable quality for X-ray diffraction. We have previously overcome this challenge, inspired by the success of CoilSer and related peptides,^18,28,29^ by modifying the exterior of the coiled coil to aid in crystal–crystal packing. We reasoned that (1) buried core binding site might be less dynamic, and (2) that design modifications that further stabilize the structure should enhance our chances of success. *x*LS2″-2,3 features two Tb^3+^ sites which are both buried (based on LS2′-2,3), but a stabilizing Ile layer is introduced in place of the intervening Asn (position 19). Crystals were obtained in the presence of Tb^3+^ in the *P*6 space group and the structure solved to 2.76 Å resolution, see Fig. 5 and Tables S9. The location of Tb^3+^ ions within the distinct Asn_3_Asp_3_ and Asp_3_ binding sites and at coiled coil interfaces are shown in Fig. S30 and S31, respectively.

### Yb^3+^ binding

To explore discrimination between different sized Ln^3+^ ions, titration of the smaller YbCl_3_ into a 30 µM solution of LS2-1,5 was monitored by CD spectroscopy. Yb^3+^ and Tb^3+^ were selected as representative smaller and optimally sized lanthanide ions, respectively, based on our previous studies in which related mono-binding-site coiled coils showed the greatest discrimination between these size regimes.^18^ The addition of increasing concentrations of Yb^3+^ led to a steady, almost linear, increase in folding (based on molar ellipticity at 222 nm) from around 50 to 65% at five equivalents Yb^3+^ per LS2-1,5 trimer, see Fig. S31. This differs from the analogous titration performed with Tb^3+^, for which the molar ellipticity at 222 nm at five equivalents Tb^3+^ per LS2-1,5 trimer, has reached a plateau consistent with 77% folding. The addition of 1 equivalent Yb^3+^ followed by the addition of increasing aliquots of Tb^3+^, closely mirrors the Tb^3+^ titration in the absence of Yb^3+^, see Fig. 3 and S32.

The displacement of bound Tb^3+^ from LS2-1,5 by the smaller Yb^3+^ was investigated using luminescence spectroscopy. Displacement plots, showing the decrease in Tb^3+^ luminescence at 545 nm with increasing equivalence of YbCl_3_, are presented in Fig. S33. Time-course measurements confirm that equilibration is rapid under these conditions (Fig. S33). These data are compared with the emission intensity of LS2-1,5 in the presence of either 1 or 2 equivalents TbCl_3_ per trimer. Although a reduction in emission intensity is observed upon Yb^3+^ addition, complete displacement of both Tb^3+^ ions does not occur even after the addition of 100 equivalents YbCl_3_ (Fig. S33). Analysis of the displacement titration in terms of the mathematical expression describing competitive binding developed by Wang^30,31^ (Fig. S33) yields a binding constant *K*_a_ for Yb^3+^ binding to site 2 of 2.7 × 10^4^ M^−1^.

### Mass spectrometry analysis of metal binding

Native mass spectrometry (MS) was employed to characterize the metal-binding behaviour of the bimetallic assemblies. Solutions containing 90 µM LS2-1,5 monomer (corresponding to 30 µM trimer) exhibited spectra dominated by a charge-state envelope consistent with monomeric species; however, low-abundance peaks attributable to the intact trimer were also observed (Fig. S34 and S35A).

Upon addition of 0.5 equivalents of TbCl_3_ (15 µM), a new set of peaks corresponding to the monometallated complex Tb(Peptide)_3_ emerged (Fig. S35B) that increased in intensity upon addition of 1.0 equivalent of TbCl_3_ (30 µM). At one equivalent of Tb^3+^, the unmetallated trimer remained readily detectable, although at lower abundance than the monometallated complex, and a minor peak consistent with the bimetallic complex Tb_2_(Peptide)_3_ was observed at very low intensity (Fig. S35C).

An analogous experiment performed with 1.0 equivalent of YbCl_3_ per trimer produced similar complexes, including the unmetallated trimer, Yb(Peptide)_3_, and Yb_2_(Peptide)_3_. In contrast to Tb^3+^, the unmetallated trimer was the most abundant complex in the presence of Yb^3+^, indicating weaker overall binding under these conditions (Fig. S35D).

**Fig. 6:**
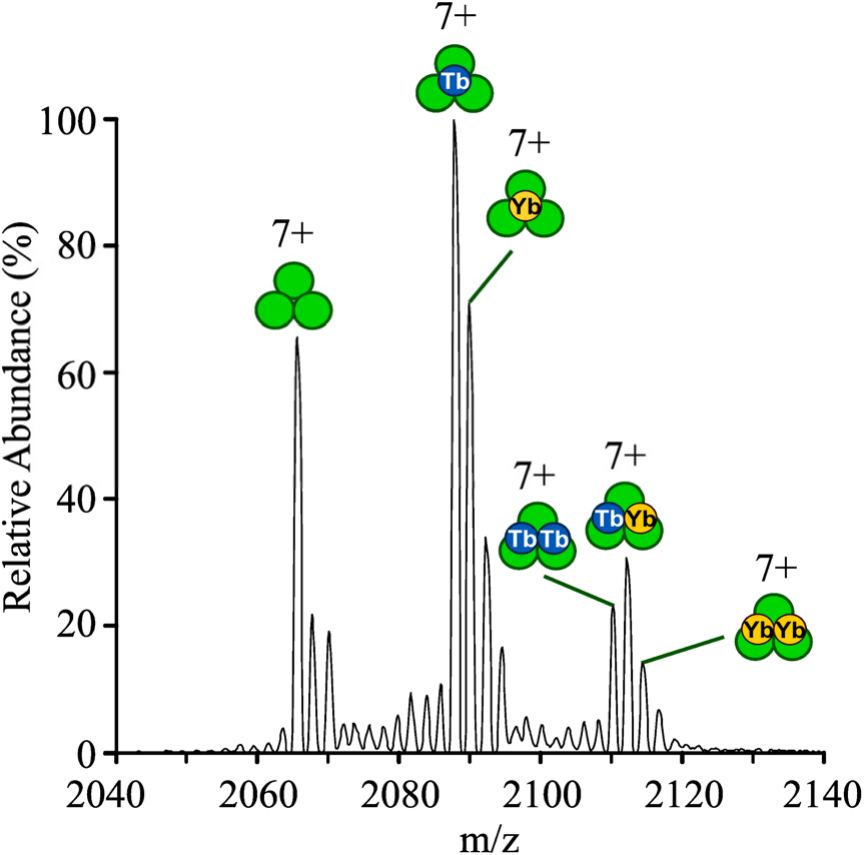
Native mass spectrum of LS2-1,5 following incubation with 1.0 equiv. of Tb^3+^ and Yb^3+^ per trimer. An expanded view of the 7+ charge state is shown. Peptide monomers, Tb, and Yb are indicated by green, blue, and yellow circles, respectively.

When spectra were acquired in the presence of both metals (1.0 equivalent each of Tb^3+^ and Yb^3+^), signals corresponding to the unmetallated trimer, the monometallated Tb(Peptide)_3_ and Yb(Peptide)_3_ complexes, and bimetallic species were detected. Among the monometallated complexes, Tb(Peptide)_3_ was more abundant than its Yb analogue. The bimetallic complexes included the homobimetallic Tb_2_(Peptide)_3_ and Yb_2_(Peptide)_3_ complexes, as well as the heterobimetallic YbTb(Peptide)_3_ species, the latter being the most abundant of the three (Fig. 6 and S35E, Table S10).

Even under conditions of excess Tb^3+^ (2.0 equivalents of Tb^3+^ and 1.0 equivalent of Yb^3+^ per trimer), such that both binding sites could be saturated by Tb^3+^, the heterobimetallic YbTb(Peptide)_3_ complex remained clearly detectable, despite an overall increase in the abundance of Tb-containing species (Fig. S35F).

## Discussion

### Accessing folded coiled coils bound to two Ln^3+^ ions

The binding of Tb^3+^ to a series of six coiled coils, each designed to coordinate two Ln^3+^ ions in distinct locations, was initially investigated by CD spectroscopy. Despite the destabilizing effect typically associated with introducing two Ln^3+^-binding sites into the hydrophobic core, folding could be recovered in all constructs by extending the sequence with an additional (sixth) heptad. This design adjustment generally yielded well-folded coiled coils upon full metalation. CD titrations across the series indicated binding of two equivalents of Tb^3+^ per coiled coil trimer.

### Impact of site location on folding and Ln^3+^ coordination chemistry

Across the coiled coil series, we systematically varied the position of Ln^3+^-binding sites to alter the inter-metal distance (*vide infra*). Notably, isomeric sites, defined by identical amino acid compositions placed at equivalent heptad positions (*abcdefg*), exhibited marked differences in folding stability depending on their location within the coiled coil. As expected, binding sites embedded within core (buried) heptads were more destabilizing than those placed within terminal heptads.^17,32^ Scaffolds containing exclusively buried sites, such as LS2′-2,3, were the most destabilized, while those exclusively featuring terminal sites, including LS2-1,5 and LS3-1,6, showed the highest degree of folding. The additional seventh heptad in LS3-1,6, introduced to accommodate longer Ln–Ln distances, provides the anticipated enhancement in folding.^33^

An apparent exception to these trends is the LS2-1,2 construct, which incorporates three adjacent binding layers (Asp_3_Asn_3_Asp_3_) near the N-terminus. Despite being constructed from an adjacent terminal and buried site, this arrangement is well tolerated and may be better described as an extended terminal site. In contrast, the LS2′-2,3 peptide, which positions two adjacent buried binding sites deeper within the core (Asn_3_Asp_3_Asn_3_Asp_3_), is significantly destabilized. Even upon full Ln^3+^ coordination, folding is not fully restored.

Luminescence studies, including those using control peptides, revealed that Tb^3+^ sites can exhibit markedly different emission characteristics. Sites that are more hydrated or solvent-accessible tend to exhibit weaker emission, whereas those that are more buried and coordinatively saturated show enhanced luminescence, consistent with previously reported trends.^17^

It remains unclear whether the coordination environment of a given site can be modulated by the presence of a second binding site. In the crystal structure of *x*LS2″-2,3, the Tb^3+^-bound Asn_3_Asp_3_ site retains the same coordination geometry as in its mono-metallic counterpart (Fig. S36), despite the proximity (∼1 nm) of a second Tb^3+^ site. In contrast, the C-terminal site in LS2-1,5 (site 1), derived from the previously characterized MB1-4 construct,^17^ does not replicate the coordination behaviour of its mono-metallic analogue. Specifically, luminescence decay experiments reveal that while MB1-4 yields a hydrated Tb(OH_2_)_2_ coordination state, the analogous site in LS2-1,5 exhibits features of a coordinatively saturated environment. This may reflect stabilization from the additional heptad.

### Binding two Ln^3+^ ions at defined distances: coiled coils as molecular rulers

The modular and predictable architecture of *de novo* designed coiled coils enables the precise placement of two Ln^3+^ ions at defined positions along the helical axis, effectively transforming these constructs into nanoscale molecular rulers. To validate these distances, we employed pulsed EPR spectroscopy to measure the separation between two paramagnetic Gd^3+^ ions bound at designated sites. The observed distances align closely with those predicted by structural models of the LS2-1,*X* to LS3-1,6 series.

The shortest accessible distance that DEER can measure is limited by the physical attributes of the sample and the spectrometer hardware.^23,24,34^ Therefore, while to the best of our knowledge, the 2 nm distance in Gd_2_(LS2-1,3)_3_ is the shortest Gd–Gd DEER measurement to date, in a non-model system, the shorter intermetallic distances could not be accurately resolved for binding sites separated by a single heptad. To experimentally validate these shorter distances, we successfully crystallized a related coiled coil containing two Tb^3+^ ions, offering a complementary structural approach. While X-ray crystal structures of coiled coils or helical bundles featuring multiple metal ions have been reported (including dinuclear sites,^35–37^ multi-metal clusters,^38,39^ and distinct transition metal sites^10,12,40^), this structure represents the first example of a coiled coil simultaneously coordinating two Ln^3+^ ions and at separate, non-equivalent sites.

The measured Tb–Tb distance of 11.9 Å is in good agreement with the expected spacing from design (Fig. 1 and 4). An intervening Ile residue between the two binding layers, absent in related constructs such as LS2′-2,3, is believed to enhance overall structural stability and may position the central Tb^3+^ more within the Asp layer. Models predict that replacing this Ile with an Asn would shift the second Tb^3+^ towards the N-terminus, reducing the intermetallic distance to ∼10.5 Å (based on Asp–Asp *C*_α_ backbone distances).

To our knowledge, the 11.9 Å spacing represents the shortest crystallographically observed distance between two distinct, non-equivalent metal-binding sites within a coiled coil or helical bundle (excluding cases with shared ligands) and the only example of the same metal ion bound at two structurally distinct, non-overlapping sites in such a scaffold.

By combining EPR spectroscopy with X-ray crystallography, we have demonstrated the ability to construct a family of molecular rulers spanning 1–5 nm with 1 nm resolution. This level of spatial precision in metal site placement represents a powerful tool for engineering next-generation metalloproteins.

### Structural characterization of distinct binding sites

One Tb^3+^ is coordinated at an Asn_3_Asp_3_ site near the N-terminus of the coiled coil, closely resembling that in our previously reported mono-metallic structure. Whereas the second Tb^3+^ is bound at an Asp_3_-only site located more centrally within the coiled coil. Although this site was examined previously, crystallographic characterization was not possible due to poor crystallizability when positioned near the N-terminus (CS1-1).^17^ Relocating the site to a more central position within the coiled coil has now enabled the first structural characterization of this second distinct Ln^3+^ binding site.

### Sequential and selective Tb^3+^ binding in a heterochromic coiled coil

We next investigated whether selective and sequential binding could be achieved between distinct metal-binding sites. While some CD titrations exhibited biphasic profiles indicative of sequential site occupancy, this behaviour was more clearly resolved through luminescence spectroscopy. The non-linear luminescence titration profiles arise from the distinct luminescence properties of individual Tb^3+^ binding sites. A notable exception is LS2′-2,3, which lacks distinguishable luminescence features, likely due to both Tb^3+^ ions occupying similar, coordinatively saturated, buried sites, that are expected to be similarly emissive.

Site specific binding was investigated using the LS2-1,5 construct and its control variants, LS2-1,5(7Q) and LS2-1,5(35Q), which differ in the placement of Trp reporter residues. These designs enable selective sensitization of Tb^3+^ at each of the two binding sites, allowing for independent monitoring of site occupancy throughout the titration experiments (Fig. 3). The titrations are consistent with the first equivalent of added Tb^3+^ predominantly binding to site 1 (C-terminal Asn_3_Asp_3_), with only partial occupancy of site 2 (N-terminal Asp_3_). The second equivalent of added Tb^3+^ then predominantly binds to site 2. Overall, this results in Tb^3+^ ions coordinating two chemically distinct sites, resulting in divergent spectroscopic properties.^8^

Global analysis of the LS2-1,5 titrations shows that both binding sites can be treated as separate sites with association constant of Tb^3+^ for binding site 1 being over an order of magnitude higher than for binding site 2. Cooperativity is not required to describe the data. However, we note that the model does not exclude the possibility that the Tb^3+^ association constant for site 2 is affected by Tb^3+^ binding to site 1. Any such effect is insufficient to result in cooperative binding.

These studies have all been performed at pH 7, however, lanthanide ion association constants are likely to be strongly influenced by pH, and this will be investigated in the future. However, lanthanide ion association constants for native and other *de novo* proteins, tend to be reported at pH 7, unless being investigated for lanthanide separation technologies. Our reported values range from 10^4^ to 10^7^ M^−1^, which is consistent with those for isolated EF-hand motifs and some *de novo* scaffolds.^41^ While these values represent significant binding, they do not yet achieve the exceptionally high association constants seen in natural lanmodulin (LanM) and designed TIM barrel protein scaffolds, which can exceed 10^11^ and 10^13^ M^−1^.^42,43^

Bimetallic coiled coils with two distinct metal-binding sites have been reported previously,^8–10,44,45^ including one example incorporating Tb^3+^ and Hg^2+^.^11^ The present work, however, establishes the first coiled coil scaffold capable of binding two Ln^3+^ ions, and does so at independent, non-equivalent sites within a, single, well-defined assembly. This behaviour echoes heterochromic Cd^2+^ coiled coils, where binding order is dictated by changes in the resulting coordination number and geometry arising from different exogenous water coordination.^8,9^ In contrast, the Ln^3+^ system described here likely retains similar coordination number and geometries across the two sites, despite differences in first coordination sphere ligands.

### Heterobimetallic lanthanide coiled coil

The combined spectroscopic, displacement, and native MS data reveal hierarchical and site-selective lanthanide binding to LS2-1,5. Tb^3+^ initially binds to site 1, which has the higher association constant and is size-selective and coordinatively saturated, to form Tb_(1)_(LS2-1,5)_3_. Only upon further metal addition does Tb^3+^ bind to site 2, which has the lower association constant and is more size-promiscuous, yielding the fully loaded Tb_(2)_Tb_(1)_(LS2-1,5)_3_ complex.

Importantly, the presence of Yb^3+^ does not perturb this binding sequence. Tb^3+^ titrations performed in the absence and presence of Yb^3+^ show nearly identical CD and luminescence titrations, and lifetime measurements confirm that the hydration environment of sub-stoichiometric Tb^3+^ remains unchanged.

Displacement experiments provide additional insight into the relative site preferences of the two lanthanides. We have previously shown that smaller Yb^3+^ cannot displace the optimally sized Tb^3+^ from a buried Asn_3_Asp_3_ site, and its addition does not lead to a decrease in Tb^3+^ emission.^18^ Whereas, when Tb^3+^-saturated LS2-1,5 is titrated with Yb^3+^, a partial decrease in Tb^3+^ emission is observed, approaching the signal expected for Tb_(1)_(LS2-1,5)_3_. These data indicate that Yb^3+^ preferentially displaces Tb^3+^ from site 2, which has a lower association constant and is less emissive, while Tb^3+^ remains bound at site 1, which has a higher association constant and is more emissive. This behaviour is consistent with the relative association constants extracted from global analysis of the luminescence titrations. Binding of Yb^3+^ in the lower association constant, size-promiscuous site 2 is an order of magnitude weaker than Tb^3+^ binding in the same site. This lower association constant explains both the requirement for a large excess of Yb^3+^ to replace Tb^3+^ and why 1 equivalent of added Yb^3+^ does not measurably affect Tb^3+^ binding.

Native MS further supports this model by revealing the formation of a heterobimetallic coiled coil complexes, YbTb(LS2-1,5)_3_, even under conditions where sufficient Tb^3+^ is present to exclusively form the TbTb(LS2-1,5)_3_ complex.

Together, these results support the formation of a well-defined heterobimetallic coiled coil, Yb_(2)_Tb_(1)_(LS2-1,5)_3_, in which the two lanthanide ions are spatially segregated and selectively bound at non-equivalent sites. We are keen to explore how this scaffold can enable selective binding and discrimination between different pairs of lanthanide ions. The N-terminal Asp_3_-based site 2 exhibits greater hydration and broader ion tolerance, while the C-terminal Asn_3_Asp_3_ site 1 is more coordinatively saturated and size-selective. This work thus demonstrates the first *de novo* designed peptide scaffold capable of simultaneously discriminating between and binding two different lanthanide ions at closely related yet functionally distinct sites.

Selective lanthanide discrimination remains a formidable challenge due to the minimal differences in ionic radii and coordination preferences across the series – an issue far more severe than for transition metals. The successful realization of Yb_(2)_Tb_(1)_(LS2-1,5)_3_ highlights how bottom-up *de novo* metalloprotein design can achieve a level of ion selectivity and positional control that begins to approach that evolved in biological lanthanide chemistry.

## Conclusions

We report the first example of a designed coiled coil scaffold capable of binding two distinct lanthanide ions at independent, non-equivalent sites, and present the first X-ray crystal structure of a bimetallic lanthanide coiled coil complex. Remarkably, the two Tb^3+^ ions are located at a shortest-to-date metal–metal separation of 11.9 Å in a coiled coil scaffold, with no shared ligands. This structure also provides the first direct crystallographic characterization of a lanthanide ion coordinated within an Asp_3_-only site, establishing a new benchmark in lanthanide-protein design.

The linear and modular arrangement of the binding sites allows precise control over intermetallic distances, enabling the development of molecular rulers with nanometre resolution (1–5 nm). Importantly, we demonstrate that selective binding and order of metal ion addition can be regulated through subtle differences in the coordination environments, modulated by ligand identity, site location, and solvent accessibility. Thereby highlighting the critical role of exogenous water ligands in lanthanide coordination chemistry.

Our dual-site designs produce a heterochromic coiled coil, in which the same metal ion binds two chemically distinct sites, resulting in different physical and spectroscopic properties. Furthermore, we achieve site-selective discrimination between two closely related lanthanide ions, Tb^3+^ and Yb^3+^, within a single scaffold. This represents a significant advance given the notorious difficulty of achieving such selectivity among lanthanides due to their similar ionic radii and coordination preferences.

Together, these findings establish new design principles for engineering selective, structurally well-defined lanthanide binding sites into *de novo* protein scaffolds. This work opens avenues for the creation of multifunctional metalloproteins and lays foundational groundwork for applications in sensing, catalysis, molecular magnetism, and rare-earth separation technologies.

## Author contributions

AFAP directed and initiated this study. LNS, AS, MJT, RIH, HELM, GMS,VB, SC, GR, NJBr and KAH performed the experiments. LNS, AS, MJT, RIH, HELM, GMS, JEL, VB, SC, ALL, NJBr, KAH, ACL, NJB and AFAP participated in the data analysis. AFAP wrote the manuscript.

## Conflicts of interest

The authors declare no conflicts of interest.

## Supplementary Material

SC-OLF-D6SC00813E-s001

SC-OLF-D6SC00813E-s002

## Data Availability

The EPR research data supporting this publication can be accessed at https://doi.org/10.17630/e8750808-4a63-41c9-bfa1-e32a8db40738. Supplementary information (SI): materials and methods, peptide characterization, and supporting experimental results. See DOI: https://doi.org/10.1039/d6sc00813e.

## References

[cit1] Cotruvo Jr. J. A., Featherston E. R., Mattocks J. A., Ho J. V., Laremore T. N. (2018). Lanmodulin: A Highly Selective Lanthanide-Binding Protein from a Lanthanide-Utilizing Bacterium. J. Am. Chem. Soc..

[cit2] Deblonde G. J. P., Mattocks J. A., Park D. M., Reed D. W., Cotruvo Jr. J. A., Jiao Y. (2020). Selective and Efficient Biomacromolecular Extraction of Rare-Earth Elements using Lanmodulin. Inorg. Chem..

[cit3] Dong Z., Mattocks J. A., Deblonde G. J. P., Hu D., Jiao Y., Cotruvo Jr. J. A., Park D. M. (2021). Bridging Hydrometallurgy and Biochemistry: A Protein-Based Process for Recovery and Separation of Rare Earth Elements. ACS Cent. Sci..

[cit4] Mattocks J. A., Jung J. J., Lin C.-Y., Dong Z., Yennawar N. H., Featherston E. R., Kang-Yun C. S., Hamilton T. A., Park D. M., Boal A. K., Cotruvo J. A. (2023). Enhanced rare-earth separation with a metal-sensitive lanmodulin dimer. Nature.

[cit5] Dong Z., Mattocks J. A., Seidel J. A., Cotruvo J. A., Park D. M. (2024). Protein-based approach for high-purity Sc, Y, and grouped lanthanide separation. Sep. Purif. Technol..

[cit6] Larrinaga W. B., Jung J. J., Lin C.-Y., Boal A. K., Cotruvo J. A. (2024). Modulating metal-centered dimerization of a lanthanide chaperone protein for separation of light lanthanides. Proc. Natl. Acad. Sci. U. S. A..

[cit7] Seidel J., Diep P., Dong Z., Cotruvo Jr. J. A., Park D. M. (2024). EF-Hand Battle Royale: Hetero-ion Complexation in Lanmodulin. JACS Au.

[cit8] Iranzo O., Cabello C., Pecoraro V. L. (2007). Heterochromia in Designed Metallopeptides: Geometry-Selective Binding of CdII in a De Novo Peptide. Angew. Chem., Int. Ed..

[cit9] Peacock A. F. A., Hemmingsen L., Pecoraro V. L. (2008). Using diastereopeptides to control metal ion coordination in proteins. Proc. Natl. Acad. Sci. U. S. A..

[cit10] Zastrow M. L., Peacock A. F. A., Stuckey J. A., Pecoraro V. L. (2012). Hydrolytic catalysis and structural stabilization in a designed metalloprotein. Nat. Chem..

[cit11] Teare P., Smith C. F., Adams S. J., Anbu S., Ciani B., Jeuken L. J. C., Peacock A. F. A. (2018). pH dependent binding in de novo hetero bimetallic coiled coils. Dalton Trans..

[cit12] Pirro F., Schmidt N., Lincoff J., Widel Z. X., Polizzi N. F., Liu L., Therien M. J., Grabe M., Chino M., Lombardi A., DeGrado W. F. (2020). Allosteric cooperation in a de novo-designed two-domain protein. Proc. Natl. Acad. Sci. U. S. A..

[cit13] Kashiwada A., Ishida K., Matsuda K. (2007). Lanthanide Ion-Induced Folding of De Novo Designed Coiled-Coil Polypeptides. Bull. Chem. Soc. Jpn..

[cit14] Berwick M. R., Lewis D. J., Jones A. W., Parslow R. A., Dafforn T. R., Cooper H. J., Wilkie J., Pikramenou Z., Britton M. M., Peacock A. F. A. (2014). De Novo Design of Ln(III) Coiled Coils for Imaging Applications. J. Am. Chem. Soc..

[cit15] Peacock A. F. A. (2025). Coiled coils as ligands for inclusion in the inorganic chemist's toolbox – For advances in MRI contrast agent design. J. Inorg. Biochem..

[cit16] Slope L. N., Hill M. G., Smith C. F., Teare P., de Cogan F. J., Britton M. M., Peacock A. F. A. (2020). Tuning coordination chemistry through the second sphere in designed metallocoiled coils. Chem. Commun..

[cit17] Berwick M. R., Slope L. N., Smith C. F., King S. M., Newton S. L., Gillis R. B., Adams G. G., Rowe A. J., Harding S. E., Britton M. M., Peacock A. F. A. (2016). Location dependent coordination chemistry and MRI relaxivity, in de novo designed lanthanide coiled coils. Chem. Sci..

[cit18] Slope L. N., Daubney O. J., Campbell H., White S. A., Peacock A. F. A. (2021). Location-Dependent Lanthanide Selectivity Engineered into Structurally Characterized Designed Coiled Coils. Angew. Chem., Int. Ed..

[cit19] Tochtrop G. P., Richter K., Tang C., Toner J. J., Covey D. F., Cistola D. P. (2002). Energetics by NMR: Site-specific binding in a positively cooperative system. Proc. Natl. Acad. Sci. U. S. A..

[cit20] Beeby A., Clarkson, I.; S M., Dickins R., Faulkner S., Parker D., Royle L., S. de Sousa A., A. Gareth Williams J., Woods M. (1999). Non-radiative deactivation of the excited states of europium, terbium and ytterbium complexes by proximate energy-matched OH, NH and CH oscillators: an improved luminescence method for establishing solution hydration states. J. Chem. Soc., Perkin Trans. 2.

[cit21] Martin R. E., Pannier M., Diederich F., Gramlich V., Hubrich M., Spiess H. W. (1998). Determination of end-to-end distances in a series of TEMPO diradicals of up to 2.8 nm length with a new four-pulse double electron electron resonance experiment. Angew. Chem., Int. Ed..

[cit22] Milov A., Salikhov K., Shirov M. (1981). Application of ELDOR in electron-spin echo for paramagnetic center space distribution in solids. Fiz. Tverd. Tela.

[cit23] Schiemann O., Heubach C. A., Abdullin D., Ackermann K., Azarkh M., Bagryanskaya E. G., Drescher M., Endeward B., Freed J. H., Galazzo L., Goldfarb D., Hett T., Esteban Hofer L., Fábregas Ibáñez L., Hustedt E. J., Kucher S., Kuprov I., Lovett J. E., Meyer A., Ruthstein S., Saxena S., Stoll S., Timmel C. R., Di Valentin M., McHaourab H. S., Prisner T. F., Bode B. E., Bordignon E., Bennati M., Jeschke G. (2021). Benchmark Test and Guidelines for DEER/PELDOR Experiments on Nitroxide-Labeled Biomolecules. J. Am. Chem. Soc..

[cit24] Giannoulis A. ; Ben-Ishay Y.; Goldfarb D., Chapter Eight - Characteristics of Gd(III) spin labels for the study of protein conformations, in Methods in Enzymology, ed J. A. Cotruvo, Academic Press, 2021, Vol. 651, pp 235–290 10.1016/bs.mie.2021.01.040 33888206

[cit25] El Mkami H., Hunter R. I., Cruickshank P. A. S., Taylor M. J., Lovett J. E., Feintuch A., Qi M., Godt A., Smith G. M. (2020). High-sensitivity Gd3+–Gd3+ EPR distance measurements that eliminate artefacts seen at short distances. Magn. Reson..

[cit26] Cruickshank P. A. S., Bolton D. R., Robertson D. A., Hunter R. I., Wylde R. J., Smith G. M. (2009). A kilowatt pulsed 94 GHz electron paramagnetic resonance spectrometer with high concentration sensitivity, high instantaneous bandwidth, and low dead time. Rev. Sci. Instrum..

[cit27] Fábregas Ibáñez L., Jeschke G., Stoll S. (2020). DeerLab: a comprehensive software package for analyzing dipolar electron paramagnetic resonance spectroscopy data. Magn. Reson..

[cit28] Lovejoy B., Choe S., Cascio D., McRorie D., DeGrado W., Eisenberg D. (1993). Crystal structure of a synthetic triple-stranded alpha-helical bundle. Science.

[cit29] Ruckthong L., Zastrow M. L., Stuckey J. A., Pecoraro V. L. (2016). A Crystallographic Examination of Predisposition versus Preorganization in de Novo Designed Metalloproteins. J. Am. Chem. Soc..

[cit30] Wang Z.-X. (1995). An exact mathematical expression for describing competitive binding of two different ligands to a protein molecule. FEBS Lett..

[cit31] Velázquez Campoy A., Freire E. (2005). ITC in the post-genomic era Priceless. Biophys. Chem..

[cit32] De Crescenzo G., Litowski J. R., Hodges R. S., O'Connor-McCourt M. D. (2003). Real-Time Monitoring of the Interactions of Two-Stranded de Novo Designed Coiled-Coils: Effect of Chain Length on the Kinetic and Thermodynamic Constants of Binding. Biochemistry.

[cit33] Ghosh D., Lee K.-H., Demeler B., Pecoraro V. L. (2005). Linear Free-Energy Analysis of Mercury(II) and Cadmium(II) Binding to Three-Stranded Coiled Coils. Biochemistry.

[cit34] Banham J. E., Baker C. M., Ceola S., Day I. J., Grant G. H., Groenen E. J. J., Rodgers C. T., Jeschke G., Timmel C. R. (2008). Distance measurements in the borderline region of applicability of CW EPR and DEER: A model study on a homologous series of spin-labelled peptides. J. Magn. Reson..

[cit35] DeGrado W. F., Di Costanzo L., Geremia S., Lombardi A., Pavone V., Randaccio L. (2003). Sliding Helix and Change of Coordination Geometry in a Model Di-MnII Protein. Angew. Chem., Int. Ed..

[cit36] Lombardi A., Summa C. M., Geremia S., Randaccio L., Pavone V., DeGrado W. F. (2000). Retrostructural analysis of metalloproteins: Application to the design of a minimal model for diiron proteins. Proc. Natl. Acad. Sci. U. S. A..

[cit37] Geremia S., Di Costanzo L., Randaccio L., Engel D. E., Lombardi A., Nastri F., DeGrado W. F. (2005). Response of a Designed Metalloprotein to Changes in Metal Ion Coordination, Exogenous Ligands, and Active Site Volume Determined by X-ray Crystallography. J. Am. Chem. Soc..

[cit38] Zaytsev D. V., Morozov V. A., Fan J., Zhu X., Mukherjee M., Ni S., Kennedy M. A., Ogawa M. Y. (2013). Metal-binding properties and structural characterization of a self-assembled coiled coil: Formation of a polynuclear Cd–thiolate cluster. J. Inorg. Biochem..

[cit39] Zhang S.-Q., Chino M., Liu L., Tang Y., Hu X., DeGrado W. F., Lombardi A. (2018). De Novo Design of Tetranuclear Transition Metal Clusters Stabilized by Hydrogen-Bonded Networks in Helical Bundles. J. Am. Chem. Soc..

[cit40] Boyle A. L., Rabe M., Crone N. S. A., Rhys G. G., Soler N., Voskamp P., Pannu N. S., Kros A. (2019). Selective coordination of three transition metal ions within a coiled-coil peptide scaffold. Chem. Sci..

[cit41] Falcone E., Mathieu E., Hureau C. (2025). Lanthanide(iii)-binding peptides and proteins: coordination properties and applications. Chem. Soc. Rev..

[cit42] Caldwell S. J., Haydon I. C., Piperidou N., Huang P.-S., Bick M. J., Sjöström H. S., Hilvert D., Baker D., Zeymer C. (2020). Tight and specific lanthanide binding in a de novo TIM barrel with a large internal cavity designed by symmetric domain fusion. Proc. Natl. Acad. Sci. U. S. A..

[cit43] Featherston E. R., Issertell E. J., Cotruvo Jr. J. A. (2021). Probing Lanmodulin's Lanthanide Recognition via Sensitized Luminescence Yields a Platform for Quantification of Terbium in Acid Mine Drainage. J. Am. Chem. Soc..

[cit44] Lombardi A., Pirro F., Maglio O., Chino M., DeGrado W. F. (2019). De Novo Design of Four-Helix Bundle Metalloproteins: One Scaffold, Diverse Reactivities. Acc. Chem. Res..

[cit45] Joh N. H., Wang T., Bhate M. P., Acharya R., Wu Y., Grabe M., Hong M., Grigoryan G., DeGrado W. F. (2014). De novo design of a transmembrane Zn^2+^-transporting four-helix bundle. Science.

