# Supplementary material for: Design of a site selective heterochromic bimetallic lanthanide coiled coil with nanometre-scale control
Source: Chem Sci. 2026 May 12;17(25):12313–23. doi: 10.1039/d6sc00813e (PMC13185964; doi:10.1039/d6sc00813e)
Supplement: SC-017-D6SC00813E-s002 [file SC-017-D6SC00813E-s002.pdf]

Supplementary Information (SI) for Chemical Science.
This journal is © The Royal Society of Chemistry 2026

Supporting Information

Design of a site selective heterochromic bimetallic lanthanide coiled coil with nanometre-scale control

Louise N. Slope,[a] Anokhi Shah,[b,c] Michael J. Taylor,[b,c] Valentina Borghesani,[d] Simon G. Caulton,[e] Nikolas J. Brooks,[e] Kate A. Hadley,[a] Georgina Rose,[a] Robert I. Hunter,[b] Hassane EL Mkami,[b] Graham M. Smith,[b] Aneika C. Leney,[e] Niklaas J. Buurma,[f] Andrew L. Lovering,[e] Janet E. Lovett[b,c] and Anna F. A. Peacock[a]*

[a] School of Chemistry, University of Birmingham, Edgbaston, B15 2TT, UK

[b] SUPA School of Physics and Astronomy, University of St Andrews, KY16 9SS, UK

[c] BSRC, University of St Andrews, KY16 9ST, UK

[d] Department of Chemistry, Life Sciences, and Environmental Sustainability, University of Parma, Parco Area delle Scienze 17A, 43124, Parma, Italy

[e] School of Biosciences, University of Birmingham, Edgbaston, B15 2TT, UK

[f] Physical Organic Chemistry Centre, School of Chemistry, Cardiff University, Main Building, Park Place, Cardiff, CF10 3AT, Cymru/Wales, UK

* a.f.a.peacock@bham.ac.uk

Contents:

1. Materials and Methods
2. Figure S1 – HPLC and mass spectra for LS2-1,2
3. Figure S2 – HPLC and mass spectra for LS2-1,3
4. Figure S3 – HPLC and mass spectra for LS2-1,4
5. Figure S4 – HPLC and mass spectra for LS2-1,5
6. Figure S5 – HPLC and mass spectra for LS3-1,6
7. Figure S6 – HPLC and mass spectra for LS2'-2,3
8. Figure S7 – HPLC and mass spectra for LS2-1,5(7Q)
9. Figure S8 – HPLC and mass spectra for LS2-1,5(35Q)
10. Figure S9 – Tb^{3+} titrations monitored by CD
11. Table S1 – Summary of % folding
12. Figure S10 – Tb^{3+} titrations monitored by luminescence
13. Figure S11 – Models of control peptides
14. Figure S12 – Tb^{3+} CD titrations of control peptides
15. Figure S13 – Luminescence titrations of control peptides
16. Figures S14-S16 – Global fit analysis with different cooperative factors
17. Table S2 – Fitting parameters for binding model
18. Figure S17 – Cooperativity factors
19. Figure S18 – Speciation simulation
20. Figure S19 – Luminescent lifetime decay profiles
21. Figure S20 – Luminescent lifetime decay profiles for control peptides
22. Table S3 – Number of water molecules coordinated to peptide bound Tb^{3+}
23. Figure S21 – Gd^{3+} titrations monitored by CD
24. Figure S22 – ED-FS spectra at W-band
25. Figure S23 – Echo decay curves

26. Figure S24 – ED-FS showing excitation bandwidth and frequency of the pump pulse in the DEER experiments.
27. Table S4 – LS2-1,3 W-band DEER parameters
28. Table S5 – LS2-1,4 W-band DEER parameters
29. Table S6 – LS2-1,5 W-band DEER parameters
30. Table S7 – LS3-1,6 DEER parameters at W- and Q-band
31. Table S8 – DeerLab results
32. Figure S25 – DEER results from DeerLab using Gaussian fitting
33. Figure S26 - DEER results from DeerAnalysis using Tikhonov regularization
34. Figure S27 - LS2-1,3 DEER with distances extracted using Tikhonov regularization
35. Figure S28 - LS2-1,4 DEER with distances extracted using Tikhonov regularization
36. Figure S29 – LS3-1,6 DEER with distances extracted using Tikhonov regularization
37. Table S9 – X-ray data collection and refinement statistics
38. Figure S30 – Close up view of Tb^{3+} sites with electron density maps
39. Figure S31 – External Tb^{3+} sites that facilitate crystal packing
40. Figure S32 – CD LS2-1,5 titrations with Tb^{3+}/Yb^{3+}
41. Figure S33 – Luminescence Tb^{3+} displacement
42. Figure S34 – Apo native MS
43. Figure S35 – Native MS binding studies
44. Table S10 – Theoretical and observed native MS molecular weights
45. Figure S36 – Structural overlay of Asn_3Asp_3 Tb^{3+}-binding site
46. References

Materials and methods, 36 supplementary figures and ten supplementary tables.

1. Materials and Methods:

Materials: Chemicals were used as received and purchased from; Sigma Aldrich (diethyl ether, gadolinium chloride hexahydrate, ytterbium chloride hexahydrate and terbium chloride hexahydrate), Pepceuticals (Fmoc protected amino acids, HBTU (O-benzotriazole-N,N,N',N'-tetramethyluronium-hexafluorophosphate), synthesis grade DMF (dimethylformamide) and 20% piperidine in DMF premix), AGTC Bioproducts Ltd. (rink amide MBHA resin, NMP (N-methyl-2-pyrrolidone), DIEA (N,N-diisopropylethylamine) and DCM (dichloromethane)), Acros Organics (xylenol orange sodium salt, acetic anhydride, TIPS (triisopropylsilane) and TFA (trifluoroacetic acid)), Rathburn Chemicals Ltd. (>99.9% DMF) and Fisher Scientific Ltd. (HPLC (high pressure liquid chromatography) grade water and acetonitrile, HEPES (2-[4-(2-hydroxyethyl)piperazin-1-yl]ethanesulfonic acid), urea, EDTA (ethylenediaminetetraacetic acid) and glacial acetic acid).

Peptide Synthesis, Characterisation and Purification: Peptides were synthesized on a CEM Liberty Blue automated peptide synthesiser on rink amide MBHA resin (0.25 mmol scale, 0.68 mmol g^{-1}), using standard Fmoc amino acid solid-phase peptide synthesis protocols[1] and purified and characterised as previously reported.[2, 3] HBTU, like other uronium-based coupling agents, can cause airborne skin sensitization and may even trigger anaphylactic reactions; therefore, the activating mixture should be handled with caution.[4, 5]

UV-visible Spectroscopy: UV-visible spectra were recorded on a Shimadzu 1800 UV Spectrophotometer, recorded in single beam mode with a medium scan speed, slit width of 1.0 nm, data interval of 1.0 nm and scan range 420-260 nm, using a 1 cm pathlength, 700 μL quartz cuvette.

Solution Preparation: The concentration of freshly prepared peptide stock solutions in deionized water, were deduced from the absorption of the Trp residues at 280 nm (ε_{280} = 5690 M^{-1} cm^{-1}) in 7 M aqueous urea, performed in triplicate for accuracy and left for 10 mins prior to quantitative UV determination. Note – most peptides contain two Trp residues with the exception of the control peptides. The concentrations of freshly prepared stock solutions of $LnCl_3$ (~1 mM) in deionized water, were determined in triplicate using a xylenol orange indicator and EDTA titration with Ln^{3+} standard solutions, following a procedure previously reported by Fedeli and co-workers.[6]

Circular Dichroism: CD spectra were recorded on a Jasco J-715 Spectropolarimeter in a 1 mm pathlength quartz cuvette. The optical chamber was purged with nitrogen and kept under a nitrogen atmosphere throughout the duration of the experiments. Aliquots of 1 mM stock solutions of $LnCl_3$ (where Ln^{3+} is either Tb^{3+} or Yb^{3+}) were titrated into 30 μM peptide monomer in 10 mM HEPES buffer pH 7.0, as previously reported.[7] All solutions were left to equilibrate for 10 minutes before recording spectra, and the observed ellipticity converted into molar ellipticity, with the helical content calculated as the percentage folded, based on the theoretical maximum ellipticity as reported by Scholtz *et al.*.[8]

Luminescence: Luminescence data for Tb^{3+} emission signals were acquired on an Edinburgh Instruments Fluorescence FL920 system with a 450 W Xenon arc lamp and a Hamamatsu R928 photomultiplier tube. The emission monochromator was fitted with two interchangeable gratings blazed at 500 nm and 1200 nm and the data was collected using F900 spectrometer analysis software. A 455 nm long pass filter was used, the solutions were excited at 280 nm and the emission scanned in the range 455-700 nm using an excitation slit width of 5 nm and an emission slit width of 5 nm with a 0.5 sec dwell time, following a previously reported procedure.[7] All emission spectra were recorded in a 1 cm pathlength, 700 μL quartz cuvette. Aliquots of 1 mM stock solutions of $TbCl_3$ were titrated into 30 μM peptide monomer in 10 mM HEPES buffer pH 7.0, as previously reported,[7] and the emission profile recorded after 10 minutes equilibration. The

signal (integrated from 530-560 nm and corrected for Trp contribution) was measured in triplicate and a standard deviation error reported. Titrations of $TbCl_3$ into 30 μM monomer solutions were also carried out in the presence of 1 equivalent of $YbCl_3$ (10 μM). Errors reported correspond to the standard deviation of the three repeats.

Tb^{3+} lifetimes in D_2O and H_2O were determined for the $Tb(Pep)_3$ complexes. Solutions containing 0.3 equivalents Tb^{3+} (10 μM) and 100 μM peptide monomer, in 10 mM HEPES buffer pH 7.0, were monitored using a μF Flashlamp light source (50 Hz) on the Edinburgh Instruments spectrofluorimeter, collecting over a 10 ms (H_2O) or 20 ms (D_2O) time range, with a lamp trigger delay of 0.1 ms. LS2-1,5 was also monitored in the presence of 1 equivalent Yb^{3+} (30 μM) and 0.3 equivalents Tb^{3+} (10 μM). Data was fitted to both mono- and bi- exponential decay kinetics in the F900 software using the tailfit model, described by the mathematical expression, $R(t) = B_1 e^{(-t/\tau_1)} + B_2 e^{(-t/\tau_2)}$. The sample decay model is a function of the variable time, *t*, and kinetic parameters. B is the pre-exponential factor made up of both instrumental and sample parameters and τ is the characteristic lifetime, measuring the time it takes for the florescence to decay to level 1/e (*ca.* 37% its original value). From the observed lifetime the number of coordinated water molecules was determined using the Parker-Beeby equation.[9]

Data Analysis: Luminescence titration data were analysed using Originlab Origin, using a custom-developed function definition file to code the binding model in Origin-C.

Derivation of the 2-binding-sites equilibrium binding model

The binding model involves a coiled coil, offering two different bindings sites, viz. site 1 and site 2, which can be occupied by Tb with binding affinities K_1 and K_2 and the potential for (anti)cooperativity of the interactions as quantified by a cooperativity factor *c* (Scheme S1). To

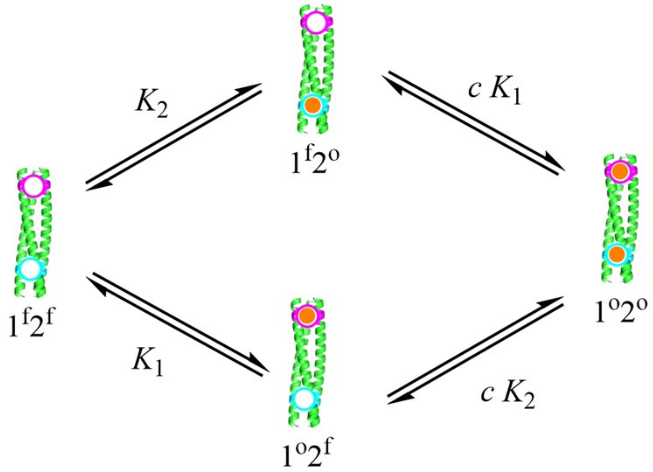

allow mathematical development of this binding model, each binding site is restricted to an exact 1:1 stoichiometry.

Scheme S1

Let $[1^f2^f]$ be the concentration of coiled coil with both binding sites free, $[1^o2^f]$ the concentration of coiled coil with binding site 1 occupied and binding site 2 free, $[1^f2^o]$ the concentration of coiled coil with binding site 1 free and binding site 2 occupied, and $[1^o2^o]$ the concentration of coiled coil with both binding site 1 and binding site 2 occupied.

There may be small fractions of potential impurities and/or small errors in the calculation of concentrations in the samples of the coiled coils and in the terbium samples. To allow for these during the fitting, the total actual concentration of coiled coil, $[\text{coil}]_{\text{tot,actual}}$, is defined as the product of a purity factor (*p_fac*) and the expected (calculated) total concentration of coiled coil, $[\text{coil}]_{\text{tot,calc}}$, which will be a non-optimisable parameter in the data analysis model (Eqn 1.)

$[\text{coil}]_{\text{tot,actual}} = p\_fac \times [\text{coil}]_{\text{tot,calc}}$	Eqn. 1

This purity factor also indirectly corrects for any small discrepancies in the concentration of the solutions of Tb. This is because the concentrations of coiled coil will be adjusted so that the binding stoichiometries are 1:1 as required by the model. For good samples, *p_fac* is expected to be close to 1.

Because different coiled coils are used in these experiments, there is a separate purity factor for each coiled coil in the global data analysis.

The mass-balance equation for the coiled coil is thus defined by Eqn. 2.

$p\_fac \times [coil]_{tot,calc} = [coil]_{tot,actual} = [1^f2^f] + [1^o2^f] + [1^f2^o] + [1^o2^o]$	Eqn. 2

$\therefore p\_fac \times [coil]_{tot,calc} = [1^f2^f] + K_1 \times [1^f2^f] \times [Tb]_f + K_2 \times [1^f2^f] \times [Tb]_f +$ $c \times K_1 \times K_2 \times [1^f2^f] \times [Tb]_f^2$	Eqn. 3

Where $[Tb]_f$ is the concentration of free terbium.

$\therefore p\_fac \times [coil]_{tot,calc} = \{1 + K_1 \times [Tb]_f + K_2 \times [Tb]_f + c \times K_1 \times K_2 \times [Tb]_f^2\} \times [1^f2^f]$	Eqn. 4

$\therefore [1^f2^f] = p\_fac \times [coil]_{tot,calc} / \{1 + K_1 \times [Tb]_f + K_2 \times [Tb]_f + c \times K_1 \times K_2 \times [Tb]_f^2\}$	Eqn. 5

Similarly, the mass-balance equation for terbium is given by Eqn. 6

$[Tb]_{tot} = [Tb]_f + K_1 \times [1^f2^f] \times [Tb]_f + K_2 \times [1^f2^f] \times [Tb]_f + 2 \times c \times K_1 \times K_2 \times [1^f2^f] \times [Tb]_f^2$	Eqn. 6

Inserting Eqn. 5 into Eqn. 6, followed by multiplication of both sides of the equation by the divisor of Eqn. 5 gives Eqn. 7.

$[Tb]_{tot} \times \{1 + K_1 \times [Tb]_f + K_2 \times [Tb]_f + c \times K_1 \times K_2 \times [Tb]_f^2\}$ $=$	Eqn. 7

$[\mathrm{Tb}]_\mathrm{f} \times \{1 + K_1 \times [\mathrm{Tb}]_\mathrm{f} + K_2 \times [\mathrm{Tb}]_\mathrm{f} + c \times K_1 \times K_2 \times [\mathrm{Tb}]_\mathrm{f}^2\}$ $+ K_1 \times \mathrm{p\_fac} \times [\mathrm{coil}]_{\mathrm{tot,calc}} \times [\mathrm{Tb}]_\mathrm{f} + K_2 \times \mathrm{p\_fac} \times [\mathrm{coil}]_{\mathrm{tot,calc}} \times [\mathrm{Tb}]_\mathrm{f}$ $+ 2 \times c \times K_1 \times K_2 \times \mathrm{p\_fac} \times [\mathrm{coil}]_{\mathrm{tot,calc}} \times [\mathrm{Tb}]_\mathrm{f}^2$	

Writing out the multiplications and colour coding by power of $[\mathrm{Tb}]_\mathrm{f}$ gives Eqn. 8.

$\therefore [\mathrm{Tb}]_{\mathrm{tot}} + (K_1 + K_2) \times [\mathrm{Tb}]_{\mathrm{tot}} \times [\mathrm{Tb}]_\mathrm{f} + c \times K_1 \times K_2 \times [\mathrm{Tb}]_{\mathrm{tot}} \times [\mathrm{Tb}]_\mathrm{f}^2$ $=$ $[\mathrm{Tb}]_\mathrm{f} + (K_1 + K_2) \times [\mathrm{Tb}]_\mathrm{f}^2 + c \times K_1 \times K_2 \times [\mathrm{Tb}]_\mathrm{f}^3 + K_1 \times \mathrm{p\_fac} \times [\mathrm{coil}]_{\mathrm{tot,calc}} \times [\mathrm{Tb}]_\mathrm{f} + K_2 \times \mathrm{p\_fac} \times [\mathrm{coil}]_{\mathrm{tot,calc}} \times [\mathrm{Tb}]_\mathrm{f} + 2 \times c \times K_1 \times K_2 \times \mathrm{p\_fac} \times [\mathrm{coil}]_{\mathrm{tot,calc}} \times [\mathrm{Tb}]_\mathrm{f}^2$	Eqn. 8

Collecting the colour-coded terms of equal power gives Eqn. 9.

$0 = c \times K_1 \times K_2 \times [\mathrm{Tb}]_\mathrm{f}^3 + (K_1 + K_2) \times [\mathrm{Tb}]_\mathrm{f}^2 + 2 \times c \times K_1 \times K_2 \times \mathrm{p\_fac} \times [\mathrm{coil}]_{\mathrm{tot,calc}} \times [\mathrm{Tb}]_\mathrm{f}^2 - c \times K_1 \times K_2 \times [\mathrm{Tb}]_{\mathrm{tot}} \times [\mathrm{Tb}]_\mathrm{f}^2 + [\mathrm{Tb}]_\mathrm{f} + K_1 \times \mathrm{p\_fac} \times [\mathrm{coil}]_{\mathrm{tot,calc}} \times [\mathrm{Tb}]_\mathrm{f} + K_2 \times \mathrm{p\_fac} \times [\mathrm{coil}]_{\mathrm{tot,calc}} \times [\mathrm{Tb}]_\mathrm{f} - (K_1 + K_2) \times [\mathrm{Tb}]_{\mathrm{tot}} \times [\mathrm{Tb}]_\mathrm{f} - [\mathrm{Tb}]_{\mathrm{tot}}$	Eqn. 9

$\therefore 0 = c \times K_1 \times K_2 \times [\mathrm{Tb}]_\mathrm{f}^3 + \{(K_1 + K_2) + 2 \times c \times K_1 \times K_2 \times \mathrm{p\_fac} \times [\mathrm{coil}]_{\mathrm{tot,calc}} - c \times K_1 \times K_2 \times [\mathrm{Tb}]_{\mathrm{tot}}\} \times [\mathrm{Tb}]_\mathrm{f}^2 + \{1 + K_1 \times \mathrm{p\_fac} \times [\mathrm{coil}]_{\mathrm{tot,calc}} + K_2 \times \mathrm{p\_fac} \times [\mathrm{coil}]_{\mathrm{tot,calc}} - (K_1 + K_2) \times [\mathrm{Tb}]_{\mathrm{tot}}\} \times [\mathrm{Tb}]_\mathrm{f} - [\mathrm{Tb}]_{\mathrm{tot}}$	Eqn. 10

Equation 10 turns into the equation presented by Tochtrop *et al.*[10] for $\mathrm{p\_fac} \times [\mathrm{coil}]_{\mathrm{tot,calc}} = M_\mathrm{t}$ and $[\mathrm{Tb}]_\mathrm{f} = \mathrm{L}$.

Eqn. 10 is a cubic equation of the form $ax^3 + bx^2 + cx + d = 0$, with

$$a = c \times K_1 \times K_2$$

$$b = c \times K_1 \times K_2 \times (2\times[\text{coil}]_{\text{tot,actual}} - [\text{Tb}]_{\text{tot}}) + (K_1+K_2)$$

$$c = 1 + K_1 \times [\text{coil}]_{\text{tot,actual}} + K_2 \times [\text{coil}]_{\text{tot,actual}} - (K_1+K_2) \times [\text{Tb}]_{\text{tot}}$$

$$d = -1 \times [\text{Tb}]_{\text{tot}}$$

Analytical solutions exist for cubic equations, see http://www.1728.org/cubic2.htm, and these can be developed through the following steps.

$$f = ((3*c/a)-(b^2/a^2))/3$$

$$g = ((2*(b^3)/(a^3))-(9*b*c/(a^2))+(27*d/a))/27$$

$$h = ((g^2)/4)+((f^3)/27)$$

$$i = \text{sqrt}((g^2/4)-h)$$

$$j = i^{(1/3)}$$

$$k = \text{acos}(-1*(g / (2*i)))$$

$$L = -1*j$$

$$M = \cos(k/3)$$

$$N = \text{sqrt}(3)*\sin(k/3)$$

$$P = -1*(b/(3*a))$$

The three solutions of the cubic equation are then:

$$x_1 = 2j \bullet \cos(k/3)-(b/3a)$$

$$x_2 = L \bullet (M + N) + P$$

$$x_3 = L \bullet (M - N) + P$$

The only physically meaningful root of the cubic equation corresponds to the free terbium concentration, $[Tb]_f$, which must lie in the range of 0 M – $[Tb]_{tot}$. From simulations with reasonable numbers, only one of the three solutions is reasonable and this always seems to be the same solution, given by equation 11.

$[Tb]_f = 2 \times j \times \cos(k/3) - (b/(3\times a))$	Eqn. 11

The concentrations of the various species can then be calculated using equations 5 and 12-14

$[1^o2^f] = K_1 \times [12] \times [Tb]_f$	Eqn. 12

$[1^f2^o] = K_2 \times [12] \times [Tb]_f$	Eqn. 13

$[1^o2^o] = c \times K_1 \times K_2 \times [12] \times [Tb]_f^2$	Eqn. 14

We next define two molar response factors which link observed luminescence intensity to the concentration of Tb^{3+} bound in each of the two binding sites. The response factors themselves represent a combination of luminescence quantum yield for each site and instrument settings such as slit widths and detector settings. We assume that both sites are fully independent and hence the response factors are constant.

The observed luminescence signal is then given by equation 15 for the coiled coil where the tryptophan sensitiser is adjacent to binding site 1, by equation 16 for the coiled coil where the tryptophan sensitiser is adjacent to binding site 2, and by equation 17 for the coiled coil where tryptophan sensitisers are adjacent to binding sites 1 and 2.

signal = background1 + response_factor1 × ($[1^o2^f] + [1^o2^o]$)	Eqn. 15

signal = background2 + response_factor2 × ($[1^f2^o] + [1^o2^o]$)	Eqn. 16

signal = background3 + response_factor1 × ($[1^o2^f] + [1^o2^o]$) + response_factor2 × ($[1^f2^o] + [1^o2^o]$)	Eqn. 17

The signal itself also depends on the settings of the fluorimeter. For example, for different settings, the observed luminescence signal for the coiled coil where the tryptophan sensitiser is adjacent to binding site 1 is given by equation 18.

signal = background1 + SettingsCorrection (response_factor1 × ($[1^o2^f] + [1^o2^o]$))	Eqn. 18

Equation 18 considers that the observed intensity as a result of binding should scale by a constant factor, whereas the background signal is not necessarily affected in precisely the same way.

If different settings (e.g. slit widths) have been used, these are identified by an integer in the dataset, viz. 1 or 2, identifying the settings for the experiment (in this model only two different settings are supported). If the column holds a 2, then the SettingsCorrection multiplier is used.

Implementation of the binding model

Data format

The titration data is organised in Origin as illustrated in Table 1.

Table 1: Data organisation.

[Tb]	[coiled coil]	site	settings	signal
.	.	.	.	.
.	.	.	.	.
.	.	.	.	.

The column "[Tb]" holds the concentration of Tb^{3+} for each data point. The column "[coiled coil]" holds the concentration of coiled coils, i.e. 1/3 of the concentration of the monomers. The column "site" identifies which of the two binding sites contribute to the observed signal with 1 corresponding to 7Q-35W, 2 corresponding to 7W-35Q, and 3 corresponding to 7W-35W. The column "settings" allows the identification of experiments which have been carried out with the same settings on the fluorimeter. This column holds a 1 or a 2 to identify a maximum of two different settings of the fluorimeter used for the titrations. For experiments where "settings" is set to 2, relative response factors should be identical but these are multiplied by SettingsCorrection to correct for different experimental settings. This correction is not needed for the backgrounds because these may be different already anyway.

Data provided in this format allows us to globally analyse all titrations, using the minimum number of optimizable parameters.

Origin-C code – naming of parameters

Equations 1-19 were translated into Origin-C code, which is available as an Origin function definition file (fdf) from from https://github.com/niekbuurmah2o/equilibrium-models-for-bimetallic-lanthanide-coiled-coil, using the following translation table for the various parameters.

$[Tb]_{tot}$	`binder`
$[Tb]_{f}$	`binderfree`
$[coil]_{tot,calc}$	`host`
p_fac (one for each coiled coil)	`purityfactor1, purityfactor2, purityfactor3`
$[coil]_{tot,actual}$	`actualhost`
$[1^f2^f]$	`hostfree`
$[1^o2^f]$	`siteoneoccupied_sitetwofree`
$[1^f2^o]$	`siteonefree_sitetwooccupied`
$[1^o2^o]$	`bothsitesoccupied`
c	`cooperativity`
K_1	`K1`
K_2	`K2`
signal	`signal`
background1, background2, background3 for settings 1 and 2	`background11, background12, background13, background21, background22, background23,`
response_factor1, response_factor2	`response1, response2`
SettingsCorrection	`settingscorrectionfactor`

Origin-C code

The translation table above results in the following Origin-C code.

```
double actualhost, hostfree, binderfree, siteoneoccupied_sitetwofree,

siteonefree_sitetwooccupied, bothsitesoccupied;

double a,b,c,d;

double f,g,h;

double i,j,k;

double L,M,N,P;

int int_settings;

int int_site;


// Make the setting and site numbers integers

int_settings = nint(settings);

int_site = nint(site);


// The following lines identify the coiled coil by which one of its binding sites gives rise to

fluorescence.

switch( int_site )

{

case 1:

        actualhost = purityfactor1*host;

        break;


case 2:

        actualhost = purityfactor2*host;
```

```
        break;

case 3:
        actualhost = purityfactor3*host;
        break;
}

//Define a, b, c and d
a = cooperativity*K1*K2;
b = cooperativity*K1*K2*(2*actualhost-binder)+(K1+K2);
c = 1 + K1*actualhost + K2*actualhost - (K1+K2)*binder;
d = -1*binder;

// see http://www.1728.org/cubic2.htm for solution to cubic

f = ((3*c/a)-(b^2/a^2))/3;
g = ((2*(b^3)/(a^3))-(9*b*c/(a^2))+(27*d/a))/27;
h = ((g^2)/4)+((f^3)/27);

i = sqrt((g^2/4)-h);
j = i^(1/3);
k = acos(-1*(g / (2*i)));

L = -1*j;
M = cos(k/3);
N = sqrt(3)*sin(k/3);
P = -1*(b/(3*a));
```

```
// From simulations with reasonable numbers, only one of the three solutions is the reasonable
one and this always seems to be the same one

binderfree = 2*j*cos(k/3)-(b/(3*a));
hostfree = actualhost / (1 + (K1+K2)*binderfree + cooperativity*K1*K2*(binderfree^2));
siteoneoccupied_sitetwofree = K1*hostfree*binderfree;
siteonefree_sitetwooccupied = K2*hostfree*binderfree;
bothsitesoccupied = cooperativity*K1*K2*hostfree*(binderfree^2);

// calculated signal depends on settings (int_settings) and on binding site (int_site)

switch( int_settings )
{
// For settings 1:
case 1:

        switch( int_site )
        {
        case 1:
                signal = (background11 + response1*(siteoneoccupied_sitetwofree +
bothsitesoccupied));
        break;

        case 2:
                signal = (background12 + response2*(siteonefree_sitetwooccupied +
bothsitesoccupied));
```

```
        break;

        case 3:
                signal = (background13 + response1*(siteoneoccupied_sitetwofree +
bothsitesoccupied) + response2*(siteonefree_sitetwooccupied + bothsitesoccupied));
        break;
        }

break;

// For settings 2:
case 2:

        switch( int_site )
        {
        case 1:
                signal = (background21 +
settingscorrectionfactor*response1*(siteoneoccupied_sitetwofree + bothsitesoccupied));
        break;

        case 2:
                signal = (background22 +
settingscorrectionfactor*response2*(siteonefree_sitetwooccupied + bothsitesoccupied));
        break;

        case 3:
```

```
            signal = (background23 +
settingscorrectionfactor*(response1*(siteoneoccupied_sitetwofree + bothsitesoccupied) +
response2*(siteonefree_sitetwooccupied + bothsitesoccupied)));
        break;
        }
}
```

Competitive binding model

The displacement titration data were analysed using a competitive binding model, assuming that site 1 is fully occupied with Tb^{3+} and that no displacement takes place in site 1. Displacement therefore only takes place in site 2 and this is the only site taken into account in displacement data analysis. In line with this assumption, a total concentration of Tb^{3+} of 10 μM was used during fitting because the other 10 μM of Tb^{3+} are tightly bound in site 1.

The competitive binding model results in a cubic equation, the physically reasonable solution of which was derived by Wang in terms of dissociation constants.<< https://febs.onlinelibrary.wiley.com/doi/10.1016/0014-5793%2895%2900062-E >> We have used this cubic equation in the notation of Campoy and Freire involving association constants, viz. Equation 22 in Ref.<< https://www.sciencedirect.com/science/article/pii/S0301462204003540 >>.

The solution of the cubic equation is analogous to the solution presented above and was implemented in Origin-C code, which is available as an Origin function definition file (fdf) from https://github.com/niekbuurmah2o/equilibrium-models-for-bimetallic-lanthanide-coiled-coil, as follows.

```
double hostfree, Afree, Bfree, Abound, Bbound;
```

```
double a,b,c,d;

double f,g,h;

double i,j,k;

double L,M,N,P;


//Define a, b, c and d

a = KA*KB;

b = KA+KB+KA*KB*(binderA+binderB-host);

c = 1 + KA*binderA + KB*binderB - (KA + KB)*host;

d = -1*host;


// see http://www.1728.org/cubic2.htm for solution to cubic


f = ((3*c/a)-(b^2/a^2))/3;

g = ((2*(b^3)/(a^3))-(9*b*c/(a^2))+(27*d/a))/27;

h = ((g^2)/4)+((f^3)/27);


i = sqrt((g^2/4)-h);

j = i^(1/3);

k = acos(-1*(g / (2*i)));


L = -1*j;

M = cos(k/3);

N = sqrt(3)*sin(k/3);

P = -1*(b/(3*a));
```

```
// FEBS letters 1995 Zhi-Xin Wang and Analytical Biochmesitry 2000 Bent
Sigurskjold show that with reasonable numbers, only one of the three
solutions is the reasonable one and this always seems to be the same one

hostfree = 2*j*cos(k/3)-(b/(3*a));
Abound = hostfree*binderA / ((1/KA) + hostfree);
Bbound = hostfree*binderB / ((1/KB) + hostfree);
Afree = binderA - Abound;
Bfree = binderB - Bbound;

// Compound B is the signal carrier as this is held constant in our
titrations.
// Compound A without signal is titrated in in this case.
// Change Bbound to Abound in the line below if the compound titrated in is
the signal carrier.

signal = background + response*Bbound;
```

EPR: The distance between the bound Gd^{3+} for each of the peptides was measured using the EPR spectroscopic method of double electron-electron resonance (DEER) in the frozen state.[11-13] This experiment measures the dipolar coupling between the Gd^{3+} pairs and since this is dependent on the distance between the centres, the distance distribution can be extracted. Gd^{3+} is a high spin ion (S = 7/2) and has been widely employed for measuring distances above 4 nm.[14] Particularly it has been used for measuring distances between Gd^{3+} spin labels on biomolecules within cells. However, for distances below 4 nm, artefacts associated with flip-flop effects are observed with the usual DEER measurement approaches. These artefacts lead to complications in determining accurate distances and distributions. We therefore employed the W-band high

power and wide bandwidth HiPER spectrometer.[15] This spectrometer allows for DEER measurements of LS2-1,3 and LS2-1,4 to be taken where both the pump and probe frequencies in the DEER pulse sequence were positioned to avoid the intense central transition of the Gd^{3+} absorption spectrum. This configuration effectively suppresses most artefacts and significantly enhances the accuracy of the extracted distances.[16]

Sample preparation: Peptides were dissolved in D_2O and their monomeric concentration determined with UV-Vis at 280 nm using an extinction coefficient of 11380 M^{-1} cm^{-1} (based on sequences containing two Trp). Final samples concentrations were 20 μM trimer and 40 μM Gd^{3+} (from $GdCl_3$ stock in D_2O) in 10 mM HEPES pH 7.0, D_2O and contained 50% by volume glycerol-d_8. Samples for HiPER (W-band) were 90 μL in a FEP tube and for the Q-band were 60 μL in a quartz tube. Samples were frozen in liquid nitrogen prior to loading. LS2-1,5 required further annealing through one temperature cycle (to 180 K) *in situ* to create a good glass.

All EPR experiments were carried out at 10 K. W-band (94 GHz) measurements were carried out on a home-built 1 kW pulsed spectrometer, widely known as HiPER, which has been upgraded using an arbitrary waveform generator (AWG) (Keysight M8190A) and incorporates a continuous flow helium cryostat (CF 935). HiPER operates with a wideband non-resonant induction mode sample holder and has been described in detail elsewhere.[15] DEER measurements were carried out using the AWG except for the LS3-1,6 sample, which was measured before the upgrade, where two separate microwave sources have been used for the pump and observer pulses.

The Q-band (34 GHz) experiments on LS3-1,6 were carried out with a Bruker ELEXSYS E580 with a cryogen free variable temperature cryostat from Cryogenic Ltd. The measurements were performed with a high power TWT amplifier (150 W) and a cylindrical resonator (ER 5106QT-2w). The MPFU and ELDOR channels were employed for DEER measurements.

Echo-detected field swept spectra (ED-FS) were obtained with a $\pi/2$-τ-π-τ-echo sequence, rectangular pulses and a 2-step phase cycle. In HiPER LS2-1,3, LS2-1,4, LS2-1,5 were measured with $t_{\pi/2} = t_\pi = 10$ ns, $\tau = 300$ ns, 3 kHz (LS2-1,3) or 4 kHz (LS2-1,4, LS2-1,5) repetition rate. In

HiPER LS3-1,6 was measured with band $t_{\pi/2}$ = 5 ns, t_{π} = 10 ns and τ = 248 ns and a 1 kHz repetition frequency. At Q-band $t_{\pi/2}$ = 16 ns, t_{π} = 32 ns and τ = 400 ns and a 1 ms shot repetition time was used. The ED-FS results for the four peptides at W-band are presented in Figure S21. FWHH are reported in the figure caption. ED-FS are also presented in Figure S23 in relation to the settings of the pump and observer pulses, here the Q-band ED-FS is also presented.

Echo-detected decay curves which indicate the phase memory time (T_m) of the Gd^{3+} were measured with the same Hahn echo sequence and pulse settings including two-step phase cycle but with the τ stepped. The measurements were taken on HiPER for the LS2 peptides. These measurements had the pulse sequence positioned at the most intense Gd^{3+} absorption (on the Gd^{3+} central transition) and offset to the high field side for LS2-1,3 (11.5 mT) and LS2-1,4 (10 mT) and to the low field side for LS2-1,5 (11 mT). The echo decay curves are shown in Figure S22. The LS2-1,4 and LS2-1,5 data overlap well and the LS2-1,3 T_m appears longer. As expected for Gd^{3+} the T_m away from the CT is shorter than when measured on the CT.
Since the observation of the low modulation depths in the DEER time traces led to the hypothesis that there is some level of incomplete binding of Gd^{3+} in our peptides we suggest that the FWHH of the ED-FS and the T_m curves are indicative of the actual sample, rather than characterizing these parameters for the completely bound peptides. However, the trends in these values across the samples remain interesting.

Four-pulse DEER was measured using the conventional sequence $\pi/2_{vobs} - \tau_1 - \pi_{vobs} - (\tau_1 + t) - \pi_{pump} - (\tau_2 - t) - \pi_{vobs} - \tau_2 -$ echo.[11-13, 17, 18] The pulses were rectangular or truncated hamming sinc, HS (truncation value *n* given as HSn). The experimental parameters were systematically optimized for each sample to achieve the maximum modulation depth. As a result, different setups were employed with HiPER for the LS2 samples. HiPER data for the LS2 peptides was collected using an offset frequency filtering as previously described and therefore no phase cycling was required.[19] LS3-1,6 was measured with HiPER by integrating the most intense 30% of the echo without a phase cycle.

The Q-band measurement utilised τ_1 averaging using 5 steps with a size of 24 ns and the signal was obtained by integrating 32 ns of the maximum of the refocussed echo an 8-step phase cycle was used.

DEER time traces are shown in Figures S24-S28 and other variable settings are provided in Tables S4-S7.

HiPER DEER were obtained for LS2-1,3 and LS2-1,4 by setting the pump pulse on the top or side of the central transition (Figure S23). The effect of the different pulse set ups are seen on the DEER time traces and resulting distance distributions in Figures S26 (LS2-1,3) and S27 (LS2-1,4). Figure S27 illustrates that by pumping the CT, the modulation depth of the DEER signal is increased but that by involving the CT in either the pump or observer pulses distorts the time signal. The time traces shown in the main paper utilised the ability of HiPER to have both pump and observer pulses away from the central transition which avoided the mixing in of pseudo-secular dipolar terms which would manifest as a broadening of the interpretable distance distribution.

LS3-1,6 was measured by HiPER prior to upgrades which increased stability of phase drifts and data collection. It is therefore seen that the background appears to increase (Figure S28) which is not physically reasonable. This was a known caveat at the time and so we also measured the sample at Q-band, also shown in Figure S28 and this provided a very similar result.

DEER data analysis was carried out in two ways. DeerAnalysis2022 with Tikhonov regularization was used for all DEER time traces.[20] Zero time, background start and Tikhonov regularization parameter, background correction (3D homogeneous) were determined by the software except LS3-1,6 measured at Q-band. These parameters are reported in Tables S4-S7. A measure of the uncertainty of the results was determined using Validation. Default parameters for white noise and background correction were used to create a fine grid of 110 points, except for LS2-1,3 where a background start value of 50 ns was used. Figure S25 presents the fits and extracted distance distributions while the most probable distances are presented in the main paper (Table 2).

The data was also analysed using DeerLab 1.1.4 (Python 3.12) to allow for a straightforward model approach to be applied.[21] This is useful since the DEER time traces have a very small signal-to-noise due to the small modulation depth, and this approach helps to stabilise the calculations to obtain the required information i.e. the most probable distance between Gd^{3+} centres. Here, the zero time was determined by DeerLab and other default values were used. The parameterized dd_gauss model was used, and in all cases the distance range was 1.5 to 8 nm. The results with default start values and errors are shown in Figure S24. It can be seen by visual inspection of the red simulated line on the experimentally determined time trace that the width of the Gaussian tends to be broader than the time traces suggest. Therefore, we chose to also model with fixed standard deviation for the Gaussian of 0.2 nm. This value was chosen arbitrarily but was informed by the narrower distributions given by the freely varying standard deviation Gaussian fit (Figure S24). These results are presented in the main paper, Figure 2 alongside the means of the best fit Gaussian. Table S8 presents the results output from DeerLab for both methods.

The observed time traces and the model-free analysis indicate the distribution is broader for LS2-1,5 than the other peptides. This is likely caused by two reasons. The first is the nature of the frozen ensemble of structures for the LS2 peptide with both Gd^{3+} at the termini. This larger ensemble of structures is also consistent with the slightly larger deviation from the hypothesized distance (4.0 nm in this case) than the other measured peptides. The second reason is that there may be a residual broadening of the distribution due to the flip-flop artefacts. Unfortunately, the poor signal-to-noise ratio due to low signal and limiting phase memory time did not allow for a good enough DEER measurement away from the central transition to determine this factor for LS2-1,5.

The measurements were all challenging from the point of view of the modulation depth, i.e. how much of the signal is affected by the dipolar coupling, which impacts on the signal-to-noise. We would expect small values for Gd-Gd measurements and especially where the central transition needs to be avoided. However, they were smaller than expected from other

measurements we have performed with Gd^{3+} spin labels, and this may be due to some issues freezing the dynamic N-terminal Gd^{3+} site.

X-ray crystallography: Crystals of xLS2”-2,3 were grown using sitting-drop vapor-diffusion at 18 °C from a 0.4 μL peptide stock solution (4 mM peptide monomer, 8 mM $TbCl_3$) mixed with

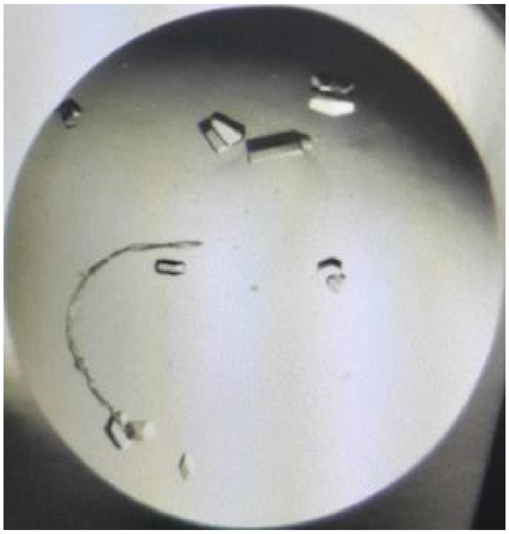

0.4 μL precipitant solution (0.1 M magnesium chloride hexahydrate, 0.1 M sodium HEPES buffer pH 7, 15% PEG 4000). Crystals, see image below, belonging to space group *P6* were obtained and cryoprotected with 30% glycerol prior to flash freezing and storage in liquid nitrogen. Data was collected at Diamond Light Source utilising beam I04. Deposited as PDB 9SOM.

Native mass spectrometry (MS): Lyophilized LS2-1,5 was reconstituted in 50 mM ammonium acetate pH 6.8. Metal stock solutions (10 mM) were initially prepared in MilliQ water and then diluted into 50 mM ammonium acetate pH 6.8 immediately before use. LS2-1,5 was analysed at a final concentration of 90 μM peptide monomer (30 μM trimer). LS2-1,5 was incubated alone, with Tb(III), Yb(III), or a Tb(III)-Yb(III) mixture, at 0.5 and 1.0 equivalents, 1.0 equivalents, and 1.0-1.0 and 2.0-1.0 equivalents per LS2-1,5 trimer, respectively. For example; 90 μM LS2-1,5 (monomer) was incubated with 30 μM Tb(III) and 30 μM Yb(III) to achieve a final LS2-1,5-Tb-Yb

mixture containing 1.0 equivalents of each component. All mixtures were incubated at room temperature for 30 minutes, followed by immediate native MS analysis.

Native MS measurements were performed using an Orbitrap Ascend Tribrid Structural Biology mass spectrometer (Thermo Fisher Scientific, San Diego, CA). Samples were introduced into the mass spectrometer using gold-coated borosilicate needles, prepared in-house. Nano-electrospray ionization was performed in positive ion mode, using a voltage of 1.0 kV, 250°C source temperature. 'Intact Protein' and 'High Pressure' modes were used throughout with the in-source fragmentation set to zero, and RF lens 100%. Mass spectra were acquired with a normalised automatic gain control target of 100%, maximum ion injection of 100 ms, and 3 micro-scans were acquired per scan cycle. Ions were detected in the Orbitrap at a resolution of 15,000 at *m/z* 200. The mass range was set to 400 – 3000 *m/z*.

Mass spectra were averaged for 3 minutes using Xcalibur *v*.4.2 software (Thermo Fisher Scientific). The observed molecular weights were calculated from a minimum of 2 charge states. For Tb-, Yb-, and Tb/Yb-bound complexes, assuming the metal (3+ charge) and H+ both contributed to the protonation states observed in the mass spectrum.

2. Figure S1:

A)

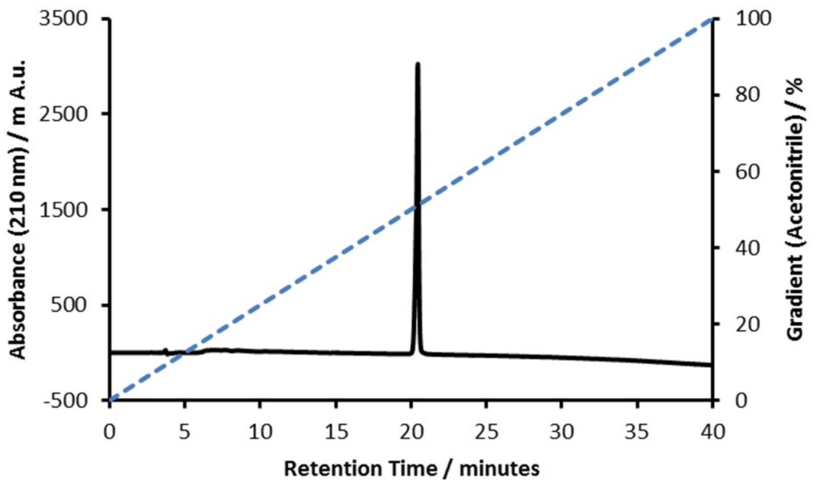

B)

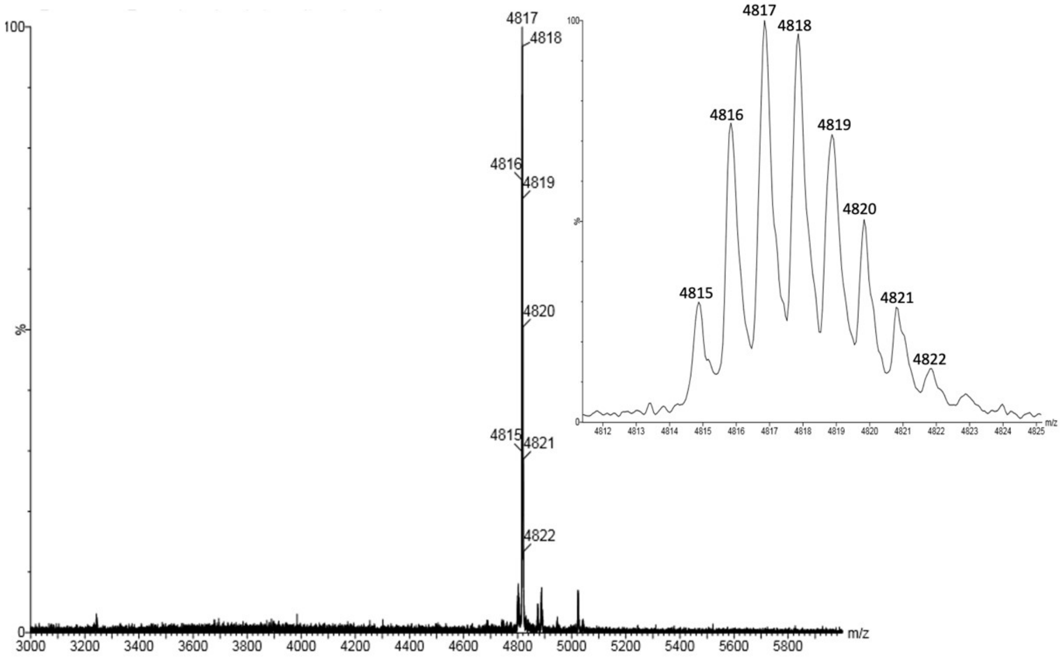

Figure S1. A) C18-analytical HPLC trace of purified LS2-1,2, using a linear gradient from 0-100% acetonitrile in water with 0.05% TFA (blue dashed line) over 40 minutes, monitored at a detection wavelength of 210 nm. B) MALDI mass spectrum of purified LS2-1,2 with inset showing the isotope distribution of the +1 charge peak.

3. Figure S2:

A)

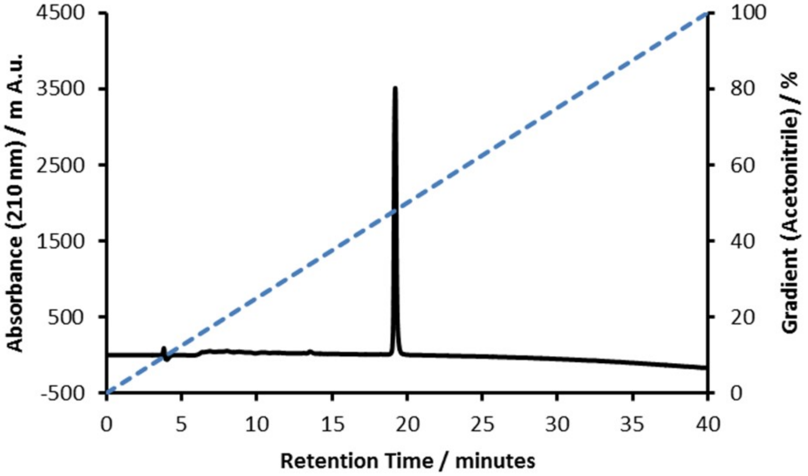

B)

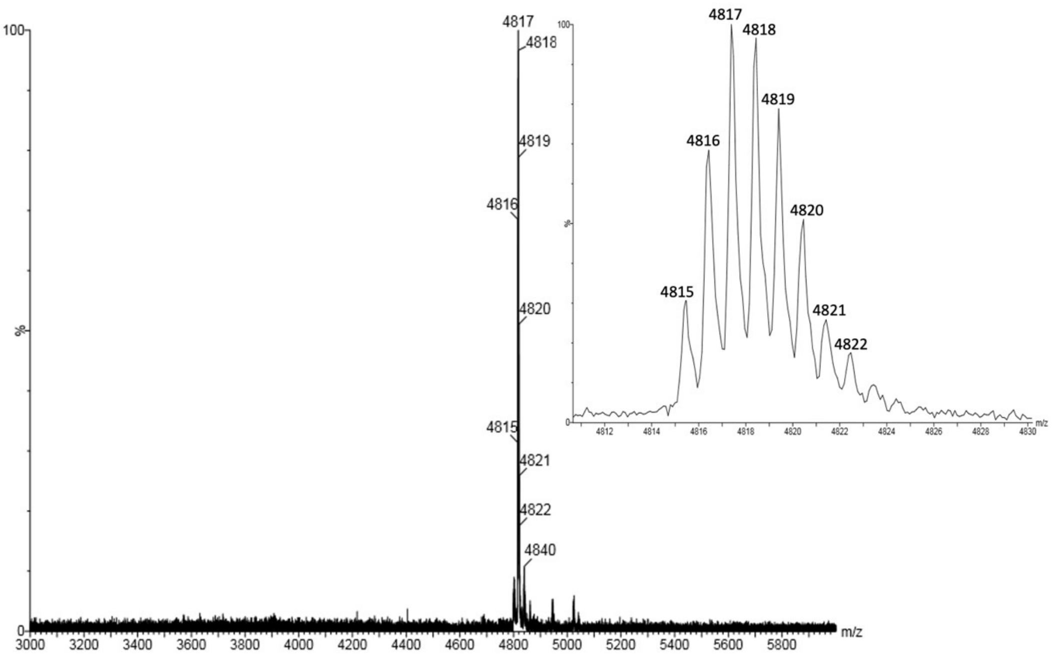

Figure S2. A) C18-analytical HPLC trace of purified LS2-1,3, using a linear gradient from 0-100% acetonitrile in water with 0.05% TFA (blue dashed line) over 40 minutes, monitored at a detection wavelength of 210 nm. B) MALDI mass spectrum of purified LS2-1,3 with inset showing the isotope distribution of the +1 charge peak.

4. Figure S3:

A)

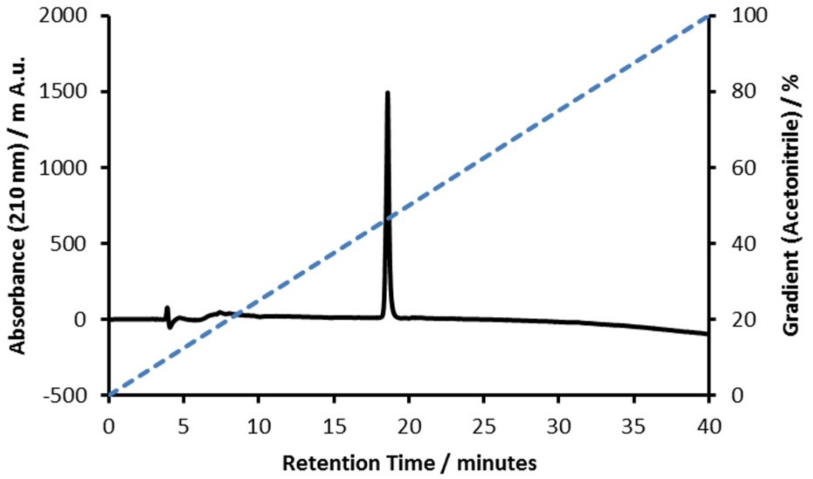

B)

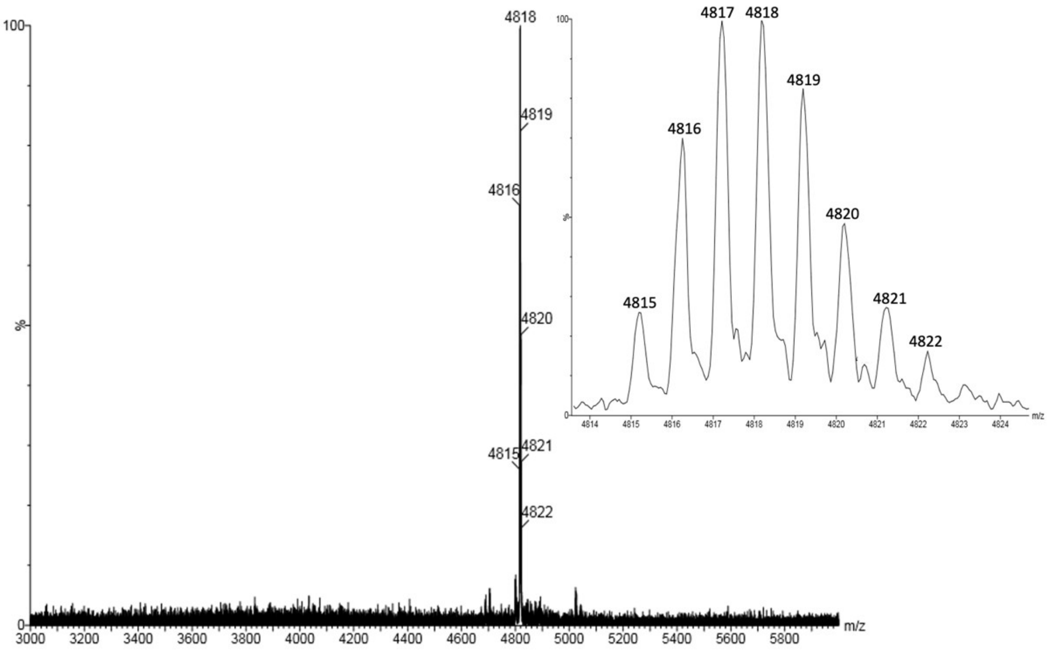

Figure S3. A) C18-analytical HPLC trace of purified LS2-1,4, using a linear gradient from 0-100% acetonitrile in water with 0.05% TFA (blue dashed line) over 40 minutes, monitored at a detection wavelength of 210 nm. B) MALDI mass spectrum of purified LS2-1,4 with inset showing the isotope distribution of the +1 charge peak.

5. Figure S4

A)

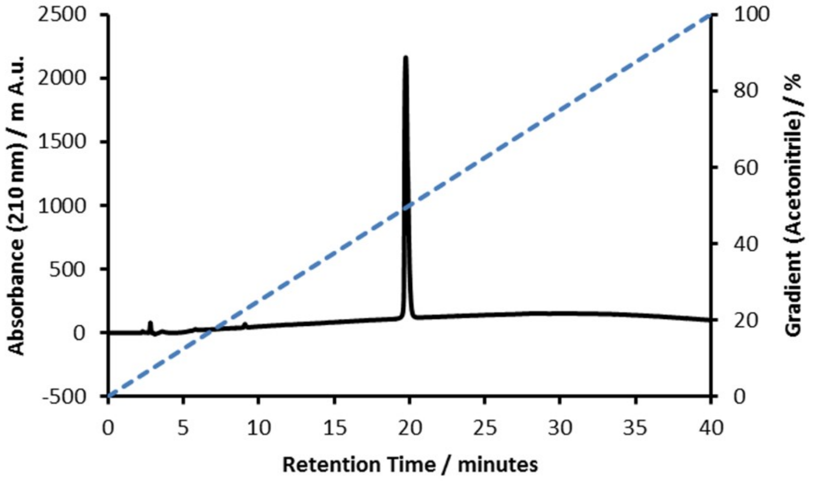

B)

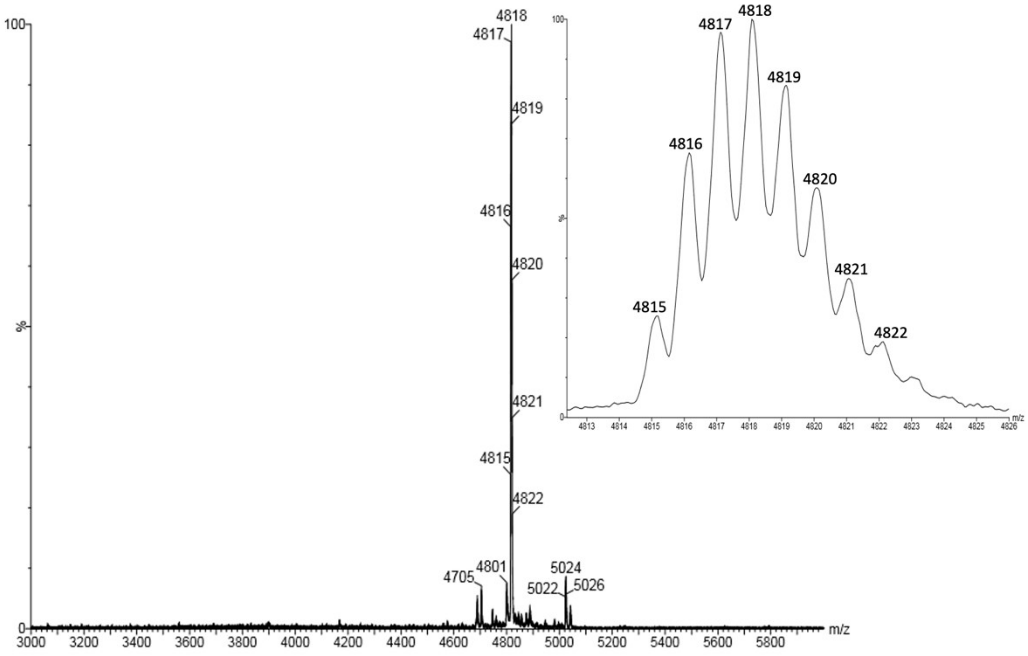

Figure S4. A) C18-analytical HPLC trace of purified LS2-1,5, using a linear gradient from 0-100% acetonitrile in water with 0.05% TFA (blue dashed line) over 40 minutes, monitored at a detection wavelength of 210 nm. B) MALDI mass spectrum of purified LS2-1,5 with inset showing the isotope distribution of the +1 charge peak.

6. Figure S5

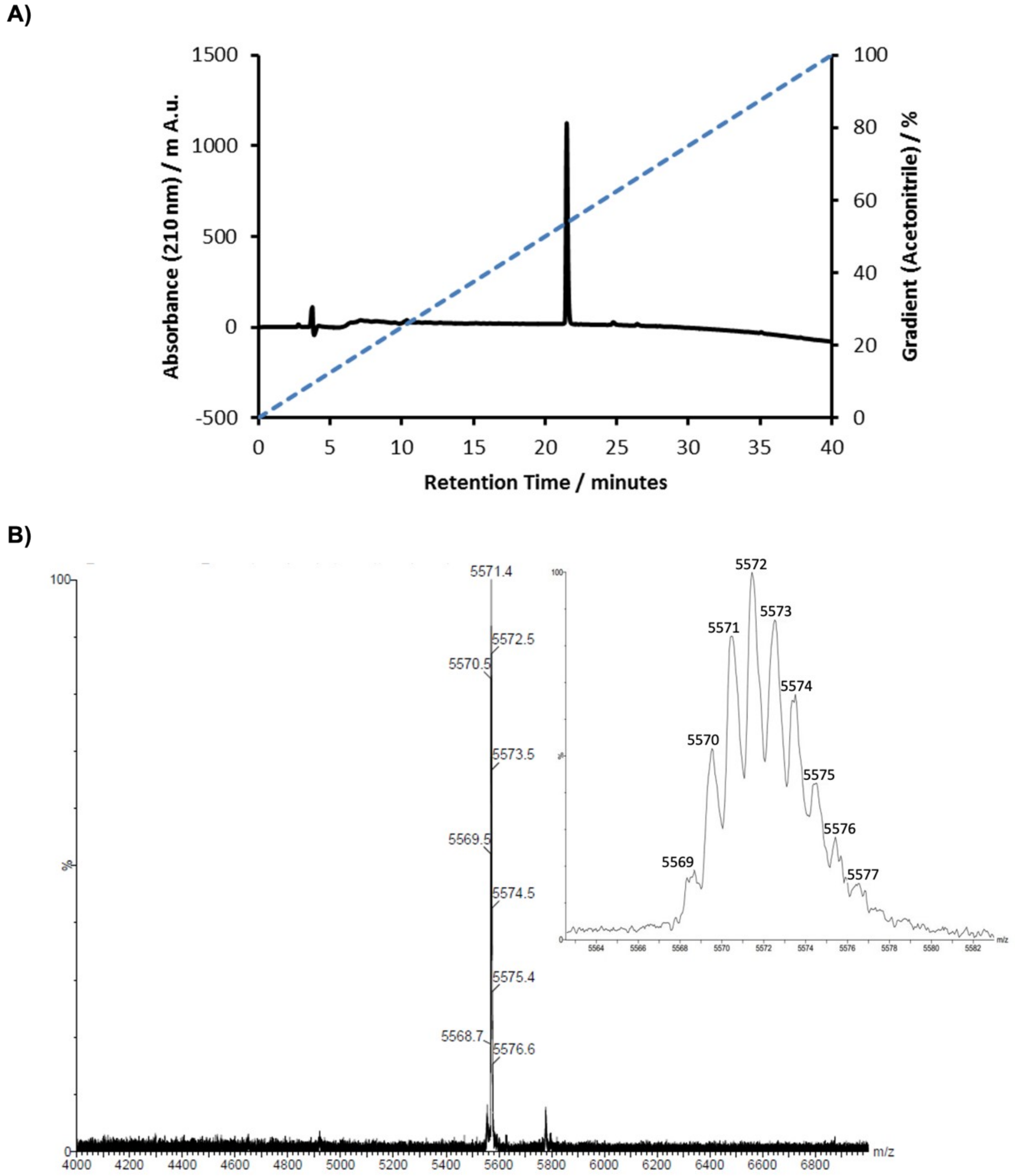

Figure S5. A) C18-analytical HPLC trace of purified LS3-1,6, using a linear gradient from 0-100% acetonitrile in water with 0.05% TFA (blue dashed line) over 40 minutes, monitored at a detection wavelength of 210 nm. B) MALDI mass spectrum of purified LS3-1,6 with inset showing the isotope distribution of the +1 charge peak.

7. Figure S6

A)

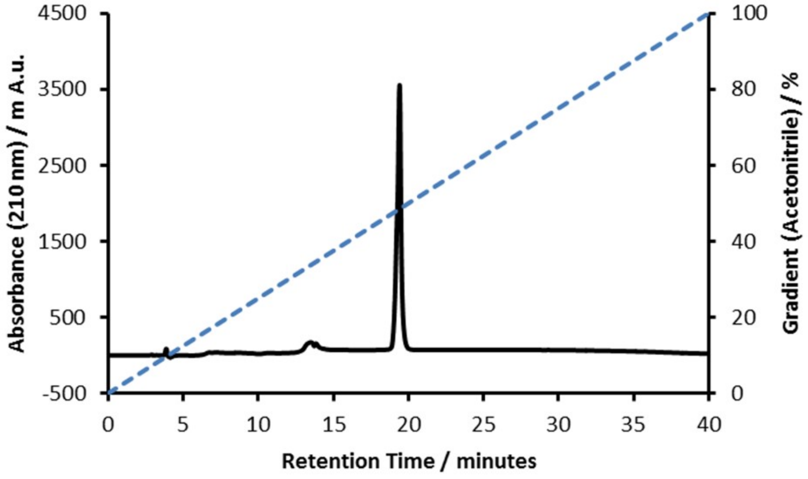

B)

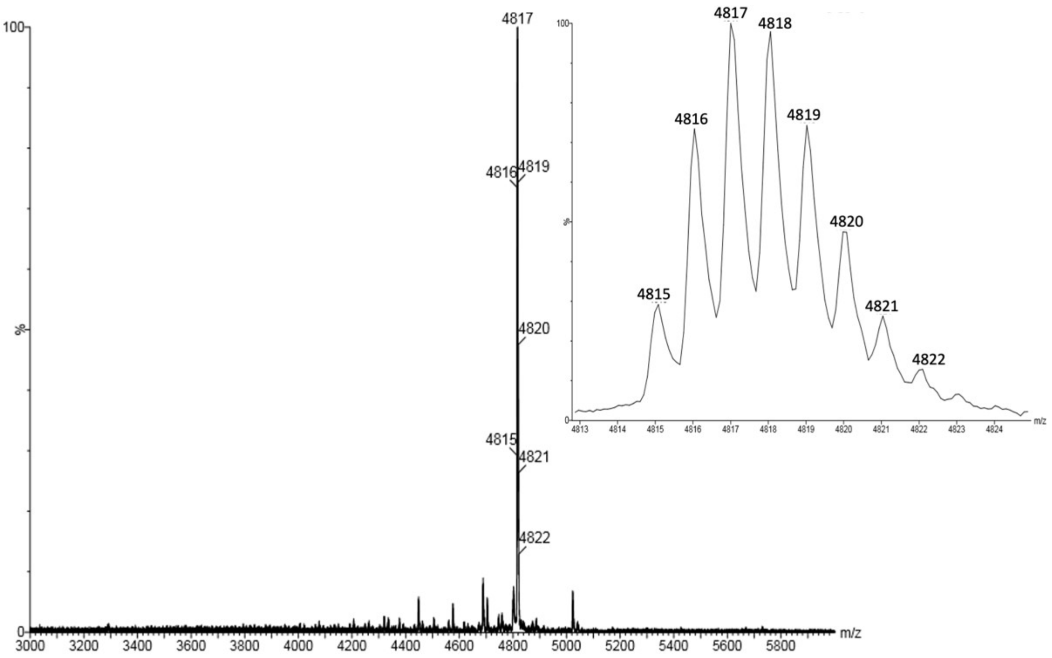

Figure S6. A) C18-analytical HPLC trace of purified LS2'-2,3, using a linear gradient from 0-100% acetonitrile in water with 0.05% TFA (blue dashed line) over 40 minutes, monitored at a detection wavelength of 210 nm. B) MALDI mass spectrum of purified LS2'-2,3 with inset showing the isotope distribution of the +1 charge peak.

8. Figure S7

A)

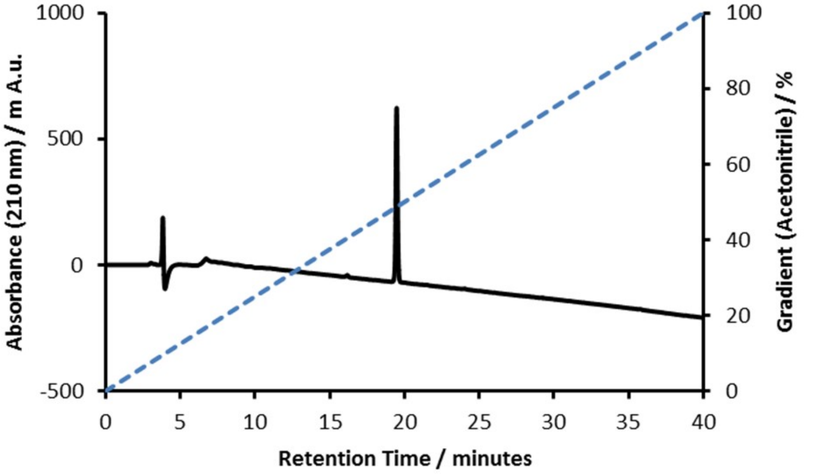

B)

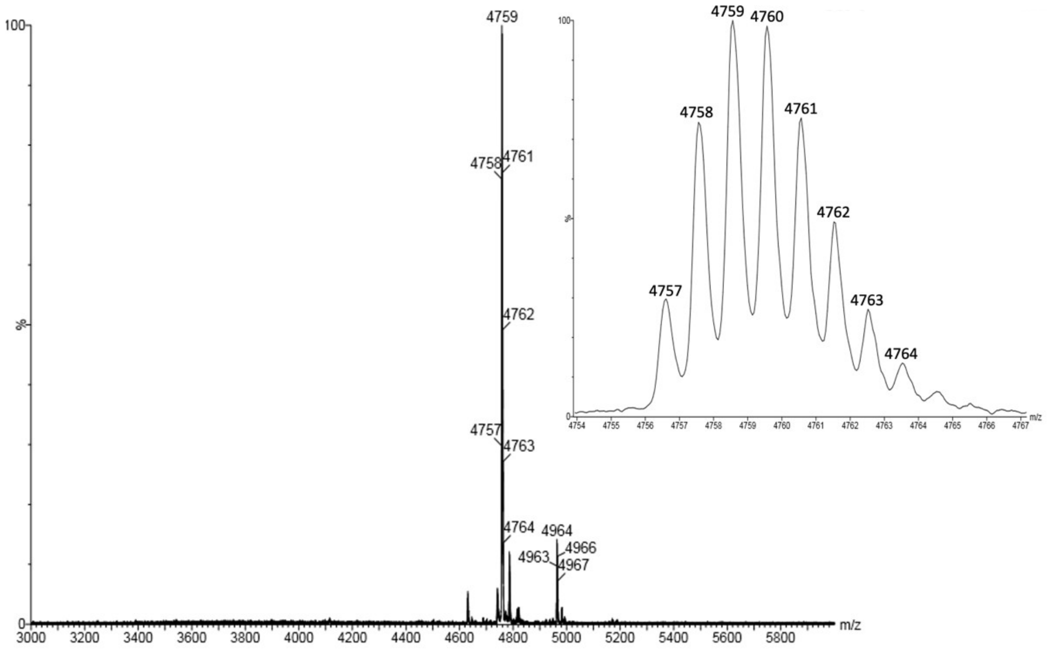

Figure S7. A) C18-analytical HPLC trace of purified LS2-1,5(7Q), using a linear gradient from 0-100% acetonitrile in water with 0.05% TFA (blue dashed line) over 40 minutes, monitored at a detection wavelength of 210 nm. B) MALDI mass spectrum of purified LS2-1,5(7Q), with inset showing the isotope distribution of the +1 charge peak.

9. Figure S8

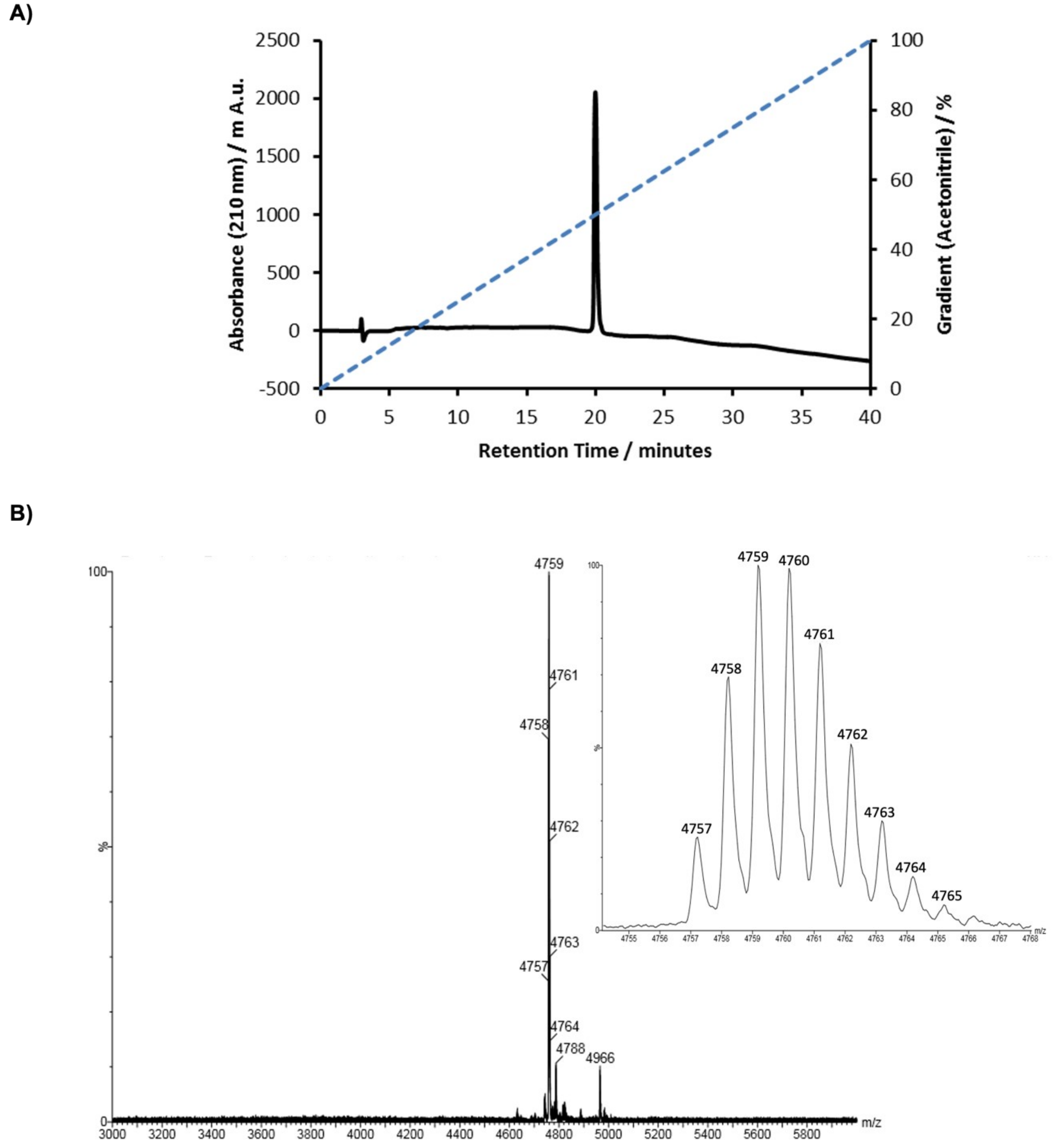

Figure S8. A) C18-analytical HPLC trace of purified LS2-1,5(35Q), using a linear gradient from 0-100% acetonitrile in water with 0.05% TFA (blue dashed line) over 40 minutes, monitored at a detection wavelength of 210 nm. B) MALDI mass spectrum of purified LS2-1,5(35Q), with inset showing the isotope distribution of the +1 charge peak.

10. Figure S9

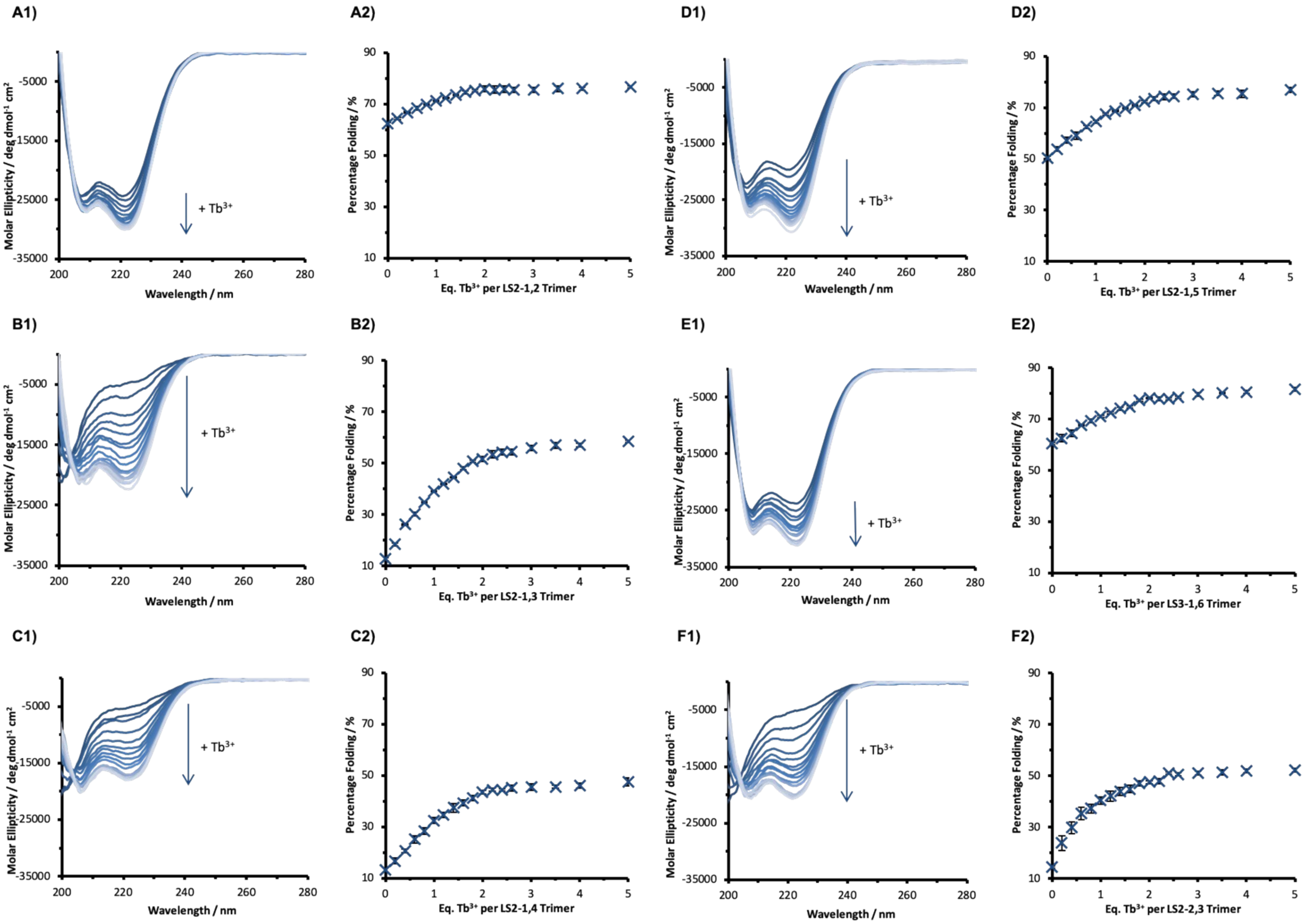

Figure S9. Tb^{3+} binding titrations monitored by 1) CD and 2) plots showing percentage folded (based on molar ellipticity at 222 nm), as a function of Tb^{3+} equivalents per trimer, for 30 µM peptide monomer solutions of A) LS2-1,2, B) LS2-1,3, C) LS2-1,4, D) LS2-1,5, E) LS3-1,6 and F) LS2'-2,3, in 10 mM HEPES buffer pH 7.0.

11. Table S1.

Table S1. Summary of % folded values

Peptide	% Folded[a] Apo-	1 eq. Tb^{3+}	2 eq. Tb^{3+}	5 eq. Tb^{3+}
LS2-1,2	62 ± 1	71 ± 1	76 ± 1	77 ± 1
LS2-1,3	13 ± 1	40 ± 1	52 ± 2	59 ± 2
LS2-1,4	13 ± 1	32 ± 1	44 ± 1	47 ± 2
LS2-1,5	50 ± 1	65 ± 1	72 ± 1	77 ± 1
LS2-1,5(7Q)	50	66	72	76
LS2-1,5(35Q)	53	64	73	80
LS3-1,6	61 ± 1	71 ± 1	78 ± 1	82 ± 1
LS2'-2,3	15 ± 1	40 ± 2	48 ± 1	52 ± 1

[a] Percentage folding determined by CD spectroscopy for 30 μM peptide monomer +/- 10, 20 and 50 μM $TbCl_3$ in 10 mM HEPES buffer pH 7.0. Errors are the standard deviation of three independent repeats.

12. Figure S10

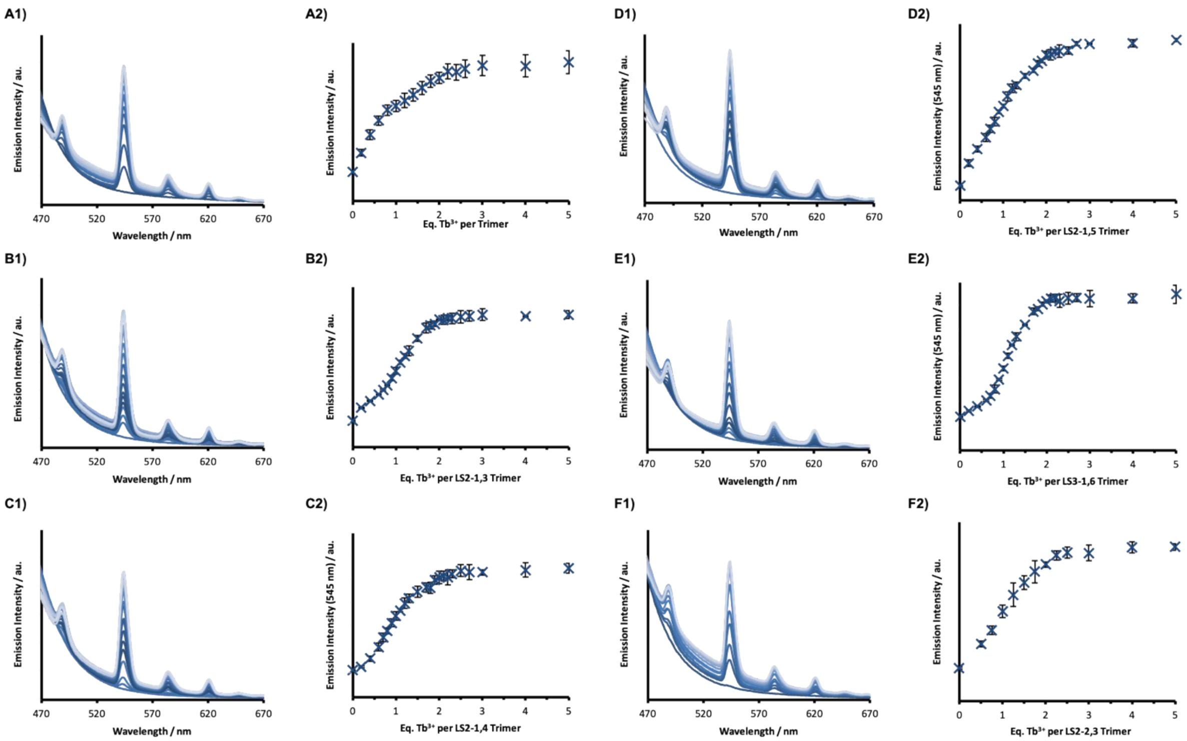

Figure S10 Tb^{3+} binding titrations monitored by 1) luminescence and 2) plots showing emission intensity (based on the integration of the 545 nm Tb^{3+} emission peak), as a function of Tb^{3+} equivalents per trimer, for 30 μM peptide monomer solutions of A) LS2-1,2, B) LS2-1,3, C) LS2-1,4, D) LS2-1,5, E) LS3-1,6 and F) LS2'-2,3, in 10 mM HEPES buffer pH 7.0. Data is a result of the average of three repeats where the error bars represent the standard deviation, λ_{ex} = 280 nm.

13. Figure S11

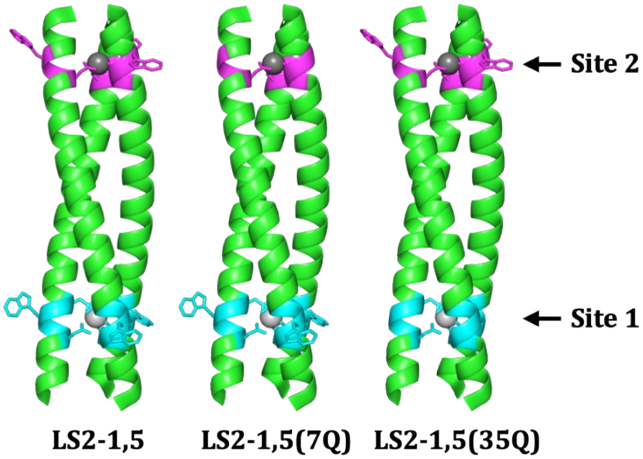

Figure S11 Models of the dual Ln^{3+} binding coiled coil, LS2-1,5 (left), and the controls LS2-1,5(7Q) (middle) and LS2-1,5(35Q) (right), which only feature a single Trp layer capable of exclusively reporting on Tb^{3+} binding to site 1 and 2, respectively. The main chain atoms are represented as helical ribbons; binding sites (1 cyan; 2 pink) are highlighted; coordinating residues and Trp side chains are shown in stick form; and the Ln^{3+} ions as grey spheres.

14. Figure S12

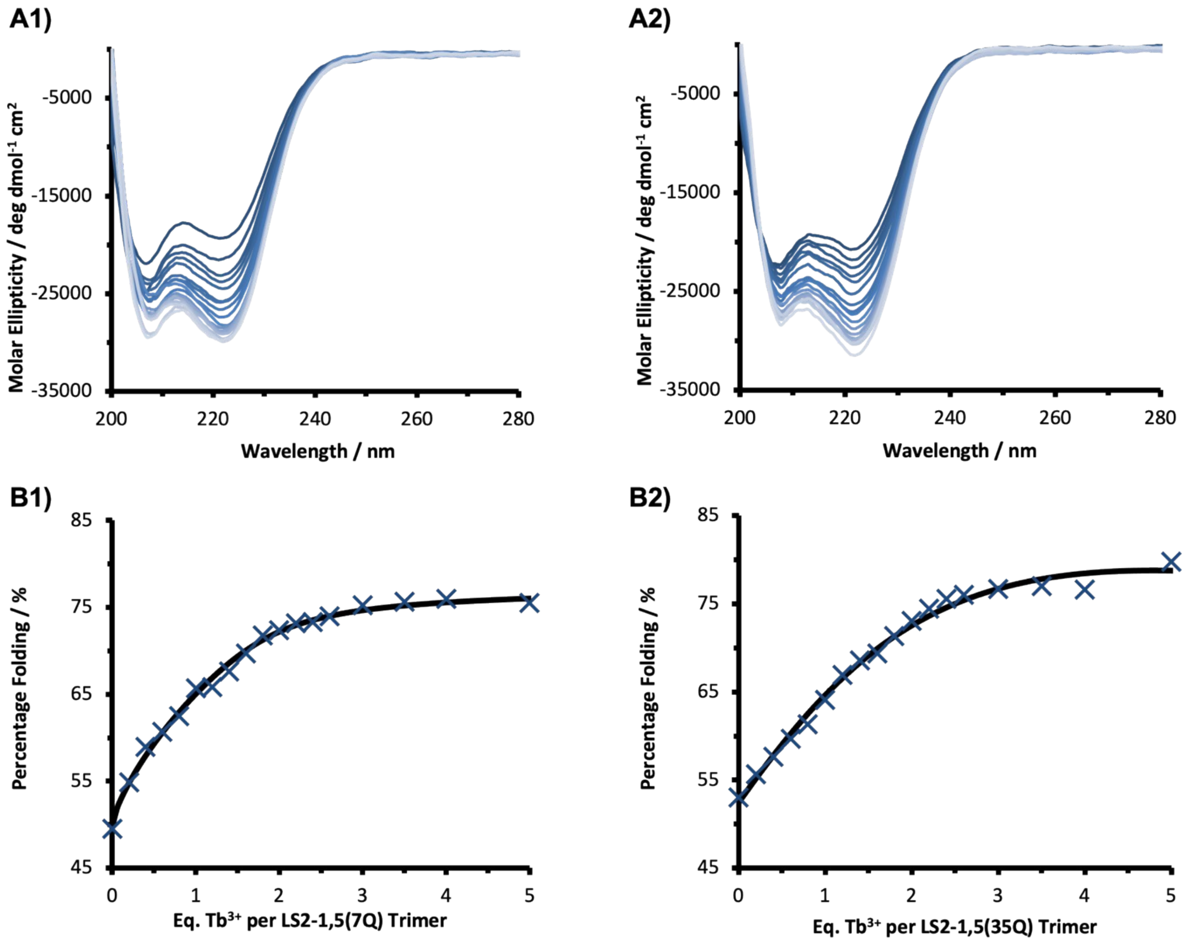

Figure S12 A) Tb^{3+} binding titrations monitored by CD into 30 μM 1) LS2-1,5(7Q) or 2) LS2-1,5(35Q) monomer in 10 mM HEPES buffer pH 7.0. B) Plots of percentage folded, based on molar ellipticity at 222 nm, as a function of Tb^{3+} equivalents per trimer.

15. Figure S13

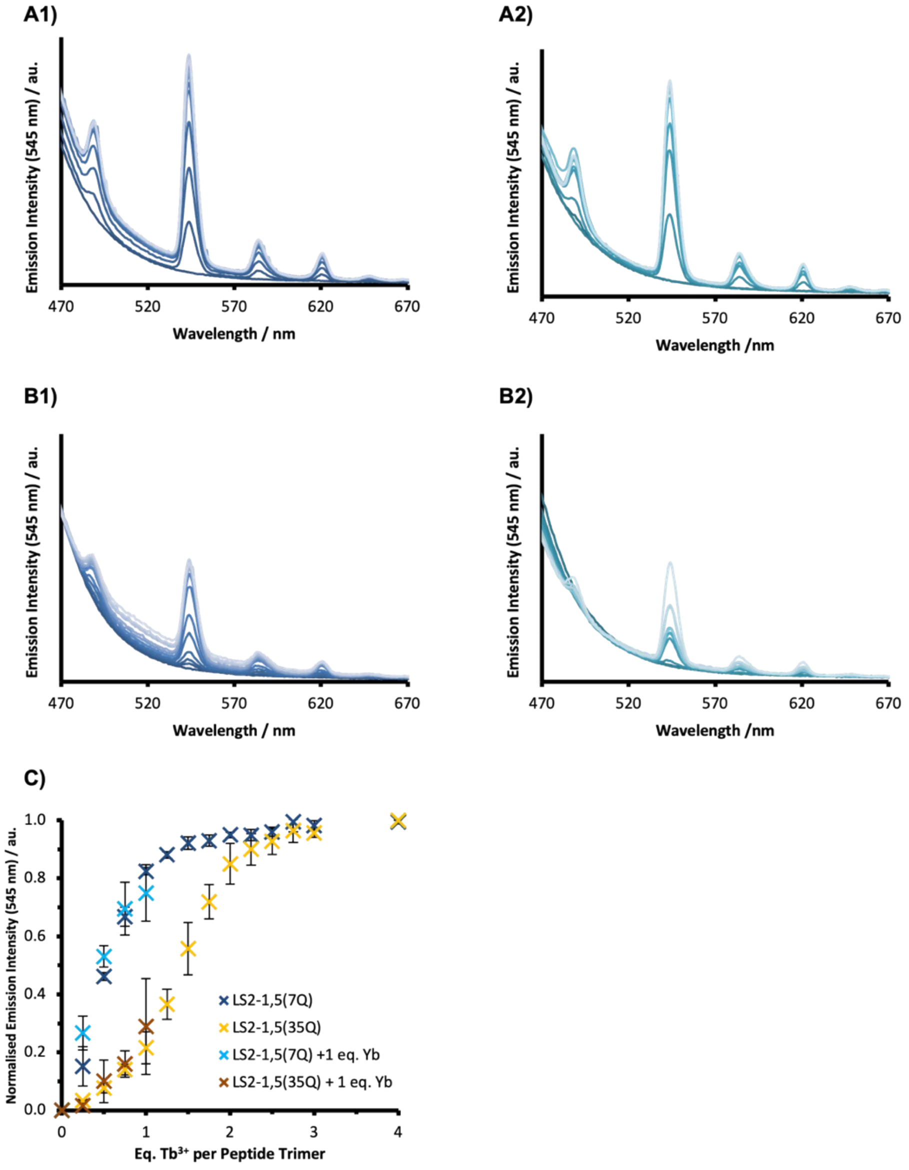

Figure S13 Representative Tb^{3+} binding titrations monitored by luminescence of 30 µM peptide monomer solutions of A) LS2-1,5(7Q) and B) LS2-1,5(35Q) in 10 mM HEPES buffer pH 7.0 in the 1) absence and 2) presence of 1 equivalent of Yb^{3+} per trimer. C) Plot of emission intensity, based on the 545 nm Tb^{3+} emission peak, as a function of Tb^{3+} equivalents per trimer. Data are the average of three repeats where the error bars represent the standard deviation. λ_{ex} = 280 nm.

16. Figures S14-S16

Global fit of the binding model to all nine titration curves, with fully optimisable background signals for each individual titration with the cooperativity factor restricted to a values of 1, 0.1 and 10 are shown in Figures S14-S16.

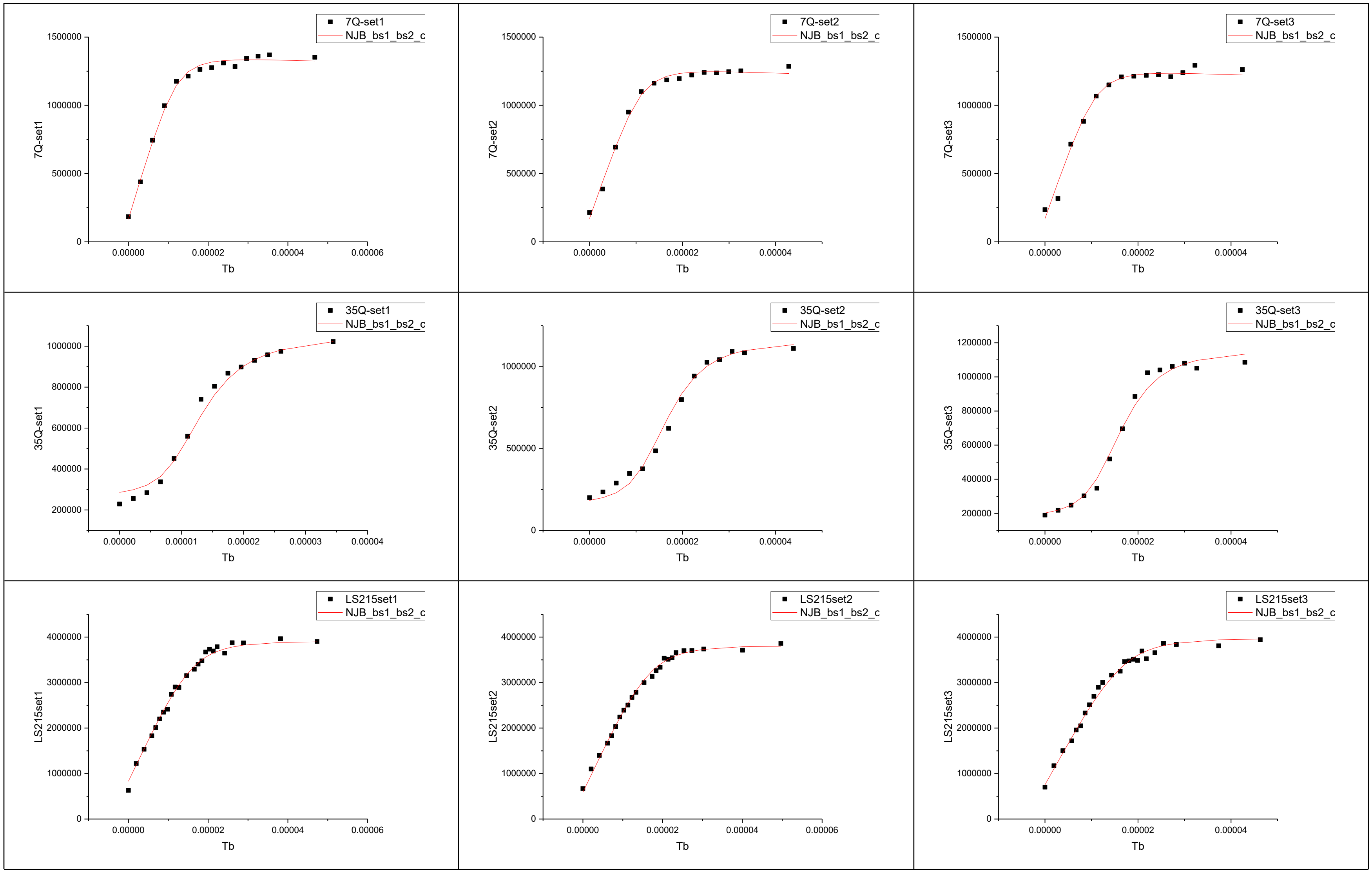

Figure S14 Global fit of the model to all nine titration curves with the cooperativity factor restricted to a value of 1.

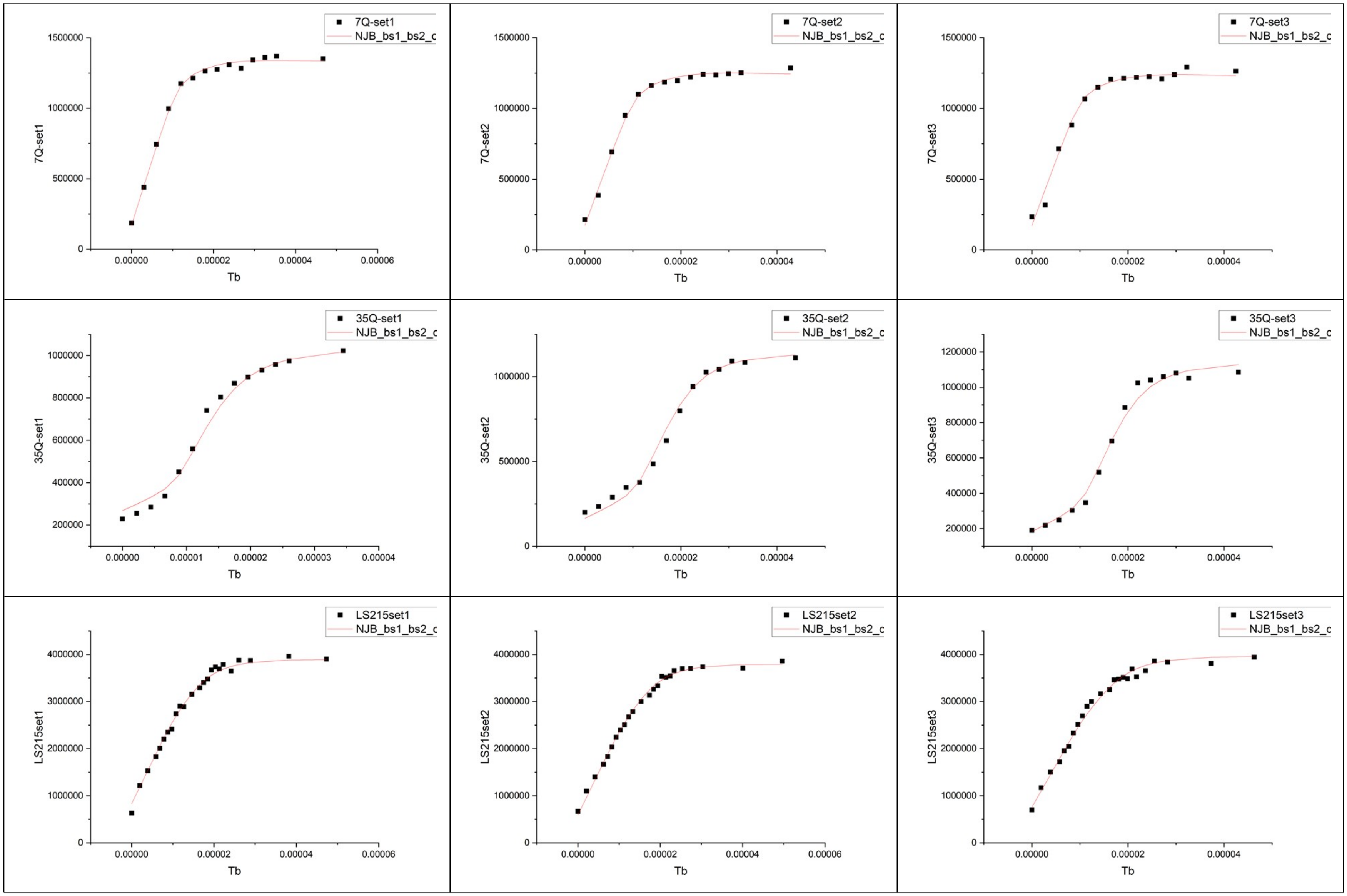

Figure S15 Global fit of the model to all nine titration curves with the cooperativity factor restricted to a value of 0.1.

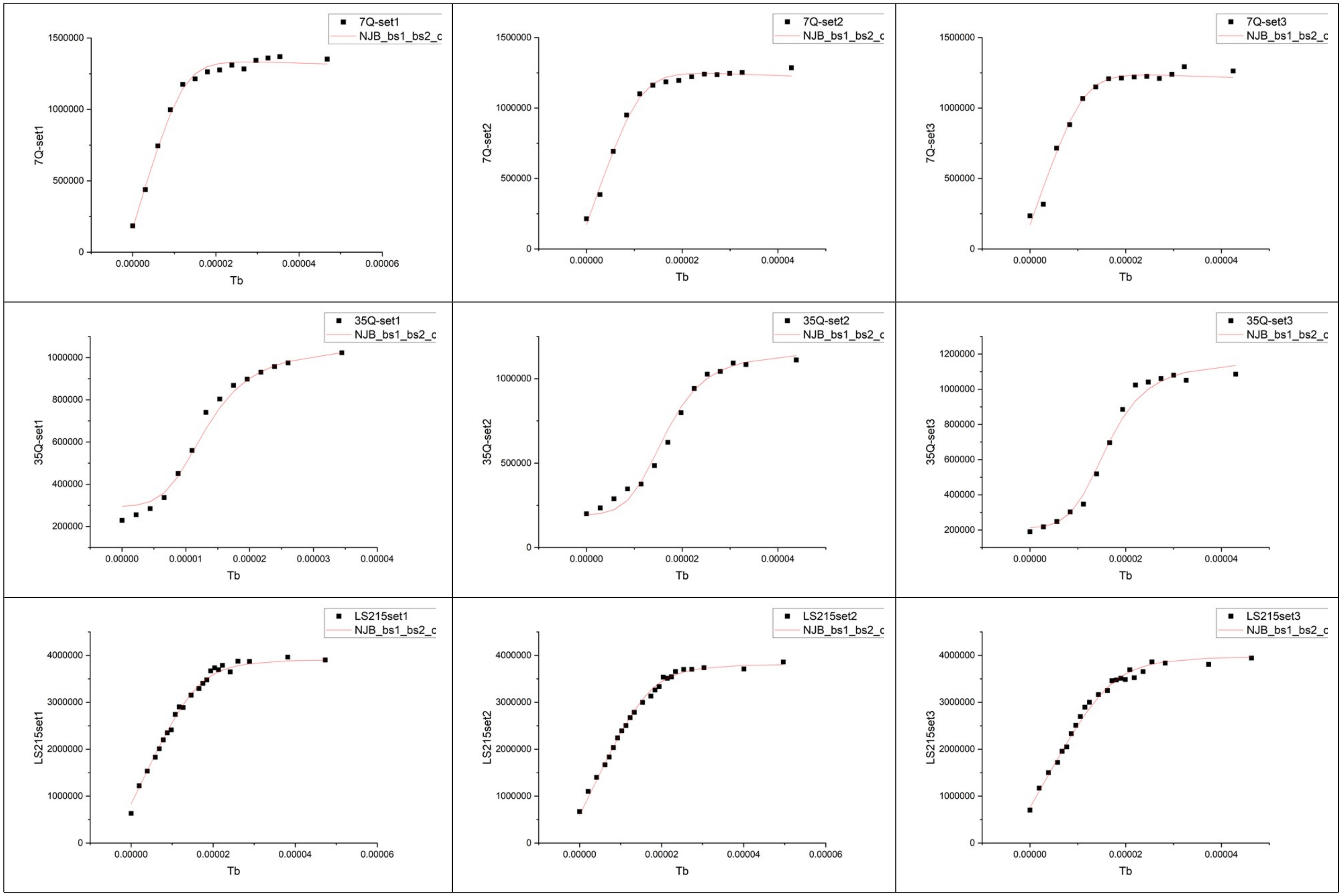

Figure S16 Global fit of the model to all nine titration curves with the cooperativity factor restricted to a value of 10.

17. Table S2. Optimised parameters from data analysis

Table S2. Fitting parameters for binding model

		cooperativity optimised			cooperativity = 0.01			cooperativity = 0.1			**cooperativity = 1**			cooperativity = 10			cooperativity = 100		
K_1		9.23E8	±	5.62E9	3.39E8	±	7.56E7	4.08E7	±	1.11E7	**1.10E7**	**±**	**5.05E6**	9.25E6	±	5.37E6	9.19E6	±	5.49E6
K_2		2.20E8	±	1.37E9	7.73E7	±	1.52E7	6.76E6	±	1.40E6	**533405**	**±**	**100096**	48457	±	8386	4790	±	820
cooperativity		0.0036	±	0.02208	0.01	±	0	0.1	±	0	**1**	**±**	**0**	10	±	0	100	±	0
purityfactor1		0.90	±	0.06	0.91	±	0.06	0.93	±	0.07	**0.97**	**±**	**0.08**	0.99	±	0.08	1.00	±	0.08
purityfactor2		1.01	±	0.04	1.01	±	0.04	0.98	±	0.05	**0.94**	**±**	**0.04**	0.91	±	0.04	0.91	±	0.04
purityfactor3		0.99	±	0.03	0.99	±	0.03	0.97	±	0.03	**0.92**	**±**	**0.03**	0.90	±	0.03	0.90	±	0.03
response1		1.13E11	±	8.49E9	1.12E11	±	8.73E9	1.09E11	±	9.96E9	**1.03E11**	**±**	**9.88E9**	9.94E10	±	9.09E9	9.88E10	±	8.96E9
response2		9.22E10	±	4.85E9	9.26E10	±	4.81E9	9.50E10	±	5.30E9	**9.99E10**	**±**	**5.95E9**	1.02E11	±	6.16E9	1.02E11	±	6.19E9
settingscorrection		1.63	±	0.10	1.64	±	0.1	1.69	±	0.12	**1.79**	**±**	**0.13**	1.85	±	0.13	1.86	±	0.13
reduced χ^2		3.526E9			3.504E9			3.569E9			**3.730E9**			3.798E9			3.807E9		

18. Figure S17

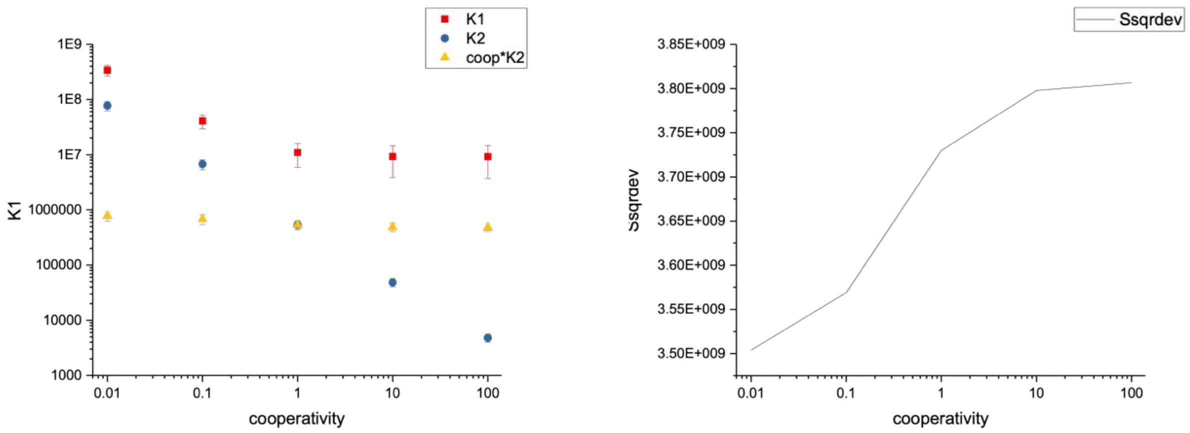

Figure S17 Plot of K_1, K_2 and of the product cooperativity*K_2 as a function of the value of the value of the cooperativity parameter, for binding of Tb^{3+} to LS2-1,5 (Left) and variation of Σdev^2 as a function of cooperativity factor (right). Two regimes are apparent, one at cooperativity below 1 (anticooperativity) and one at cooperativity above 1 but variation in Σdev^2 is too small to allow identification of an optimal cooperativity factor that is not equal to 1.

19. Figure S18

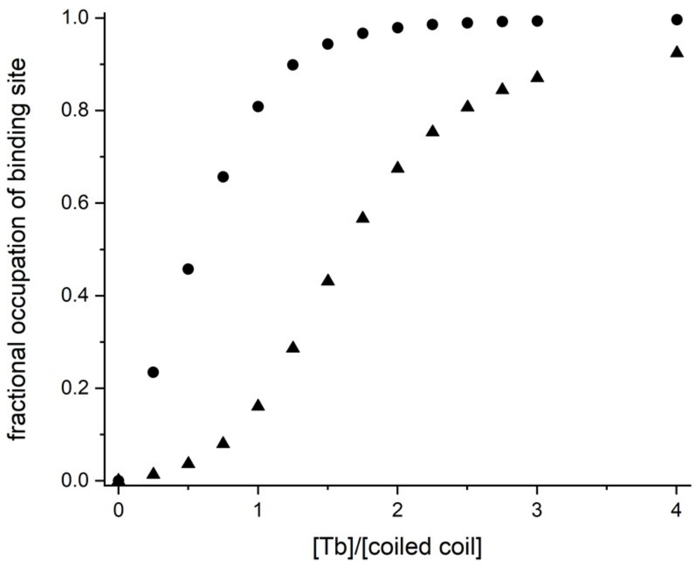

Figure S18. Speciation plot showing predicted fractional Tb^{3+} occupation of binding site 1 (circles) and binding site 2 (triangles) in LS2-1,5, based on binding affinity of 1.1×10^{7} M^{-1} for site 1 and 5×10^{5} M^{-1} for site 2, respectively, with no evidence for cooperativity in binding.

20. Figure S19

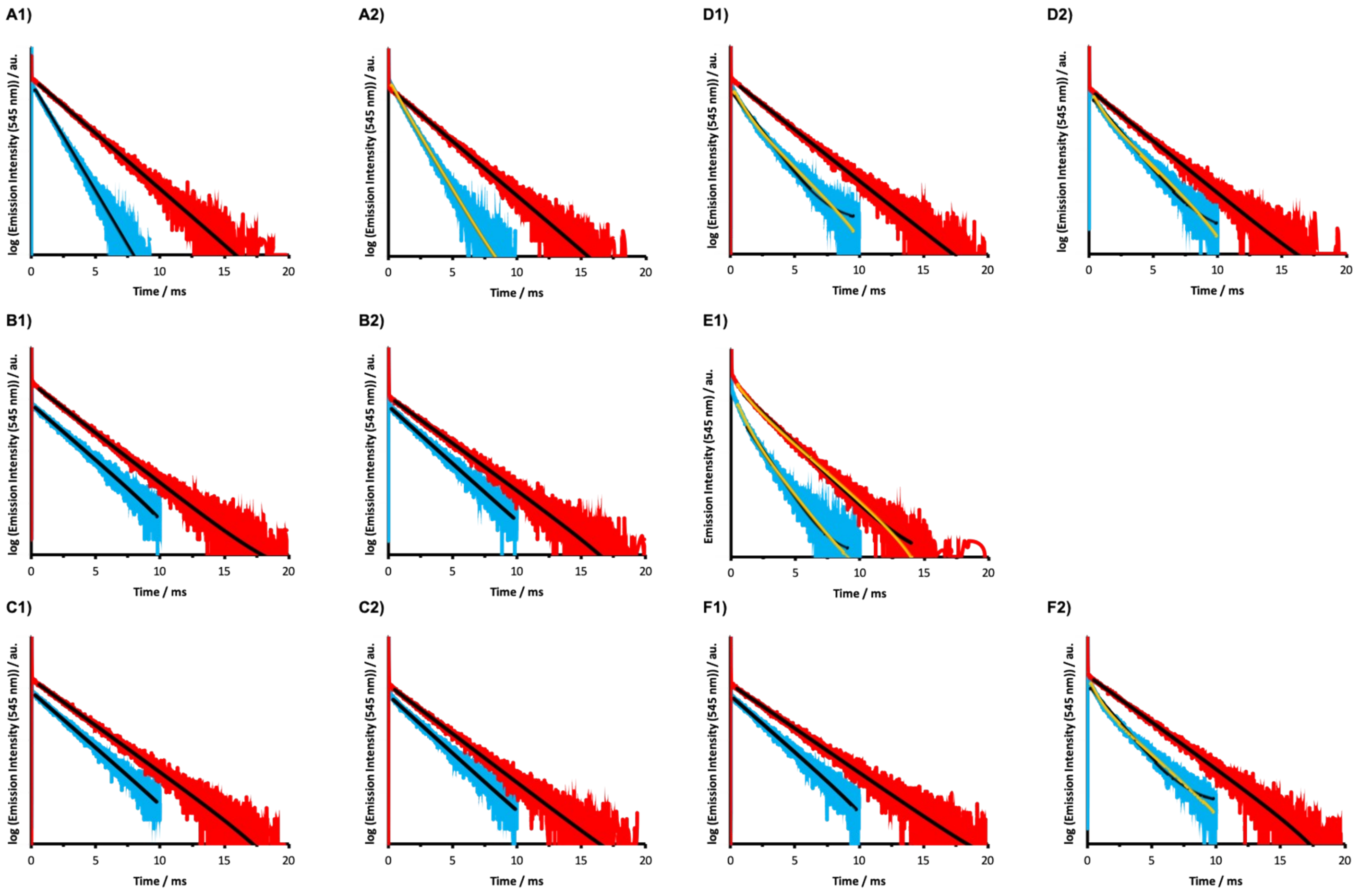

Figure S19. Representative decay profiles for Tb^{3+} emission at 545 nm, recorded in H_2O (blue) and D_2O (red), for 100 µM peptide monomer solutions of A) LS2-1,2, B) LS2-1,3, C) LS2-1,4, D) LS2-1,5, E) LS3-1,6 and F) LS2'-2,3 with 1) 0.3 equivalents Tb^{3+} and 2) 1 equivalent Yb^{3+} and 0.3 equivalents Tb^{3+}, per peptide trimer in 10 mM HEPES buffer pH 7.0. Lifetimes are fitted using mono-exponential decays (black line) and bi-exponential decays (orange lines).

21. Figure S20

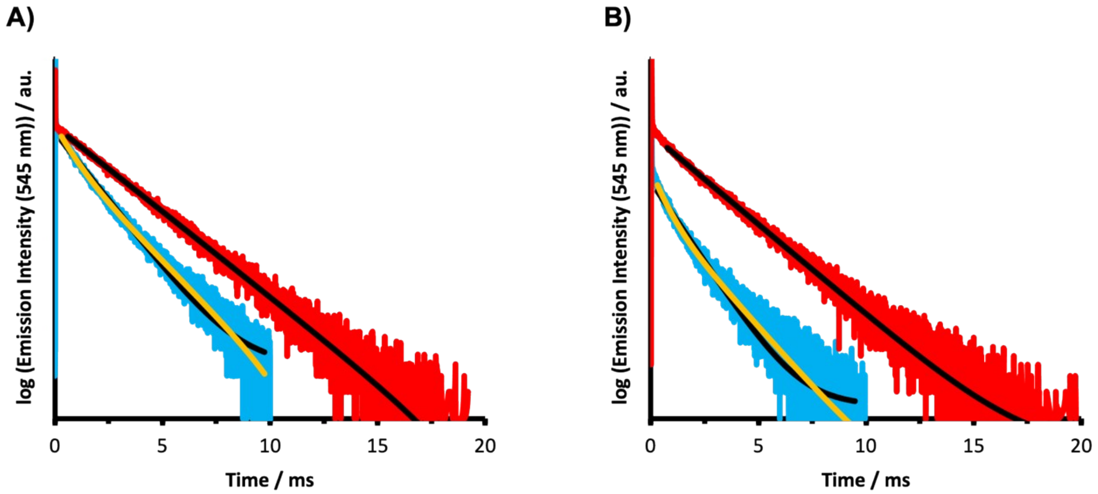

Figure S20. Representative decay profiles for Tb^{3+} emission at 545 nm, recorded in H_2O (blue) and D_2O (red), in the presence of 0.3 equivalents Tb^{3+} per peptide trimer for 100 μM peptide monomer solutions of A) LS2-1,5(7Q) and B) LS2-1,5(35Q), in 10 mM HEPES buffer pH 7.0. Lifetimes are fitted using mono-exponential decays (black line) and bi-exponential decays (orange lines).

22. Table S3

Table S3 Number of water molecules coordinated to peptide bound Tb^{3+}.

**Peptide**	**Equivalents Ln^{3+} per Trimer**		**Number of Coordinated Waters to Tb^{3+}**
	**Tb^{3+}**	**Yb^{3+}**	
LS2-1,2	0.3	0	2.0 ± 0.1
LS2-1,3	0.3	0	0.1 ± 0.1
LS2-1,4	0.3	0	0.1 ± 0.1
LS2-1,5	0.3	0	0.2 ± 0.9 (70%)
LS3-1,6	0.3	0	0.8 ± 0.1 (74%)
LS2’-2,3	0.3	0	0.2 ± 0.1
LS2-1,2	0.3	1	2.0 ± 0.4 (82%)
LS2-1,3	0.3	1	0.1 ± 0.2
LS2-1,4	0.3	1	0.0 ± 0.2 (90%)
LS2-1,5	0.3	1	0.3 ± 0.1 (80%)
LS2’-2,3	0.3	1	0.2 ± 0.1
LS2-1,5(7Q)	0.3	0	0.4 ± 0.1 (72%)
LS2-1,5(35Q)	0.3	0	0.3 ± 0.2 (75%)

Number of coordinated waters determined by luminescence emission of 10 μM Tb^{3+} in the presence of 100 μM peptide monomer solutions in 10 mM HEPES buffer pH 7.0, where the error represents the standard deviation, based on three repeats.

23. Figure S21 – Gd binding CD data

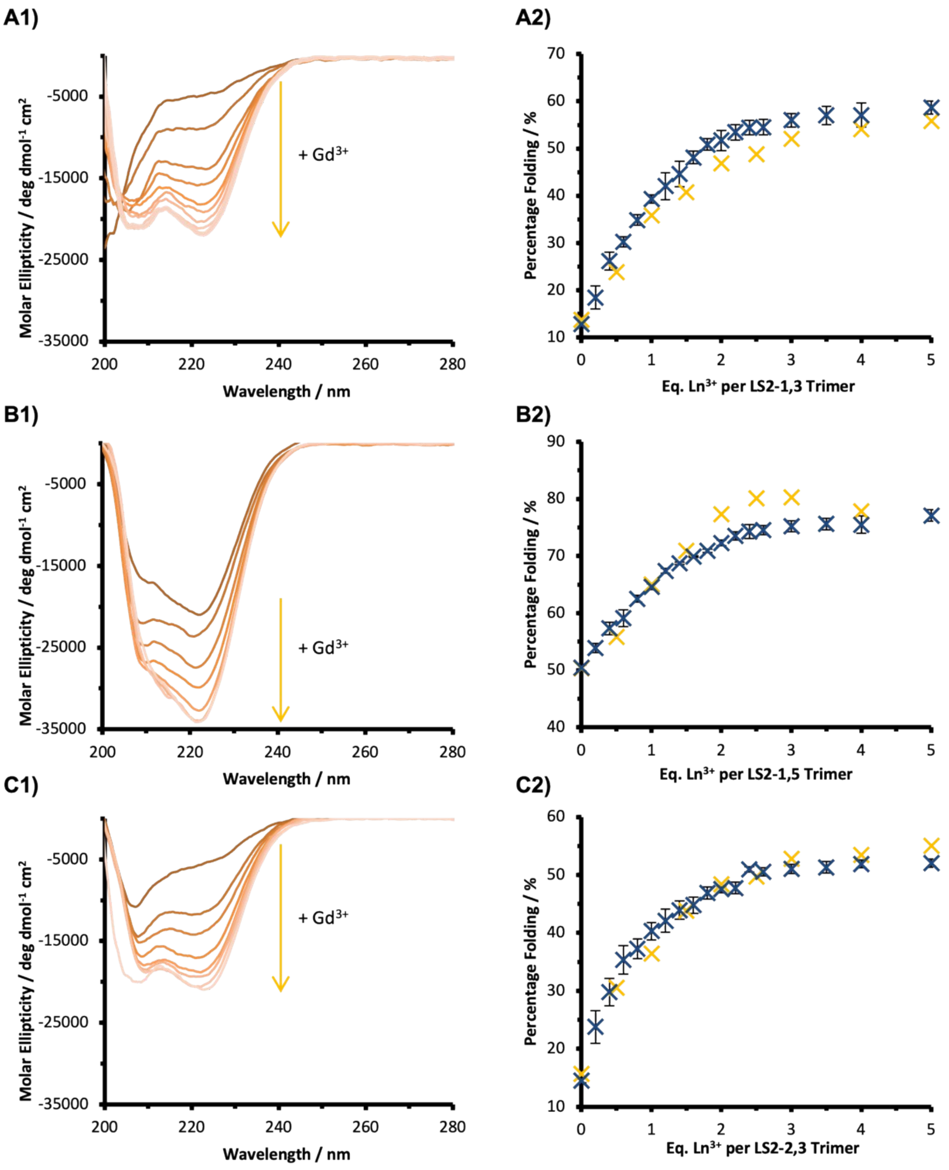

Figure S21. 1) Ln^{3+} binding titrations monitored by CD and 2) plots of percentage folded, based on molar ellipticity at 222 nm, as a function of Ln^{3+} equivalents per trimer (Gd^{3+} orange and Tb^{3+} dark blue), with 30 μM A) LS2-1,3, B) LS2-1,5 and C) LS2'-2,3 monomer solution in 10 mM HEPES buffer pH 7.0. Data for the Tb^{+} titrations are the average of three repeats where the error bars represent the standard deviation.

24. Figure S22

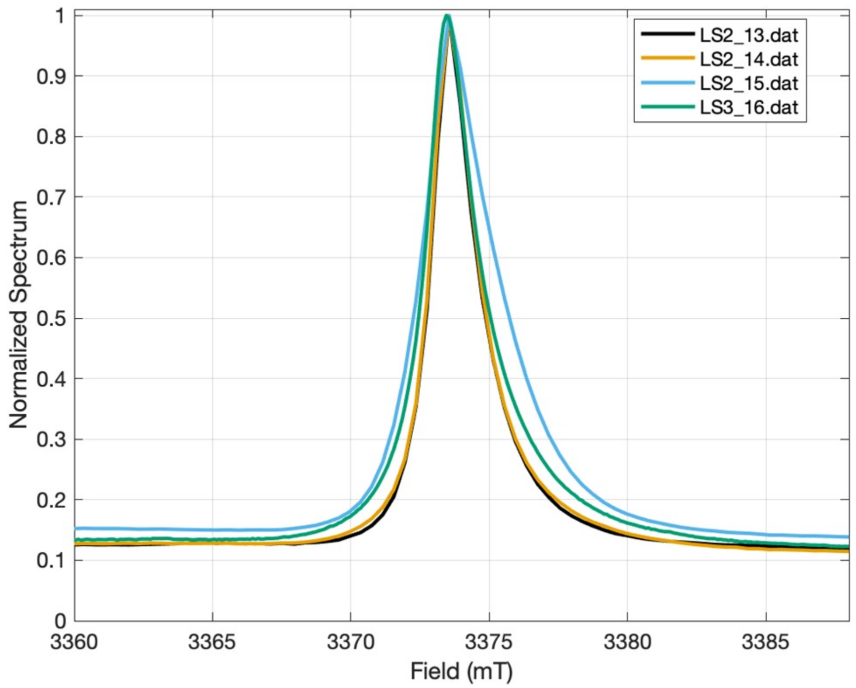

Figure S22. ED-FS spectra (focussing on the central transition) of the peptides at W-band. The full-width at half-height (FWHH) measurement ranges from approximately 2.2 mT for LS2-1,3 to 3.5 mT for LS2-1,5, with LS2-1,3 ≅ LS2-1,4 < LS3-1,6 < LS2-1,5. The peptides where the Gd^{3+} are at the two termini appear to have a slightly broader spectra than the LS2-1,3 and LS2-1,4 peptides.

25. Figure S23

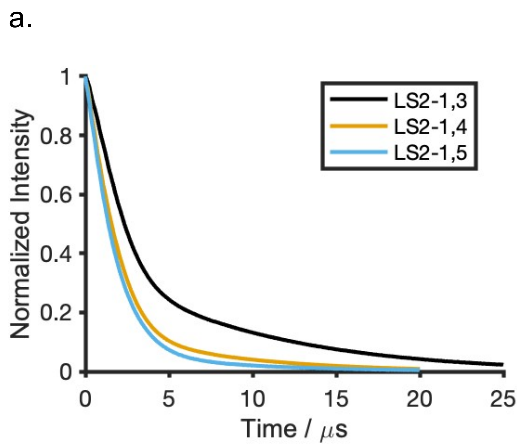

Figure S23. Echo decay curves for the LS2 peptides: a. measured at the field corresponding to the maximum intensity of the central transition (CT); b. measured at an offset (approx. 11 mT) from the maximum of the CT.

26. Figure S24

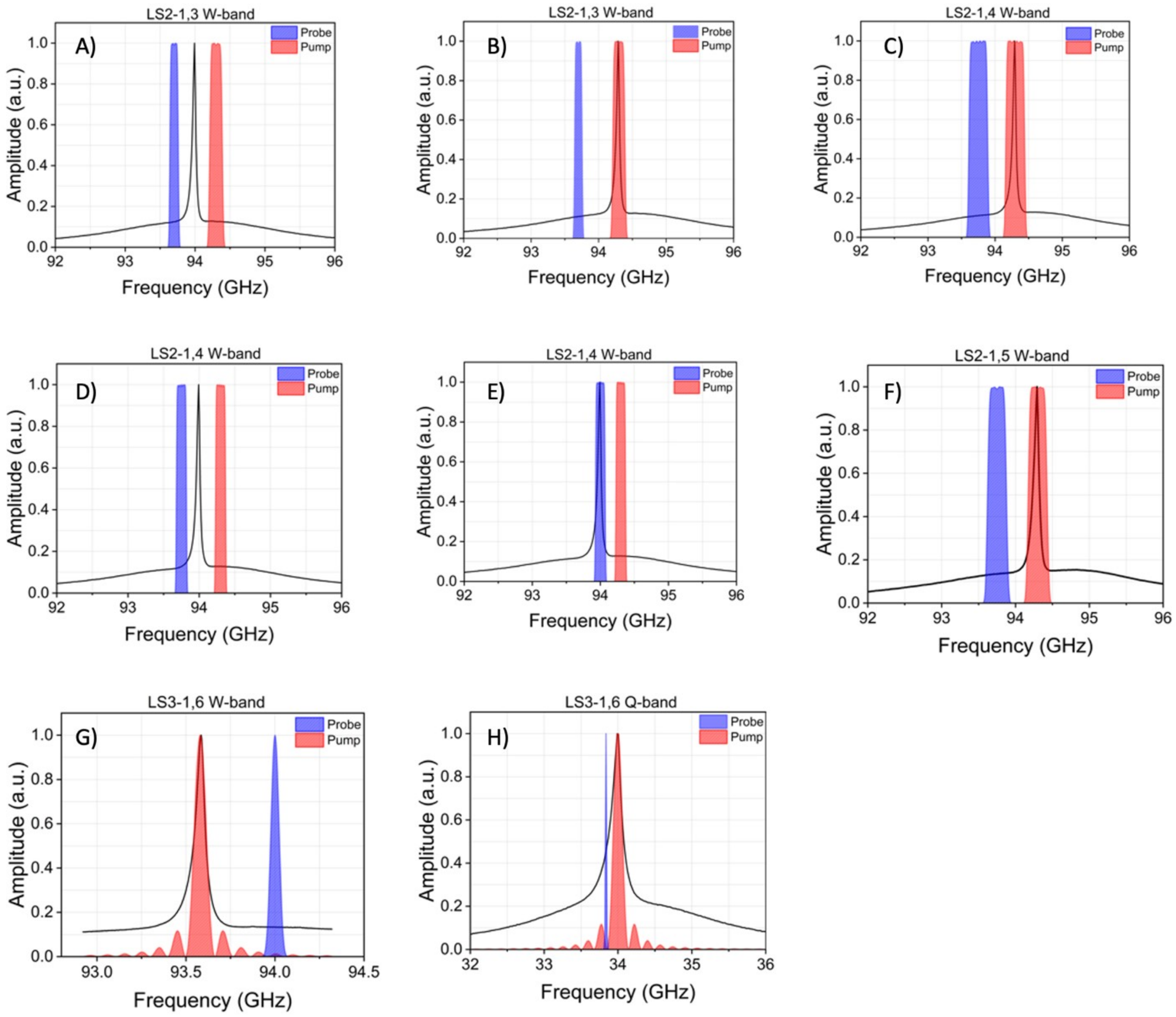

Figure S24. ED-FS showing the excitation bandwidth and frequency of the pump pulse and observer sequence ("probe") in the DEER experiments. Panels a, d, f and g correspond to the DEER data shown in the main paper (Figure 4). A) LS2-1,3 pump and probe avoid the CT; B) LS2-1,3 pump on CT; C) LS2-1,4 pump on CT; D) LS2-1,4 pump and probe avoid the CT; E) LS2-1,4 probe on CT; F) LS2-1,5 pump on CT; G) LS3-1,6 pump on CT; H) LS3-1,6 pump on CT, measurement at Q-band.

27. Table S4 LS2-1,3 W-band DEER parameters. “Avoid CT” is the data used in the main paper.

**LS2-1,3**	**Pump on CT**	**Avoid CT**
Number of shots per point	6 k	6 k
Number of scans used in average	26	145
Shot repetition	3 kHz	3 kHz
Pump pulse	40 ns HS4 94.3 GHz	40 ns HS4 94.3 GHz
Observer pulses	60 ns HS4 93.7 GHz	60 ns HS4 93.7 GHz
τ_1	140 ns	140 ns
τ_2	1690 ns	1690 ns
Time step	10 ns	10 ns
Modulation depth	1.5%	0.6%
Zero time	143 ns	140 ns
Background	384 ns	192 ns
Tikhonov regularization parameter	50.1	7.9

28. Table S5 LS2-1,4 W-band DEER parameters. "Avoid CT" is the data used in the main paper.

**LS2-1,4**	**Pump on CT**	**Avoid CT**	**Observe CT**
Number of shots per point	10 k	10 k	10 k
Number of scans used in average	105	141	95
Shot repetition	4 kHz	4 kHz	4 kHz
Pump pulse	40 ns HS6 94.3 GHz	80 ns HS6 94.3 GHz	80 ns HS6 94.3 GHz
Observer pulses	40 ns HS6 93.75 GHz	80 ns HS6 93.7 GHz	80 ns HS6 94 GHz
τ_1	160 ns	220 ns	220 ns
τ_2	4710 ns	4120 ns	4120 ns
Time step	20 ns	20 ns	20 ns
Modulation depth	2.4%	1.1%	0.6%
Zero time	154 ns	201 ns	209 ns
Background	320 ns	620 ns	560 ns
Tikhonov regularization parameter	25.1	31.6	12.6

29. Table S6 LS2-1,5 W-band DEER parameters.

**LS2-1,5**	**W-band**
Number of shots per point	6 k
Number of scans used in average	77
Shot repetition	4 kHz
Pump pulse	28 ns HS4<br>94.3 GHz
Observer pulses	28 ns HS4<br>93.7 GHz
τ_1	172 ns
τ_2	5522 ns
Time step	10 ns
Modulation depth	1.9%
Zero time	169 ns
Background	2480 ns
Tikhonov regularization parameter	316.2

30. Table S7 LS3-1,6 DEER parameters at W- and Q-band. The Q-band result is the data used in the main paper.

**LS3-1,6**	**W-band**	**Q-band**
Number of shots per point	3 k	30
Number of scans used in average	38	793
Shot repetition	1 kHz	300 ms
Pump pulse	10.5 ns rectangular 93.58 GHz	6 ns rectangular 34.013 GHz ($\Delta\nu$= 165 MHz)
Observer pulses	5 and 10 ns rectangular 94 GHz	32 ns rectangular 33.848 GHz
τ_1	298 ns	200 ns
τ_2	6640 ns	6000 ns
Time step	20 ns	8 ns
Modulation depth	1.2%	0.7%
Zero time	208 ns	164 ns
Background	2820 ns	3088 ns
Tikhonov regularization parameter	199.5	2511.9

31. Table S8 DeerLab results tables for Gaussian fitting in DeerLab. Top four are with standard deviation 0.2 nm and bottom four are for default fitting values. Each of the four sets are in order LS2-1,3, LS2-1,4, LS2-1,5 and LS3-1,6.

Goodness-of-fit:

Dataset	Noise level	Reduced χ2	Residual autocorr.	RMSD
#1	0.000	1.000	0.000	0.000

Model parameters:

Parameter	Value	95%-Confidence interval	Unit	Description
mod	0.006	(0.006,0.006)		Modulation depth
reftime	0.150	(0.149,0.151)	μs	Refocusing time
conc	326.680	(305.772,347.588)	μM	Spin concentration
mean	2.150	(2.129,2.172)	nm	Mean
std	0.200	(0.169,0.200)	nm	Standard deviation
scale	1.000	(1.000,1.000)	None	Overall echo amplitude/scale

Goodness-of-fit:

Dataset	Noise level	Reduced χ2	Residual autocorr.	RMSD
#1	0.000	3.226	1.358	0.001

Model parameters:

Parameter	Value	95%-Confidence interval	Unit	Description
mod	0.009	(0.009,0.010)		Modulation depth
reftime	0.221	(0.217,0.226)	μs	Refocusing time
conc	104.231	(90.409,118.054)	μM	Spin concentration
mean	3.087	(3.045,3.130)	nm	Mean
std	0.200	(0.152,0.200)	nm	Standard deviation
scale	0.998	(0.998,0.998)	None	Overall echo amplitude/scale

Goodness-of-fit:

Dataset	Noise level	Reduced $\chi2$	Residual autocorr.	RMSD
#1	0.001	2.761	1.385	0.001

Model parameters:

Parameter	Value	95%-Confidence interval	Unit	Description
mod	0.016	(0.015,0.017)		Modulation depth
reftime	0.183	(0.172,0.195)	µs	Refocusing time
conc	307.223	(288.416,326.029)	µM	Spin concentration
mean	4.249	(4.207,4.290)	nm	Mean
std	0.200	(0.158,0.200)	nm	Standard deviation
scale	0.997	(0.997,0.997)	None	Overall echo amplitude/scale

Goodness-of-fit:

Dataset	Noise level	Reduced $\chi2$	Residual autocorr.	RMSD
#1	0.001	1.000	0.000	0.000

Model parameters:

Parameter	Value	95%-Confidence interval	Unit	Description
mod	0.010	(0.009,0.010)		Modulation depth
reftime	0.345	(0.308,0.345)	µs	Refocusing time
conc	0.010	(0.010,7.164)	µM	Spin concentration
mean	5.107	(5.063,5.151)	nm	Mean
std	0.200	(0.166,0.200)	nm	Standard deviation
scale	0.999	(0.999,0.999)	None	Overall echo amplitude/scale

Goodness-of-fit:

Dataset	Noise level	Reduced $\chi2$	Residual autocorr.	RMSD
#1	0.000	1.000	0.000	0.000

Model parameters:

Parameter	Value	95%-Confidence interval	Unit	Description
mod	0.006	(0.006,0.006)		Modulation depth
reftime	0.150	(0.149,0.151)	µs	Refocusing time
conc	298.600	(278.284,318.916)	µM	Spin concentration
mean	2.143	(2.120,2.165)	nm	Mean
std	0.292	(0.260,0.323)	nm	Standard deviation
scale	1.000	(1.000,1.000)	None	Overall echo amplitude/scale

Goodness-of-fit:

Dataset	Noise level	Reduced χ2	Residual autocorr.	RMSD
#1	0.001	1.108	0.480	0.001

Model parameters:

Parameter	Value	95%-Confidence interval	Unit	Description
mod	0.018	(0.018,0.019)		Modulation depth
reftime	0.184	(0.177,0.190)	μs	Refocusing time
conc	253.294	(242.269,264.319)	μM	Spin concentration
mean	4.233	(4.179,4.287)	nm	Mean
std	0.704	(0.645,0.763)	nm	Standard deviation
scale	0.999	(0.999,0.999)	None	Overall echo amplitude/scale

Goodness-of-fit:

Dataset	Noise level	Reduced χ2	Residual autocorr.	RMSD
#1	0.001	1.000	0.000	0.000

Model parameters:

Parameter	Value	95%-Confidence interval	Unit	Description
mod	0.010	(0.009,0.010)		Modulation depth
reftime	0.345	(0.308,0.345)	μs	Refocusing time
conc	0.010	(0.010,7.171)	μM	Spin concentration
mean	5.107	(5.062,5.152)	nm	Mean
std	0.203	(0.169,0.236)	nm	Standard deviation
scale	0.999	(0.999,0.999)	None	Overall echo amplitude/scale

32. Figure S25

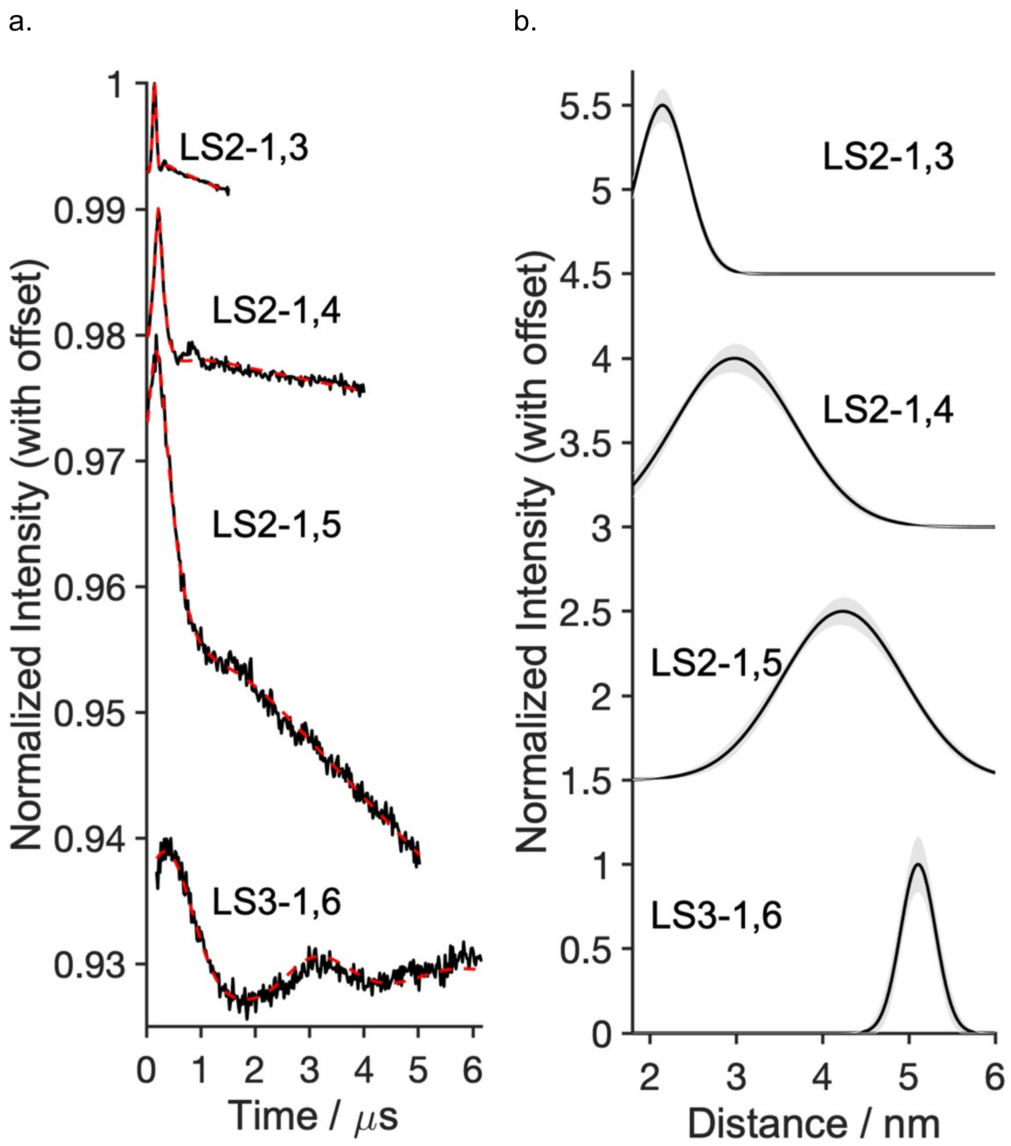

Figure S25. Main paper DEER analysed by DeerLab using Gaussian fitting without a constraint on the standard deviation of the Gaussian. a) time traces (black) and simulated fits (red dash); b) distance distributions with 95% confidence shown in grey.

33. Figure S26

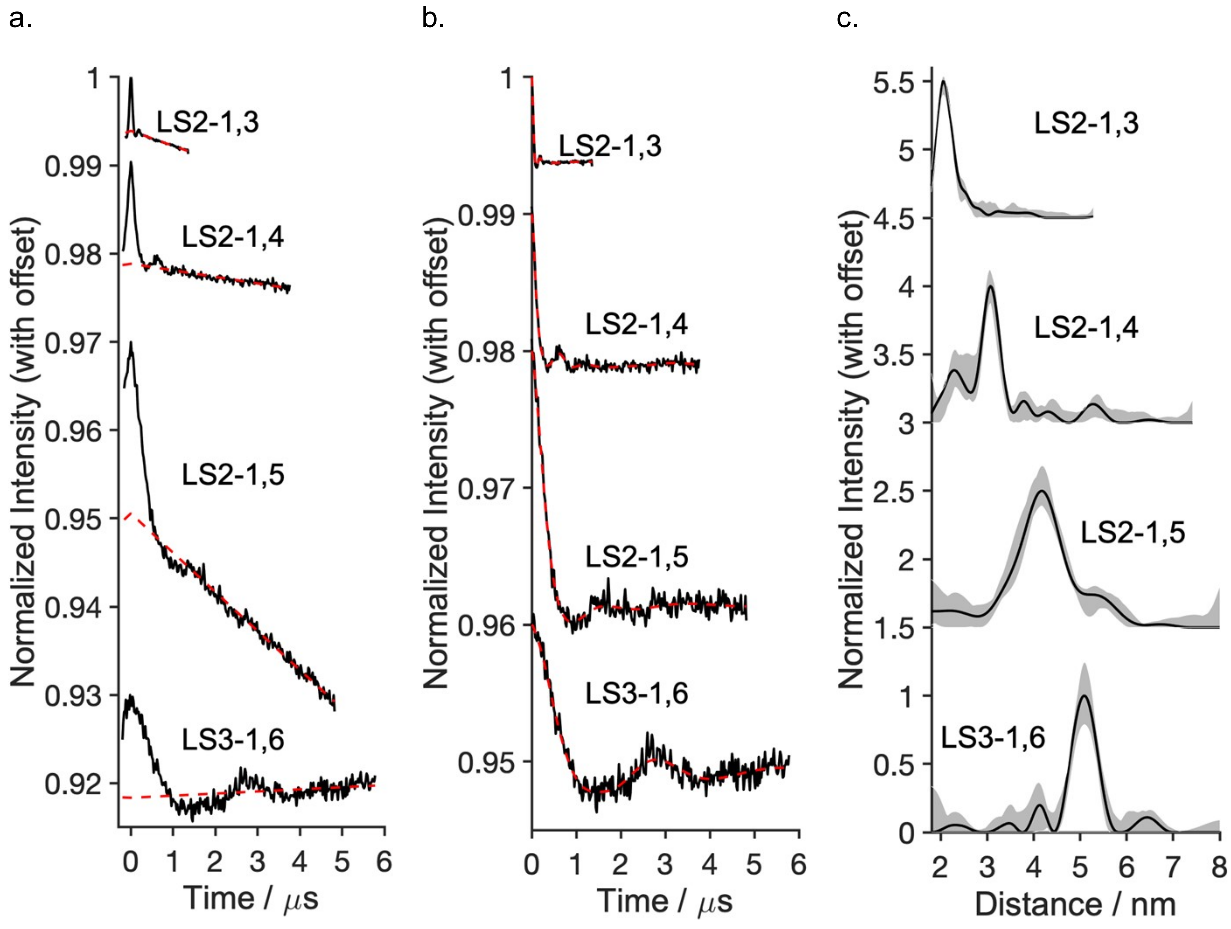

Figure S26. Main paper DEER results from DeerAnalysis using Tikhonov regularization. a) time traces (black) and background fits (red dash); b) time trace following background subtraction with simulated fits (red dash); c) distance distributions with uncertainty determined by Validation in grey.

34. Figure S27

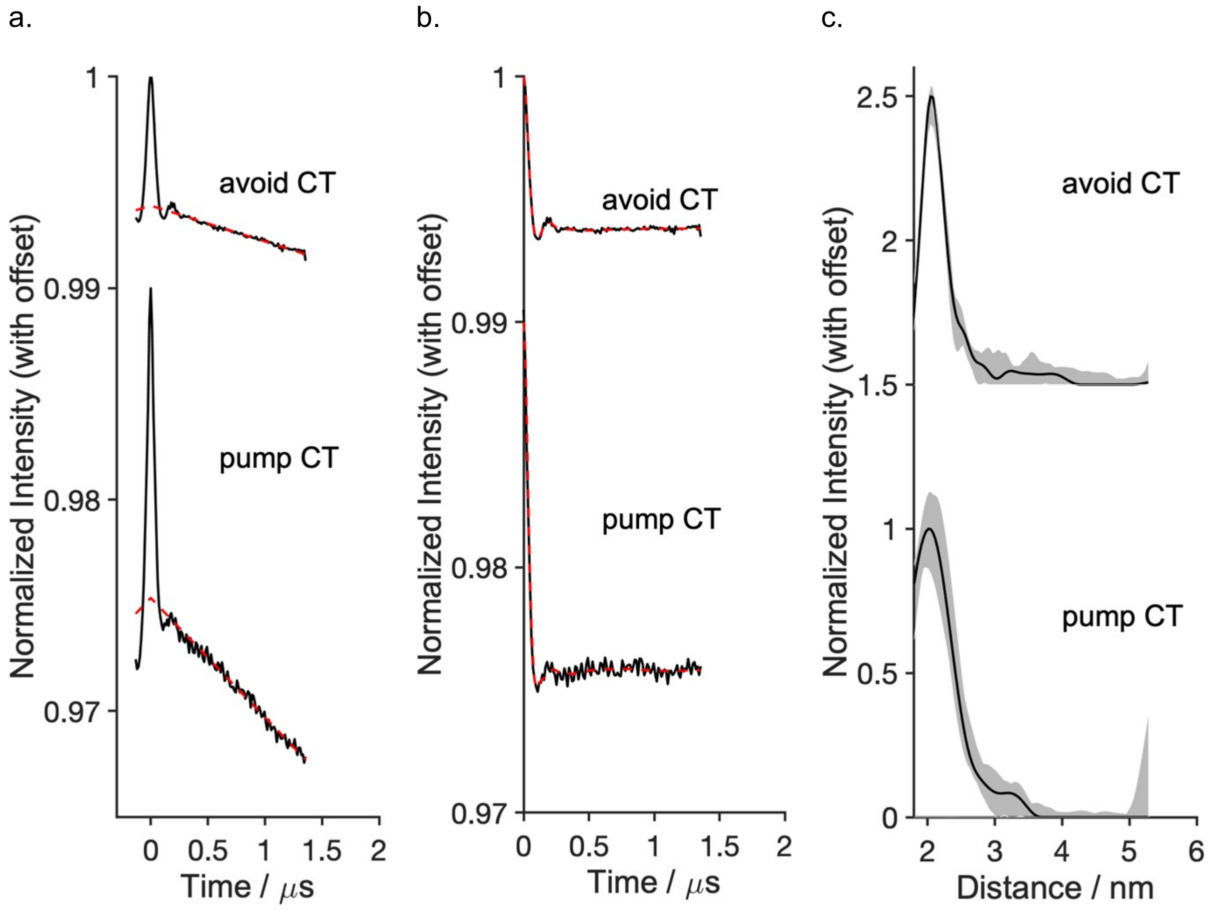

Figure S27. LS2-1,3 DEER with pumping on and off the CT and corresponding distance distributions extracted using Tikhonov regularization. a) time traces (black) and background fits (red dash); b) time trace following background subtraction with simulated fits (red dash); c) distance distributions with uncertainty determined by Validation in grey.

35. Figure S28

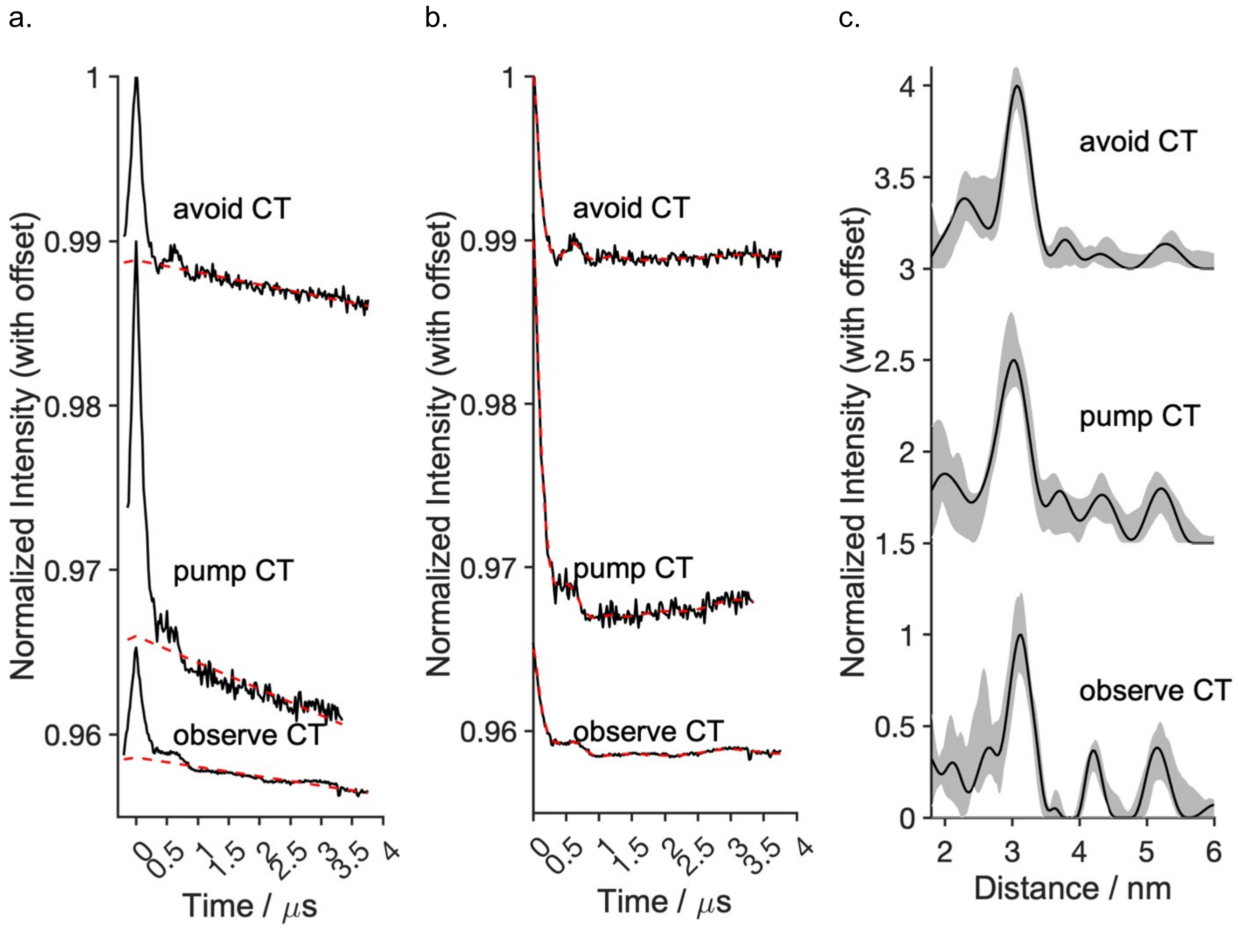

Figure S28. LS2-1,4 DEER with pumping on and off the CT and corresponding distance distributions extracted using Tikhonov regularization. a) time traces (black) and background fits (red dash); b) time trace following background subtraction with simulated fits (red dash); c) distance distributions with uncertainty determined by Validation in grey.

36. Figure S29

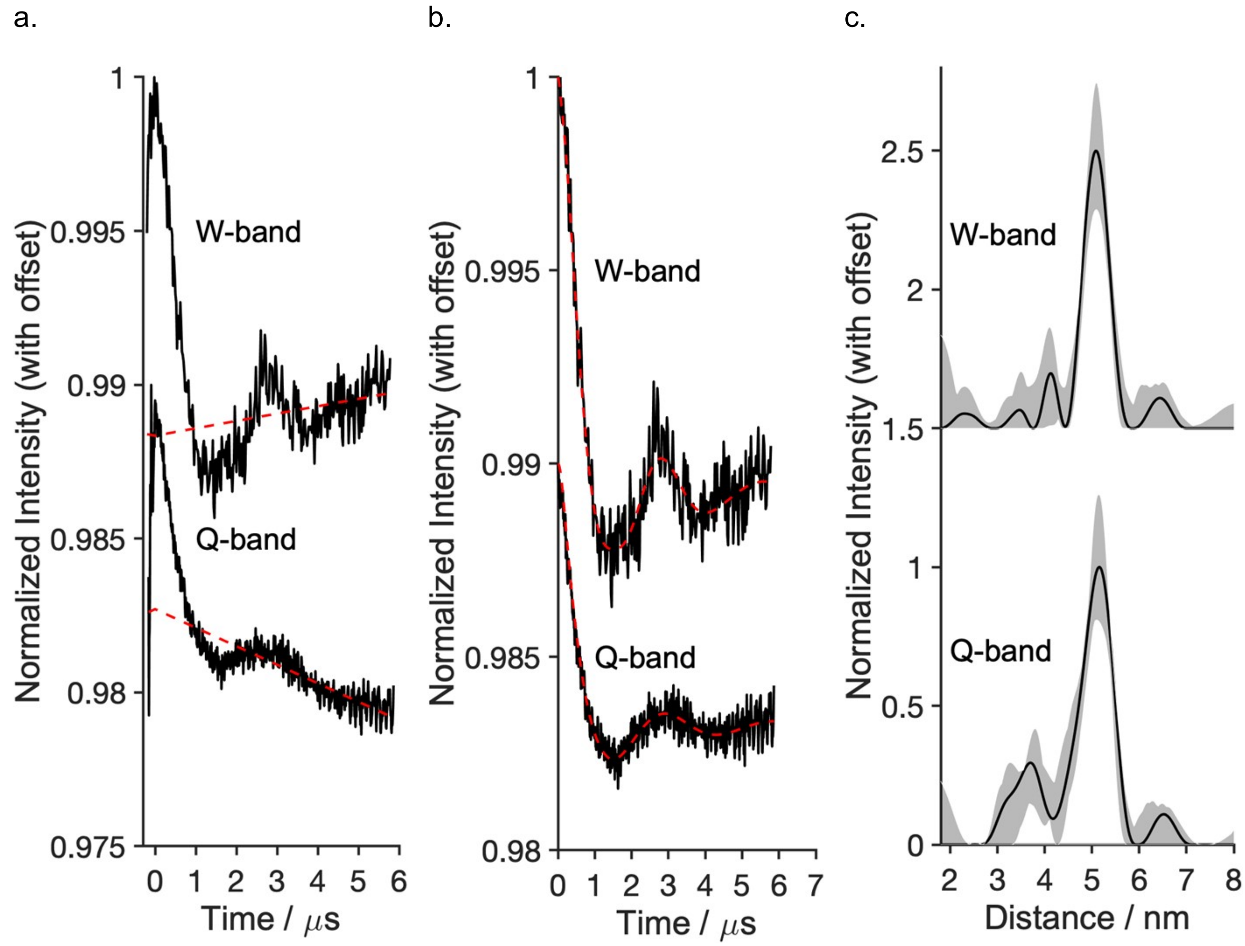

Figure S29. LS3-1,6 DEER with pumping on and off the CT and corresponding distance distributions extracted using Tikhonov regularization. a) time traces (black) and background fits (red dash); b) time trace following background subtraction with simulated fits (red dash); c) distance distributions with uncertainty determined by Validation in grey.

37. Table S9 X-ray data collection and refinement statistics.

	**xLS2"-2,3**
Wavelength	1.4001 Å
Resolution range	23.79 - 2.76 (2.99 - 2.76)
Space group	P 6
Unit cell	38.74 38.74 67.46 90 90 120
Total reflections	30605 (6448)
Unique reflections	1507 (315)
Multiplicity	20.3 (20.5)
Completeness (%)	97.8 (100)
Mean I/sigma(I)	29.6 (5.4)
Wilson B-factor	95.24
R_{merge}	0.044 (0.414)
R_{meas}	0.047 (0.435)
R_{pim}	0.015 (0.135)
$CC_{1/2}$	0.999 (0.998)
Anomalous completeness	99.8 (100)
Anomalous multiplicity	10.4 (10.4)
DelAnom correlation between half-sets	0.767 (0.45)
Reflections used in refinement	1473 (139)
Reflections used for R_{free}	74 (4)
R_{work}	0.2253 (0.3156)
R_{free}	0.2315 (0.3990)
Number of non-hydrogen atoms	363
macromolecules	359
ligands	4
solvent	0
Protein residues	45
RMS(bonds)	0.004
RMS(angles)	0.65
Ramachandran favored (%)	97.67
Ramachandran allowed (%)	2.33
Ramachandran outliers (%)	0.00

Rotamer outliers (%)	2.78
Clashscore	1.36
Average B-factor	123.34
macromolecules	123.30
ligands	126.92
Number of TLS groups	1
PDB code	9SOM

Statistics for the highest-resolution shell are shown in parentheses.

38. Figure S30

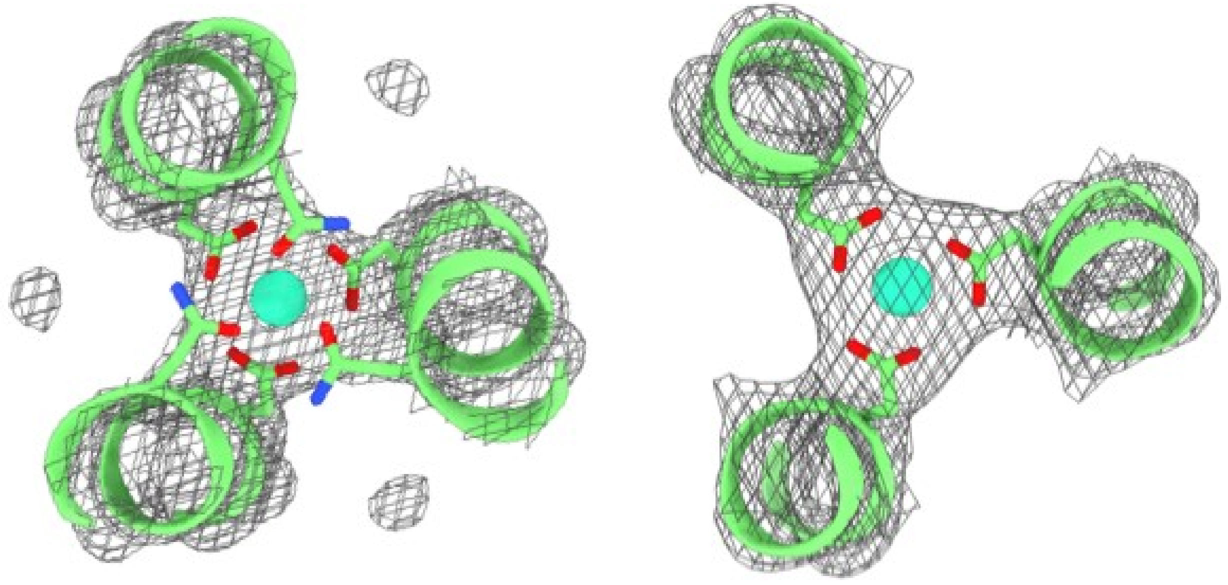

Figure S30. Close-up views of the crystal structure of Tb^{3+} bound at the (left) Asn_3Asp_3 and (right) Asp_3 binding sites within the interior of the parallel three-stranded coiled coil generated by xLS2”-2,3. Top down view from the N-terminus, showing main-chain atoms as ribbons, the binding-site Asn and Asp side chains in stick form (C green, O red, N blue), the bound Tb^{3+} ions as cyan spheres, and the electron density map (grey mesh, 1.5 σ) overlaid.

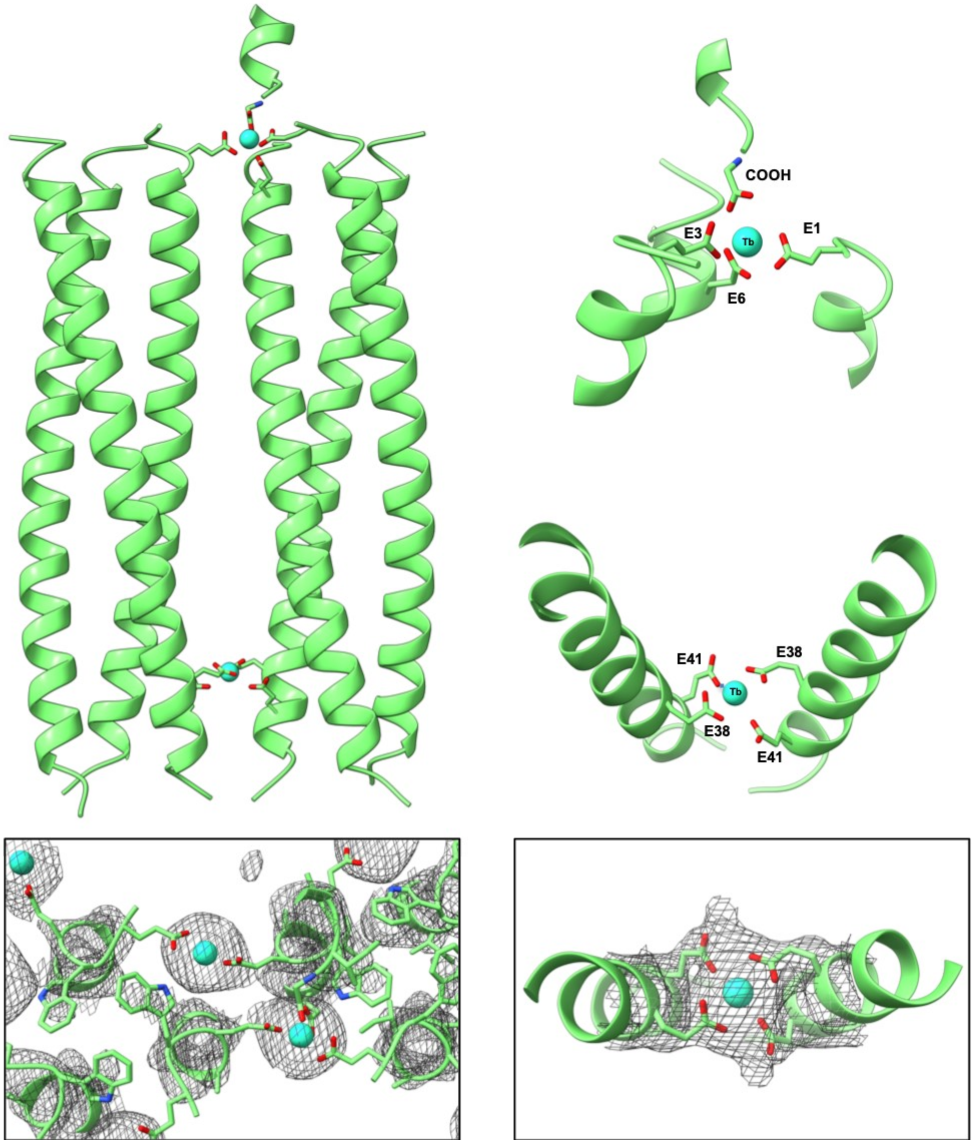

39. Figure S31

Figure S31. X-ray crystal structure of the parallel three-stranded coiled coil highlighting external Tb^{3+} binding sites that facilitate crystal packing. (Top left) Full coiled-coil trimers with Tb^{3+} ions (cyan spheres) coordinated at both ends of the bundle. (Top right and middle right) Close-up views of two representative external Tb^{3+} binding motifs formed by glutamate residues at inter-helical interfaces. (Bottom left and right) Electron density maps (grey mesh, 1.5 σ) confirm Tb^{3+}

positions and coordination environments. Tb^{3+}-mediated interactions between helices contribute to lattice contacts and stabilize the crystal architecture.

40. Figure S32

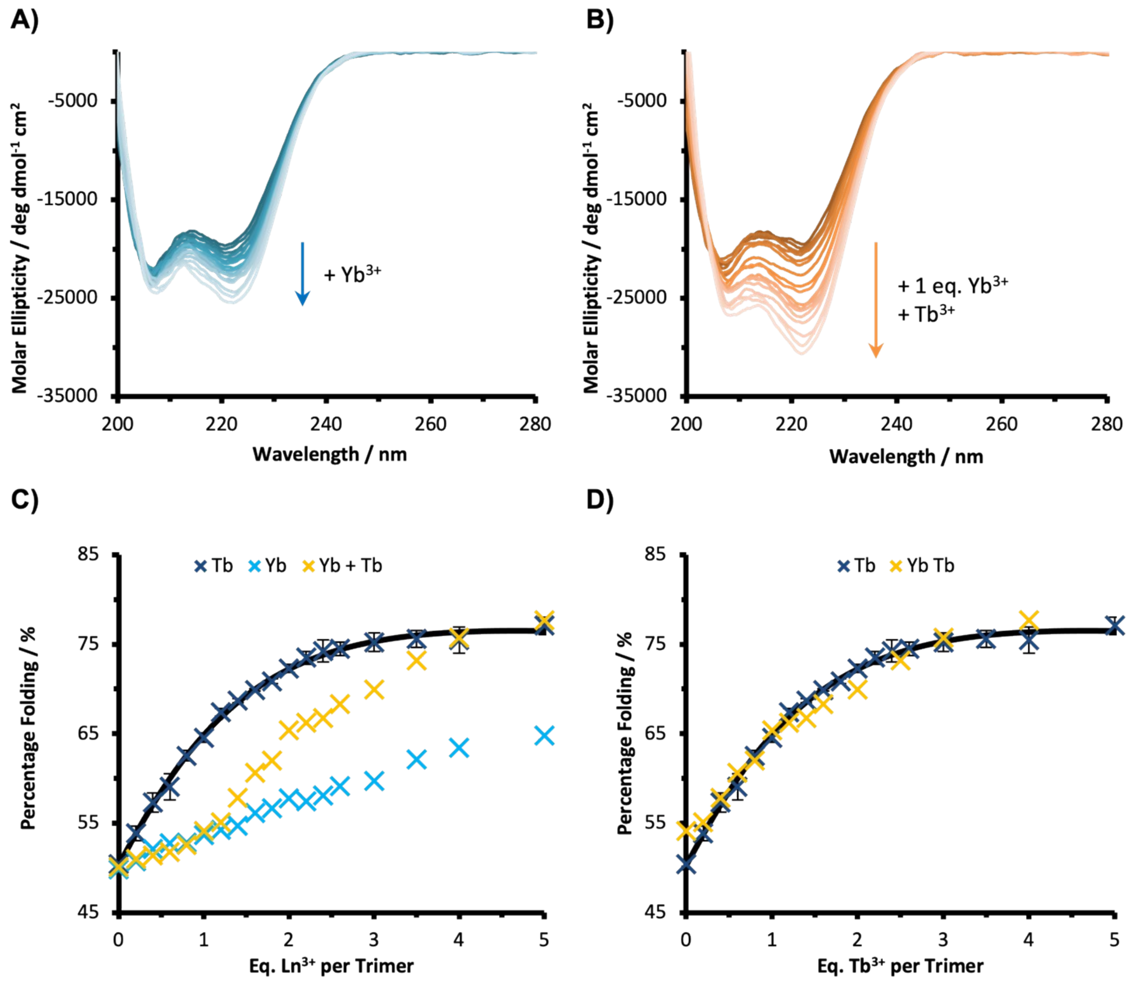

Figure S32. Ln^{3+} LS2-1,5 binding titrations monitored by CD where A) Yb^{3+} (light blue) and B) 1 equivalent Yb^{3+} followed by titration of Tb^{3+} (orange) into a 30 µM LS2-1,5 monomer solution in 10 mM HEPES buffer pH 7.0. C) Plot of percentage folding, based on molar ellipticity at 222 nm, as a function Ln^{3+} equivalents per trimer (dark blue Tb^{3+} only, light blue Yb^{3+} only, and yellow 1 equiv. Yb^{3+} followed by Tb^{3+}). Data for Tb^{3+} is the average of three repeats where error bars represent the standard deviation.

41. Figure

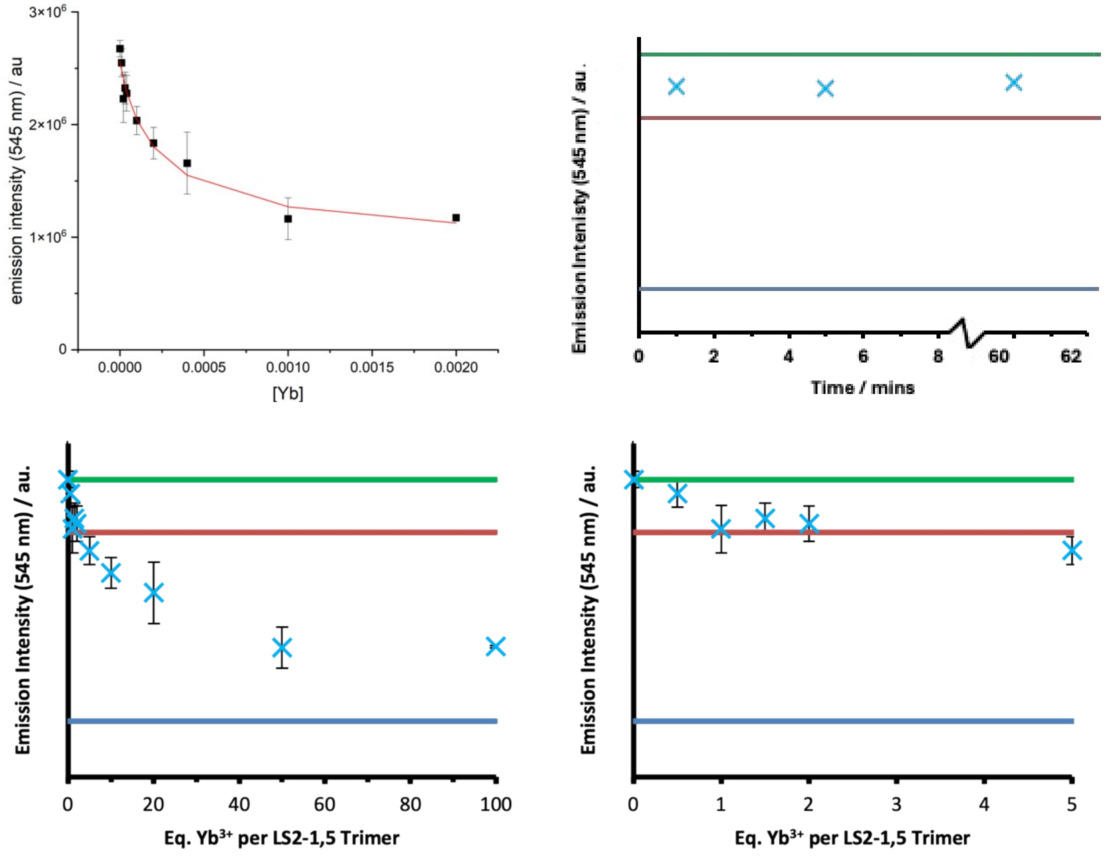

3×10^6
2×10^6
1×10^6
0
emission intensity (545 nm) / au
0.0000
0.0005
0.0010
0.0015
0.0020
[Yb]
Emission Intenisty (545 nm) / au.
0
2
4
6
8
60
62
Time / mins
Emission Intensity (545 nm) / au.
0
20
40
60
80
100
Eq. Yb3+ per LS2-1,5 Trimer
Emission Intensity (545 nm) / au.
0
1
2
3
4
5
Eq. Yb3+ per LS2-1,5 Trimer

Figure S33. Luminescence Tb^{3+} displacement plots for 20 μM Tb^{3+} and 30 μM LS2-1,5 monomer (green line) in the presence of increasing amounts of (Top left) competing Yb^{3+} (0.5, 1, 1.5, 2, 5, 10, 20 50 and 100 equivalents per trimer) and (Top right) a zoom-in displaying only 0-5 equivalents Yb^{3+} added per peptide trimer, in 10 mM HEPES buffer pH 7.0. The blue line represents emission for the apo peptide and the red line 10 μM Tb^{3+} and 30 μM LS2-1,5 monomer. Data is based on the integration of the 545 nm Tb^{3+} emission peak, error bars represent the standard deviation of the three repeats, λ_{ex} = 280 nm. The same data analysed in terms of a competition binding model (Bottom left). Equilibration is reached within the first time point as illustrated upon addition of 1 equiv. Yb^{3+} over time (Bottom right).

42. Figure S34

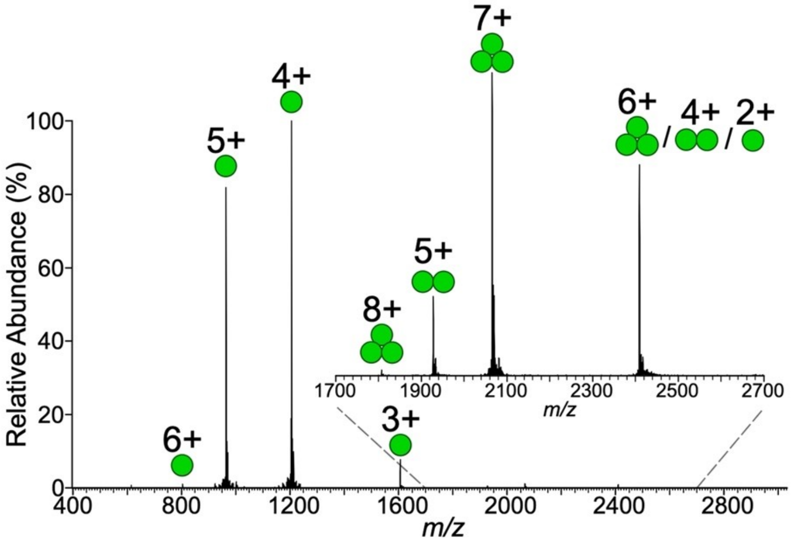

Figure S34. Native mass spectrum of apo LS2-1,5. Peptide monomers, dimers and trimers are indicated with one, two and three green circles, respectively. Note that although dimeric complexes are observed, the trimeric complex has a higher relative abundance confirming the trimer is the preferential complex topology.

43. Figure S35

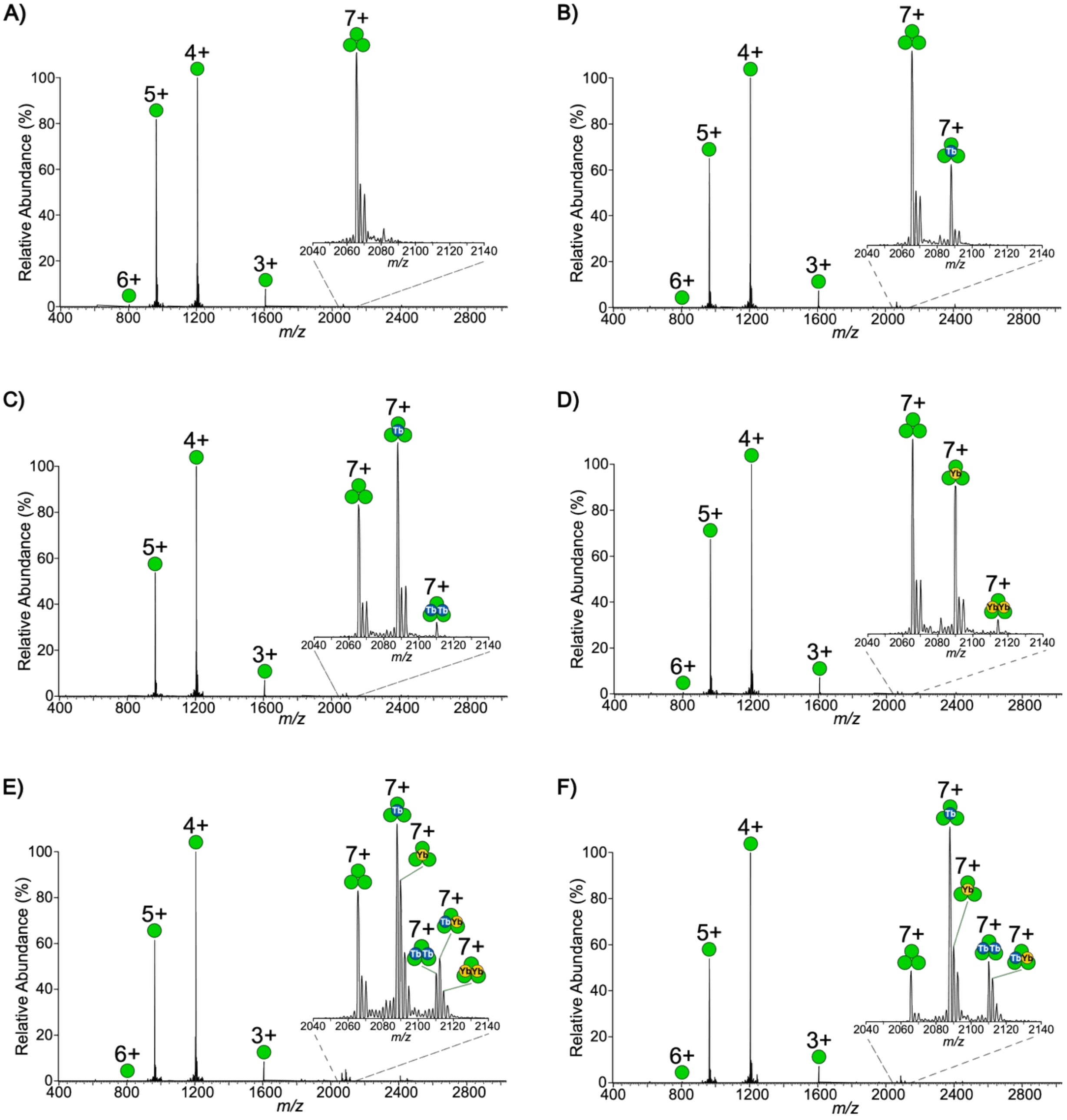

Figure S35. Native mass spectra of LS2-1,5 in the absence (A) and presence of increasing equivalents of Tb/Yb; B) 0.5 eq. Tb^{3+}; C) 1.0 eq. Tb^{3+}; D) 1.0 eq. Yb^{3+}; E) 1.0 eq. Tb^{3+} + 1.0 eq. Yb^{3+}; and F) 2.0 eq. Tb^{3+} + 1.0 eq. Yb^{3+}. The insets highlight the most abundant 7+ charge states. Peptide monomers, Tb and Yb are indicated with green, blue and yellow circles, respectively.

44. Table S10

Table S10. Average theoretical and observed molecular weights of unbound and metal-bound LS2-1,5 complexes, determined using native MS. The error on the masses calculated across charge states in all cases was ≤ 0.05 Da.

Complex	Theoretical Mass (Da)	Observed Mass (Da)
LS2-1,5 (Monomer)	4817.5	4817.6
LS2-1,5 (Dimer)	9635.0	9635.2
LS2-1,5 (Trimer)	14452.5	14452.8
LS2-1,5 (Trimer) + 1 Tb	14611.4	14611.7
LS2-1,5 (Trimer) + 1 Yb	14625.5	14625.7
LS2-1,5 (Trimer) + 2 Tb	14770.3	14770.7
LS2-1,5 (Trimer) + 1 Tb + 1 Yb	14784.5	14784.6
LS2-1,5 (Trimer) + 2 Yb	14798.6	14798.6

45. Figure S36

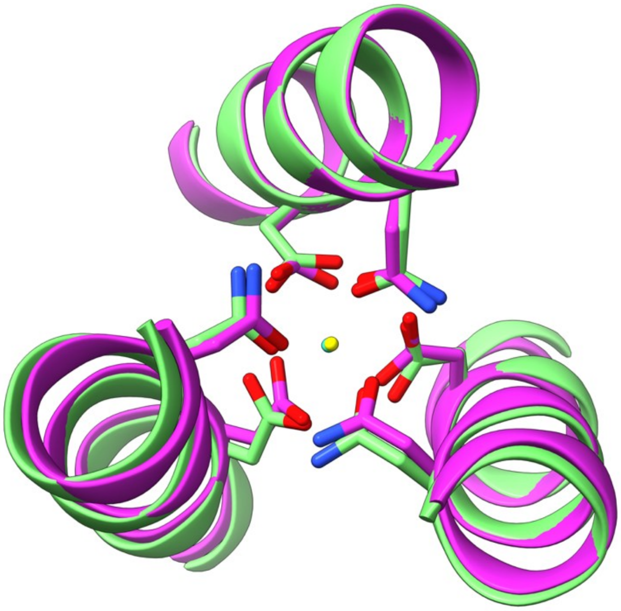

Figure S36. Structural overlay of the Asn_3Asp_3 Tb^{3+}-binding site from the previously reported structure (PDB ID: 7P3H, magenta; Tb^{3+} yellow) and the current structure (green; Tb^{3+} cyan), highlighting the conserved geometry of the metal coordination environment. Side chains involved in Tb^{3+} coordination (C magenta/green, O red, N blue) adopt highly similar conformations across both structures.

46. References

1. Chan, W. C.; White, P. D., *Fmoc Solid Phase Peptide Synthesis: A Practical Approach*. 1st ed.; Oxford University Press: New York, 2002.
2. Peacock, A. F. A.; Bullen, G. A.; Gethings, L. A.; Williams, J. P.; Kriel, F. H.; Coates, J., Gold-phosphine binding to de novo designed coiled coil peptides. *J. Inorg. Biochem.* **2012,** *117*, 298-305.
3. Berwick, M. R.; Slope, L. N.; Smith, C. F.; King, S. M.; Newton, S. L.; Gillis, R. B.; Adams, G. G.; Rowe, A. J.; Harding, S. E.; Britton, M. M.; Peacock, A. F. A., Location dependent coordination chemistry and MRI relaxivity, in de novo designed lanthanide coiled coils. *Chem. Sci.* **2016,** *7* (3), 2207-2216.
4. McKnelly, K. J.; Sokol, W.; Nowick, J. S., Anaphylaxis Induced by Peptide Coupling Agents: Lessons Learned from Repeated Exposure to HATU, HBTU, and HCTU. *The Journal of Organic Chemistry* **2020,** *85* (3), 1764-1768.
5. Borghesani, V., Uronium peptide coupling agents: Another case of occupational airborne allergic sensitization induced by HBTU. *Journal of Peptide Science* **2025,** *31* (1), e3649.
6. Barge, A.; Cravotto, G.; Gianolio, E.; Fedeli, F., How to determine free Gd and free ligand in solution of Gd chelates. A technical note. *Contrast Media Mol. Imaging* **2006,** *1* (5), 184-188.
7. Berwick, M. R.; Lewis, D. J.; Jones, A. W.; Parslow, R. A.; Dafforn, T. R.; Cooper, H. J.; Wilkie, J.; Pikramenou, Z.; Britton, M. M.; Peacock, A. F. A., De Novo Design of Ln(III) Coiled Coils for Imaging Applications. *J. Am. Chem. Soc.* **2014,** *136* (4), 1166-1169.
8. Myers, J. K.; Pace, C. N.; Scholtz, J. M., A direct comparison of helix propensity in proteins and peptides. *Proc. Natl. Acad. Sci. U.S.A.* **1997,** *94* (7), 2833-2837.
9. Beeby, A.; M. Clarkson, I.; S. Dickins, R.; Faulkner, S.; Parker, D.; Royle, L.; S. De Sousa, A.; A. Gareth Williams, J.; Woods, M., Non-radiative deactivation of the excited states of europium, terbium and ytterbium complexes by proximate energy-matched OH, NH and CH oscillators: an improved luminescence method for establishing solution hydration states. *J. Chem. Soc. Perkin Trans. 2* **1999,** *2* (3), 493-504.
10. Tochtrop, G. P.; Richter, K.; Tang, C.; Toner, J. J.; Covey, D. F.; Cistola, D. P., Energetics by NMR: Site-specific binding in a positively cooperative system. *Proceedings of the National Academy of Sciences* **2002,** *99* (4), 1847-1852.
11. Martin, R. E.; Pannier, M.; Diederich, F.; Gramlich, V.; Hubrich, M.; Spiess, H. W., Determination of end-to-end distances in a series of TEMPO diradicals of up to 2.8 nm length with a new four-pulse double electron electron resonance experiment. *Angewandte Chemie International Edition* **1998,** *37* (20), 2833-2837.
12. Milov, A.; Salikhov, K.; Shirov, M., Application of ELDOR in electron-spin echo for paramagnetic center space distribution in solids. *Fizika Tverdogo Tela* **1981,** *23* (4), 975-982.
13. Schiemann, O.; Heubach, C. A.; Abdullin, D.; Ackermann, K.; Azarkh, M.; Bagryanskaya, E. G.; Drescher, M.; Endeward, B.; Freed, J. H.; Galazzo, L.; Goldfarb, D.; Hett, T.; Esteban Hofer, L.; Fábregas Ibáñez, L.; Hustedt, E. J.; Kucher, S.; Kuprov, I.; Lovett, J. E.; Meyer, A.; Ruthstein, S.; Saxena, S.; Stoll, S.; Timmel, C. R.; Di Valentin, M.; McHaourab, H. S.; Prisner, T. F.; Bode, B. E.; Bordignon, E.; Bennati, M.; Jeschke, G., Benchmark Test and Guidelines for DEER/PELDOR Experiments on Nitroxide-Labeled Biomolecules. *Journal of the American Chemical Society* **2021,** *143* (43), 17875-17890.
14. Giannoulis, A.; Ben-Ishay, Y.; Goldfarb, D., Chapter Eight - Characteristics of Gd(III) spin labels for the study of protein conformations. In *Methods in Enzymology*, Cotruvo, J. A., Ed. Academic Press: 2021; Vol. 651, pp 235-290.
15. Cruickshank, P. A. S.; Bolton, D. R.; Robertson, D. A.; Hunter, R. I.; Wylde, R. J.; Smith, G. M., A kilowatt pulsed 94 GHz electron paramagnetic resonance spectrometer with high concentration

sensitivity, high instantaneous bandwidth, and low dead time. *Review of Scientific Instruments* **2009,** *80* (10), 103102.
16. El Mkami, H.; Hunter, R. I.; Cruickshank, P. A. S.; Taylor, M. J.; Lovett, J. E.; Feintuch, A.; Qi, M.; Godt, A.; Smith, G. M., High-sensitivity Gd3+–Gd3+ EPR distance measurements that eliminate artefacts seen at short distances. *Magn. Reson.* **2020,** *1* (2), 301-313.
17. Pannier, M.; Veit, S.; Godt, A.; Jeschke, G.; Spiess, H. W., Dead-Time Free Measurement of Dipole–Dipole Interactions between Electron Spins. *Journal of Magnetic Resonance* **2000,** *142* (2), 331-340.
18. Milov, A. D.; Ponomarev, A. B.; Tsvetkov, Y. D., Electron-electron double resonance in electron spin echo: Model biradical systems and the sensitized photolysis of decalin. *Chemical Physics Letters* **1984,** *110* (1), 67-72.
19. Mocanu, E. M.; Ben-Ishay, Y.; Topping, L.; Fisher, S. R.; Hunter, R. I.; Su, X.-C.; Butler, S. J.; Smith, G. M.; Goldfarb, D.; Lovett, J. E., Robustness and Sensitivity of Gd(III)–Gd(III) Double Electron–Electron Resonance (DEER) Measurements: Comparative Study of High-Frequency EPR Spectrometer Designs and Spin Label Variants. *Applied Magnetic Resonance* **2025,** *56* (5), 591-611.
20. Jeschke, G.; Chechik, V.; Ionita, P.; Godt, A.; Zimmermann, H.; Banham, J.; Timmel, C.; Hilger, D.; Jung, H., DeerAnalysis2006—a comprehensive software package for analyzing pulsed ELDOR data. *Applied Magnetic Resonance* **2006,** *30* (3-4), 473-498.
21. Fábregas Ibáñez, L.; Jeschke, G.; Stoll, S., DeerLab: a comprehensive software package for analyzing dipolar electron paramagnetic resonance spectroscopy data. *Magn. Reson.* **2020,** *1* (2), 209-224.
